# Association of BMI with overall and cause-specific mortality: a population-based cohort study of 3·6 million adults in the UK

**DOI:** 10.1016/S2213-8587(18)30288-2

**Published:** 2018-12

**Authors:** Krishnan Bhaskaran, Isabel dos-Santos-Silva, David A Leon, Ian J Douglas, Liam Smeeth

**Affiliations:** a Department of Non-Communicable Disease Epidemiology, London School of Hygiene & Tropical Medicine, London, UK; b Department of Community Medicine, The Arctic University of Norway, Tromsø, Norway

## Abstract

**Background:**

BMI is known to be strongly associated with all-cause mortality, but few studies have been large enough to reliably examine associations between BMI and a comprehensive range of cause-specific mortality outcomes.

**Methods:**

In this population-based cohort study, we used UK primary care data from the Clinical Practice Research Datalink (CPRD) linked to national mortality registration data and fitted adjusted Cox regression models to examine associations between BMI and all-cause mortality, and between BMI and a comprehensive range of cause-specific mortality outcomes (recorded by International Classification of Diseases, 10th revision [ICD-10] codes). We included all individuals with BMI data collected at age 16 years and older and with subsequent follow-up time available. Follow-up began at whichever was the latest of: start of CPRD research-standard follow up, the 5-year anniversary of the first BMI record, or on Jan 1, 1998 (start date for death registration data); follow-up ended at death or on March 8, 2016. Fully adjusted models were stratified by sex and adjusted for baseline age, smoking, alcohol use, diabetes, index of multiple deprivation, and calendar period. Models were fitted in both never-smokers only and the full study population. We also did an extensive range of sensitivity analyses. The expected age of death for men and women aged 40 years at baseline, by BMI category, was estimated from a Poisson model including BMI, age, and sex.

**Findings:**

3 632 674 people were included in the full study population; the following results are from the analysis of never-smokers, which comprised 1 969 648 people and 188 057 deaths. BMI had a J-shaped association with overall mortality; the estimated hazard ratio per 5 kg/m^2^ increase in BMI was 0·81 (95% CI 0·80–0·82) below 25 kg/m^2^ and 1·21 (1·20–1·22) above this point. BMI was associated with all cause of death categories except for transport-related accidents, but the shape of the association varied. Most causes, including cancer, cardiovascular diseases, and respiratory diseases, had a J-shaped association with BMI, with lowest risk occurring in the range 21–25 kg/m^2^. For mental and behavioural, neurological, and accidental (non-transport-related) causes, BMI was inversely associated with mortality up to 24–27 kg/m^2^, with little association at higher BMIs; for deaths from self-harm or interpersonal violence, an inverse linear association was observed. Associations between BMI and mortality were stronger at younger ages than at older ages, and the BMI associated with lowest mortality risk was higher in older individuals than in younger individuals. Compared with individuals of healthy weight (BMI 18·5–24·9 kg/m^2^), life expectancy from age 40 years was 4·2 years shorter in obese (BMI ≥30·0 kg/m^2^) men and 3·5 years shorter in obese women, and 4·3 years shorter in underweight (BMI <18·5 kg/m^2^) men and 4·5 years shorter in underweight women. When smokers were included in analyses, results for most causes of death were broadly similar, although marginally stronger associations were seen among people with lower BMI, suggesting slight residual confounding by smoking.

**Interpretation:**

BMI had J-shaped associations with overall mortality and most specific causes of death; for mental and behavioural, neurological, and external causes, lower BMI was associated with increased mortality risk.

**Funding:**

Wellcome Trust.

## Introduction

High BMI contributed to an estimated 4 million deaths globally in 2015.^1^ Several major studies and meta-analyses have found strong associations between BMI and all-cause mortality; most have described a U-shaped association with minimum mortality in the healthy weight (20–25 kg/m^2^) range;^2^, ^3^, ^4^, ^5^ a 2013 meta-analysis suggested that overweight might be protective^6^ but concerns were raised about whether the study had adequately accounted for age, reverse causality, and confounding by smoking.^7^ A recent large meta-analysis, which explored the effect of different methodological decisions on results, observed a higher nadir of the BMI–mortality curve when studies with short follow-up (and thus increased susceptibility to reverse causality) were included, and when ever-smokers were included.^2^

There is less evidence about the associations between BMI and cause-specific mortality outcomes. Among studies investigating cardiovascular mortality, increased BMI has generally been associated with increased risk,^4^, ^5^, ^8^, ^9^ but it is unclear whether risk begins to increase with overweight^5^ or only with obesity,^10^ and whether underweight affects risk.^4^, ^9^ U-shaped or J-shaped associations have been found between BMI and all-cancer mortality^4^ but there is evidence of variation by cancer site based on studies looking at a range of site-specific cancers.^11^ Drawing out patterns from existing evidence is complicated by variation in study settings and populations; in analytical strategy, including inclusion and exclusion of smokers, inclusion and exclusion of early follow-up time, and handling of pre-existing disease; and in the range and granularity of outcomes considered.

**Research in context**

**Evidence before the study**

Two meta-analyses published in 2016 reviewed studies of associations between BMI and all-cause mortality. The first found a J-shaped association with lowest risk at a BMI of 23–24 kg/m^2^ among never-smokers. Inclusion of smokers and people with existing but undiagnosed illnesses were identified as important potential sources of bias. The second, by the Global BMI Mortality Collaboration, was an individual patient data meta-analysis and found a similar pattern, largely consistent across four continents. In the absence of any broad systematic reviews examining the association of BMI and cause-specific mortality outcomes, we searched PubMed for articles published in the past 10 years (Jan 1, 2007, to Dec 31, 2017) and retrieved 67 studies that investigated associations between BMI (as a continuous variable or in at least three categories) and one or more cause-specific mortality outcomes. The search string was (“body mass index” OR bmi OR obes*OR overweight) AND (mortality OR death); inclusion and exclusion criteria are listed in the appendix. The 67 included studies are described in the appendix; deaths from cardiovascular disease, cancer, and respiratory disease were most often studied, whereas only a small number of studies investigated deaths from other causes—namely, diabetes, external causes, liver or digestive diseases, kidney diseases, and infectious diseases. Only six studies investigated four or more of these categories of causes. Findings for cause-specific mortality outcomes from studies based in European, North American, Australian, and trans-continental settings (probably the most comparable to our data) are summarised in the appendix. Positive or J-shaped associations were observed for most cardiovascular disease mortality outcomes; associations between BMI and cancer mortality were smaller overall but varied by cancer site; inverse or U-shaped associations were reported for respiratory deaths, and for other outcomes there was limited evidence. Analogous results from 30 studies in Asian settings are also summarised in the appendix.

**Added value of this study**

To the best of our knowledge, this is one of the largest single cohort studies of its kind to date that quantifies the associations between BMI and a comprehensive range of mortality outcomes at three levels of granularity, including several outcomes for which few previous data are available. We built on previous evidence by using flexible spline models to examine non-linearity in detail, and we also investigated potential effect modification. We used consistent methodology throughout to minimise confounding and reverse causality, and did extensive sensitivity analyses.

**Implications of all the available evidence**

Important associations exist between BMI and almost every category of mortality outcome. In contrast with some previous evidence suggesting that overweight might be protective, we found that risk began to increase above 21–25 kg/m^2^ for most outcomes, including all-cause mortality, cardiovascular disease, and cancer. However, for mental and behavioural, neurological, and external causes, only lower BMI was associated with increased risk. We found strong evidence of effect modification by age; further work is needed to explore the drivers of this effect and thus understand whether healthy weight recommendations might need to take age into consideration.

We aimed to examine in detail the association of BMI with all-cause and cause-specific mortality outcomes using a large, single, contemporary, population-based cohort. We applied a consistent approach to minimise reverse causality and residual confounding. We also investigated effect modification by key individual-level characteristics and estimated absolute effects of BMI on mortality outcomes.

## Methods

### Study design and setting

We did a cohort study using prospectively collected data from the UK Clinical Practice Research Datalink (CPRD) linked to national death registration data. The CPRD contains primary care records from general practitioners (GPs) covering around 9% of the UK population; linkage to death registration data, including the date and causes of death, was available for 80% of GP clinics in England. CPRD data, including the linked subset, have been shown to be broadly representative of the general population in terms of age, sex, ethnicity, and BMI.^12^, ^13^, ^14^, ^15^

The study protocol was approved by the London School of Hygiene & Tropical Medicine Ethics Committee (14389) and the Independent Scientific Advisory Committee for MHRA Database Research (protocol number 16_174, approved Aug 24, 2016, and provided in the appendix).

### Participants, exposures, and outcomes

We included all individuals with BMI data collected at age 16 years and older and with subsequent follow-up time available; BMI data were processed as described elsewhere.^12^ Exposure was assigned as the earliest BMI recorded during CPRD research-standard follow-up (appendix);^14^ in the absence of BMI recorded at the start of follow-up, we used the most recent historical BMI record (if available) and updated it at the date of the first BMI during follow-up.

To minimise reverse causality (disease leading to weight change), we excluded the first 5 years of follow-up after the BMI record. Follow-up began at whichever was the latest of: start of CPRD research-standard follow-up, the 5-year anniversary of the first BMI record, or on Jan 1, 1998 (start date for death registration data); follow-up ended at death or on March 8, 2016 (end date for death registration data).

The outcomes were all-cause mortality and cause-specific mortality based on the International Classification of Diseases, 10th revision (ICD-10) code recorded as the underlying cause of death. We used a three-level hierarchical classification of causes of death as used by the Global Burden of Diseases, Injuries, and Risk Factors Study.^16^ We studied all Level 1 outcomes (communicable diseases, non-communicable diseases, and injuries and external causes); all Level 2 non-communicable disease outcomes (high-level disease groupings such as cancer and cardiovascular disease), and selected Level 3 outcomes (specific disease and injury types, such as lung cancer and heart failure) that were either common causes of death in the UK,^17^ or were a priori expected to have important associations with BMI (the full list of outcomes with ICD codes is provided in the appendix).

### Statistical analysis

Cox regression models with an age timescale were fitted for all-cause mortality and for each cause-specific mortality outcome, censoring deaths from competing causes.^18^ BMI was initially fitted in WHO categories,^19^ then in finer BMI categories as used by the Global BMI Mortality Collaboration (to aid comparison with work based on this classification),^4^ and then as a restricted cubic spline (smooth curve). Fully adjusted models were stratified by sex and adjusted for baseline age, smoking, alcohol use, diabetes, index of multiple deprivation (a measure of socioeconomic status^20^), and calendar period. We excluded individuals with missing smoking status (n=25 373 [0·7%]) or alcohol status (n=244 848 [6·7%]). Further details about parametrisation of covariates are provided in the appendix. Simpler linear or piecewise-linear models were next fitted for the Level 1 and Level 2 outcomes to quantify associations: where there was evidence of non-linearity, a two-line piecewise linear model with a single change point was estimated by trying all possible values for the change point and choosing the value with highest likelihood.

We fitted interactions to investigate effect modification by sex, current age, smoking, index of multiple deprivation quintile, and (among those with available data) ethnicity. To quantify absolute effects, we estimated the expected age of death for men and women aged 40 years by BMI category using a simplified Poisson model including BMI category, age, sex, and interactions between these variables. Age 40 years was chosen as this was the approximate median age at entry and there were few deaths at younger ages; full details of the methods are provided in the appendix. Population attributable fractions were calculated by combining hazard ratios (HRs) from the nine-category BMI model with observed deaths in each BMI category.^21^ Cumulative incidences of mortality from cardiovascular disease, cancer, neurological causes, and respiratory causes were also calculated by BMI category by use of competing risks methods.^18^

### Sensitivity analyses

First, we varied the amount of initial follow-up time after the BMI record that was excluded between 0 years and 10 years; in the primary analysis the first 5 years were excluded to minimise reverse causality. Second, we excluded individuals with prevalent cancer or cardiovascular disease at the start of follow-up for those respective mortality outcomes, to further explore possible reverse causality; we also excluded individuals with previous chronic obstructive pulmonary disorder from the respiratory mortality analysis; dementia or Alzheimer's disease from the neurological mortality analysis; and depression, bipolar disorder, or schizophrenia from the analyses of deaths from mental and behavioural causes and from self-harm and interpersonal violence. Third, we considered alternative non-linear parametrisations of BMI—namely, very fine categories of 1 kg/m^2^ width (18·0–18·9 kg/m^2^, 19·0–19·9 kg/m^2^, and so on), and a two-term fractional polynomial.^22^ Fourth, we adjusted for ethnicity where such data were available. Fifth, we dropped adjustment for diabetes, then excluded patients with diabetes entirely, in case diabetes might act as an intermediary. Sixth, we dropped BMIs recorded before the start of research-standard CPRD follow-up. Seventh, we restricted the analysis to patients who had a BMI record less than 12 months after registration (more likely because of administrative reasons rather than clinically motivated). Finally, we explored the effect of missing BMI by restricting analyses to more recent calendar periods in which BMI completeness was higher.

### Role of the funding source

The sponsor had no role in study design, data collection, data analysis, data interpretation, or writing of the report. The corresponding author had full access to all the data in the study and had final responsibility for the decision to submit for publication.

## Results

Of data from 8 093 746 individuals from CPRD practices in England, 3 632 674 were included in the study after excluding data from individuals with no linked mortality data, BMI data, or BMI data outside of the plausible range (15–50 kg/m^2^), and from individuals followed up for less than 5 years after BMI measure (appendix). Of individuals included in the study, 1 969 648 were never-smokers. 367 512 deaths were observed (188 057 among never-smokers; table 1; appendix). A positive association was observed between age and BMI (median age 25 years in underweight individuals, 33 years in healthy-weight individuals, 42 years in overweight individuals, and 43 years in obese individuals), in keeping with expected trends;^23^ 70·3% of underweight individuals were women, whereas 56·1% of obese individuals were men (table 1).

**Table 1:** Characteristics of study population at the time of BMI measure by WHO BMI category,^19^ restricted to individuals with follow-up available from 5 years after the BMI record

		**Underweight (<18·5 kg/m^2^); n=112 077**	**Healthy weight (18·5–24·9 kg/m^2^); n=1 793 989**	**Overweight (25·0–29·9 kg/m^2^); n=1 151 359**	Obese (≥30·0 kg/m^2^); n=575 249*	**Overall; n=3 632 674**
**Time from BMI record date to end of follow-up (years)**						
	Mean (SD)	12·2 (5·4)	13·1 (5·6)	12·6 (5·4)	11·5 (5·1)	12·7 (5·5)
	Median (IQR)	11 (7·8–15·6)	12 (8·4–17·2)	11·6 (8·1–16·1)	10·4 (7·4–14·4)	11·6 (8·1–16·3)
	Total follow-up included (millions of person-years)	0·756	13·614	8·248	3·557	26·176
Age (years)						
	<30	73 653 (65·7%)	769 152 (42·9%)	271 837 (23·6%)	124 965 (21·7%)	1 239 607 (34·1%)
	30–39	15 780 (14·1%)	394 672 (22·0%)	253 964 (22·1%)	123 093 (21·4%)	787 509 (21·7%)
	40–49	7529 (6·7%)	243 730 (13·6%)	211 796 (18·4%)	116 511 (20·3%)	579 566 (16·0%)
	50–59	4531 (4·0%)	163 210 (9·1%)	178 411 (15·5%)	99 176 (17·2%)	445 328 (12·3%)
	60–69	4221 (3·8%)	117 085 (6·5%)	137 148 (11·9%)	70 164 (12·2%)	328 618 (9·0%)
	70–79	3937 (3·5%)	76 051 (4·2%)	76 680 (6·7%)	33 964 (5·9%)	190 632 (5·2%)
	≥80	2426 (2·2%)	30 089 (1·7%)	21 523 (1·9%)	7376 (1·3%)	61 414 (1·7%)
	Median (IQR)	24·7 (19·2–35·5)	32·7 (24·3–47·0)	42·2 (30·6–56·8)	43·3 (31·6–56·5)	36·9 (26·6–52·4)
Sex						
	Women	78 745 (70·3%)	1 068 110 (59·5%)	518 896 (45·1%)	322 647 (56·1%)	1 988 398 (54·7%)
	Men	33 332 (29·7%)	725 879 (40·5%)	632 463 (54·9%)	252 602 (43·9%)	1 644 276 (45·3%)
Smoking status						
	Never-smoker	59 327 (52·9%)	989 704 (55·2%)	617 698 (53·6%)	302 919 (52·7%)	1 969 648 (54·2%)
	Current smoker	41 272 (36·8%)	559 478 (31·2%)	312 427 (27·1%)	147 707 (25·7%)	1 060 884 (29·2%)
	Ex-smoker	9828 (8·8%)	231 269 (12·9%)	214 542 (18·6%)	121 130 (21·1%)	576 769 (15·9%)
	Data missing	1650 (1·5%)	13 538 (0·8%)	6692 (0·6%)	3493 (0·6%)	25 373 (0·7%)
Alcohol use						
	Non-drinker	27 058 (24·1%)	277 594 (15·5%)	169 072 (14·7%)	105 930 (18·4%)	579 654 (16·0%)
	Current drinker, 1–2 units per day	56 496 (50·4%)	1 083 931 (60·4%)	674 329 (58·6%)	317 722 (55·2%)	2 132 478 (58·7%)
	Current drinker, 3–6 units per day	5337 (4·8%)	170 458 (9·5%)	148 318 (12·9%)	56 156 (9·8%)	380 269 (10·5%)
	Current drinker, ≥7 units per day	1835 (1·6%)	28 469 (1·6%)	21 958 (1·9%)	11 634 (2·0%)	63 896 (1·8%)
	Current drinker, unknown level	5250 (4·7%)	76 572 (4·3%)	47 688 (4·1%)	25 650 (4·5%)	155 160 (4·3%)
	Ex-drinker	2396 (2·1%)	31 974 (1·8%)	24 398 (2·1%)	17 601 (3·1%)	76 369 (2·1%)
	Data missing	13 705 (12·2%)	124 991 (7·0%)	65 596 (5·7%)	40 556 (7·1%)	244 848 (6·7%)
Any previous diabetes diagnosis		885 (0·8%)	25 896 (1·4%)	38 903 (3·4%)	39 323 (6·8%)	105 007 (2·9%)
Index of multiple deprivation						
	Quintile 1 (low)	21 735 (19·4%)	428 458 (23·9%)	260 902 (22·7%)	102 681 (17·8%)	813 776 (22·4%)
	Quintile 2	21 840 (19·5%)	392 644 (21·9%)	255 758 (22·2%)	115 817 (20·1%)	786 059 (21·6%)
	Quintile 3	23 046 (20·6%)	374 109 (20·9%)	242 194 (21·0%)	121 034 (21·0%)	760 383 (20·9%)
	Quintile 4	22 960 (20·5%)	329 436 (18·4%)	214 904 (18·7%)	120 965 (21·0%)	688 265 (18·9%)
	Quintile 5 (high)	22 324 (19·9%)	267 155 (14·9%)	176 289 (15·3%)	114 098 (19·8%)	579 866 (16·0%)
Ethnicity						
	White	36 317 (32·4%)	619 968 (34·6%)	428 065 (37·2%)	235 071 (40·9%)	1 319 421 (36·3%)
	South Asian	5432 (4·8%)	48 835 (2·7%)	28 318 (2·5%)	10 835 (1·9%)	93 420 (2·6%)
	Black	1474 (1·3%)	23 675 (1·3%)	20 772 (1·8%)	14 292 (2·5%)	60 213 (1·7%)
	Other	2244 (2·0%)	21 529 (1·2%)	10 279 (0·9%)	4170 (0·7%)	38 222 (1·1%)
	Mixed	648 (0·6%)	8357 (0·5%)	4499 (0·4%)	2374 (0·4%)	15 878 (0·4%)
	Data missing	65 962 (58·9%)	1 071 625 (59·7%)	659 426 (57·3%)	308 507 (53·6%)	2 105 520 (58·0%)
Calendar year of first available BMI record						
	<1989	803 (0·7%)	18 480 (1·0%)	11 300 (1·0%)	4414 (0·8%)	34 997 (1·0%)
	1990–94	16 549 (14·8%)	379 351 (21·1%)	233 344 (20·3%)	83 708 (14·6%)	712 952 (19·6%)
	1995–99	22 241 (19·8%)	400 716 (22·3%)	252 260 (21·9%)	111 507 (19·4%)	786 724 (21·7%)
	2000–04	28 235 (25·2%)	416 163 (23·2%)	279 589 (24·3%)	153 742 (26·7%)	877 729 (24·2%)
	2005–09	36 201 (32·3%)	479 627 (26·7%)	311 622 (27·1%)	181 477 (31·5%)	1 008 927 (27·8%)
	≥2010	8048 (7·2%)	99 652 (5·6%)	63 244 (5·5%)	40 401 (7·0%)	211 345 (5·8%)

Data are n (%), mean (SD), or median (IQR).

* Among 575 249 obese individuals, 405 005 (70·4%), had obesity class 1 (BMI 30–34·9 kg/m^2^), 121 891 (21·2%) had obesity class 2 (BMI 35–39·9 kg/m^2^), and 48 353 (8·4%) had obesity class 3 (BMI ≥40 kg/m^2^). A further breakdown of these characteristics by sex as well as BMI category is given in the appendix. Characteristics are at the time of the first BMI record used in study where applicable; smoking was assigned by use of the record from same date as the BMI record or within 1 year before where available (for 3 000 050 [83%] patients), or by use of the nearest record in year after the BMI record (160 790 [4%]), or by use of the nearest record >1 year before the BMI record (285 817 [8%]), or by use of the nearest record >1 year after the BMI record (160 427 [4%]); a similar algorithm was used for alcohol; for ethnicity, the earliest available record was used.

**Figure 1:**
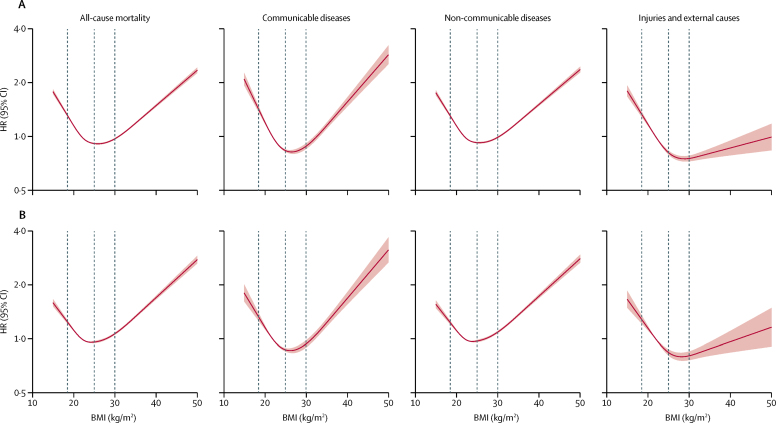
All-cause mortality and Level 1 cause-specific mortality outcomes in total study population (A) and in never-smokers only (B) We used a three-level hierarchical classification of causes of death as used by the Global Burden of Diseases, Injuries, and Risk Factors Study.^16^ All Level 1 outcomes (communicable diseases, non-communicable diseases, and injuries and external causes) were studied. 5-year exclusion period applied for person-time and events after a BMI record. Dashed vertical lines represent WHO BMI category thresholds of 18·5 kg/m^2^ (underweight to healthy), 25 kg/m^2^ (healthy weight to overweight), and 30 kg/m^2^ (overweight to obese). Estimates adjusted for age at BMI record, deprivation, calendar year, diabetes, alcohol status, and smoking (all as defined at date of BMI measure) and stratified for sex. The p values for overall association and p values for non-linearity were less than 0·0001 for all outcomes, in both full and never-smoker populations. HR=hazard ratio.

Associations between BMI and mortality were J-shaped for all-cause, communicable, and non-communicable disease mortality. For injuries and external causes, a marked increase in risk was observed at low BMIs, but minimal elevation in risk at higher BMIs (figure 1). Restriction to never-smokers slightly attenuated the association at low BMIs for all these outcomes (figure 1) and also for cancer mortality (appendix). The nadir for all-cause mortality risk among never-smokers was estimated from piecewise linear models to be at a BMI of 25 kg/m^2^ (table 2).

**Table 2:** Estimated change points in the association between BMI and mortality among never-smokers, and associations with mortality below and above the change point, from piecewise two-line models for the 5-year post-BMI exclusion period

		**BMI change point, kg/m^2^ (95% CI)**	HR per 5 kg/m^2^ BMI increase below change point*(95% CI)	**HR per 5 kg/m^2^ BMI increase above change point (95% CI)**
All-cause mortality		25 (25–25)	0·81 (0·80–0·82)	1·21 (1·20–1·22)
Level 1 outcomes				
	Communicable diseases	26 (26–26)	0·73 (0·71–0·76)	1·28 (1·24–1·31)
	Non-communicable diseases	25 (25–25)	0·83 (0·81–0·84)	1·22 (1·21–1·23)
	Injuries and external causes	27 (26–28)	0·75 (0·71–0·80)	1·10 (1·04–1·17)
Level 2 outcomes (ICD-10 chapters/codes)				
	Cancers (C)	21 (20–25)	0·88 (0·80–0·97)	1·13 (1·12–1·14)
	Blood and endocrine (D50–89, E)	22 (22–29)	0·43 (0·35–0·54)	1·42 (1·37–1·48)
	Mental and behavioural (F)	24 (21–25)	0·31 (0·22–0·44)	1·05 (0·86–1·27)
	Neurological (G)	26 (25–27)	0·68 (0·66–0·70)	0·98 (0·96–1·01)
	Cardiovascular (I)	25 (25–25)	0·89 (0·87–0·91)	1·29 (1·27–1·30)
	Respiratory (J23–99)	25 (24–25)	0·53 (0·50–0·56)	1·25 (1·21–1·29)
	Liver cirrhosis (K70·3/71·7/74·3–6)	23 (22–27)	0·75 (0·48–1·16)	1·44 (1·33–1·55)
	Digestive (K, excluding cirrhosis)	24 (22–25)	0·79 (0·72–0·86)	1·32 (1·28–1·36)
	Musculoskeletal (M)	24 (24–25)	0·45 (0·39–0·53)	1·23 (1·15–1·32)
	Urogenital (N)	25 (24–25)	0·84 (0·77–0·93)	1·45 (1·39–1·51)
	Accident, transport-related (V)	NA*	1·00 (0·90–1·11)	..
	Accident, excluding transport (W/X00–59)	27 (26–28)	0·71 (0·66–0·77)	1·17 (1·09–1·26)
	Self-harm and interpersonal violence (X60–Y09)	NA*	0·87 (0·80–0·94)	..

HR=hazard ratio. ICD-10=International Classification of Diseases, 10th revision. NA=not available.

**Figure 2:**
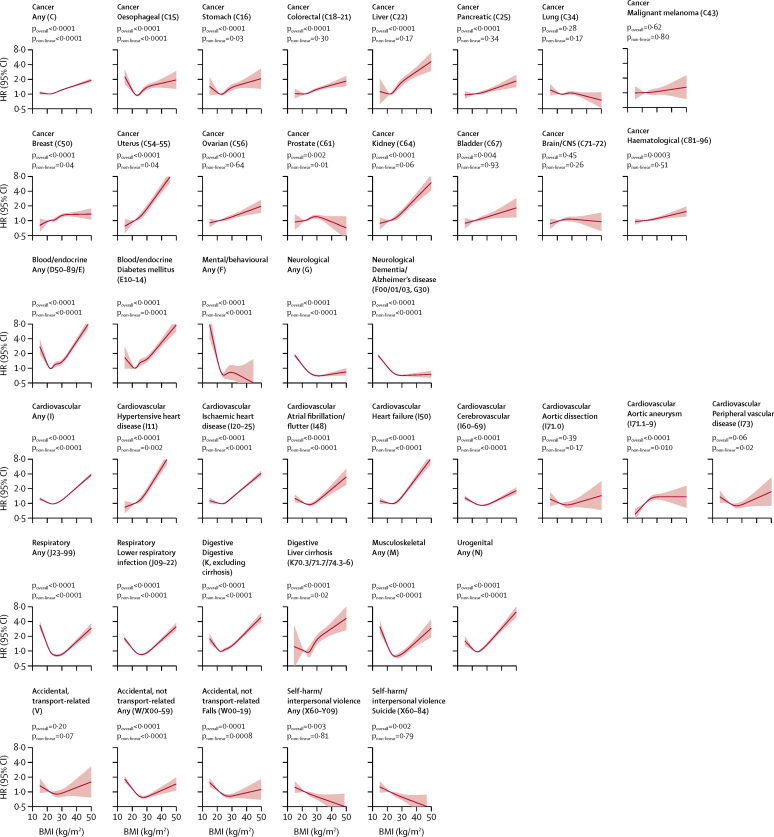
Association between BMI and Level 2 and Level 3 cause-specific mortality outcomes among never-smokers (organised by ICD-10 code) We used a three-level hierarchical classification of causes of death as used by the Global Burden of Diseases, Injuries, and Risk Factors Study.^16^ We studied all Level 2 non-communicable disease outcomes and selected Level 3 outcomes that were either common causes of death in the UK or were a priori expected to have important associations with BMI. 5-year exclusion period applied for person-time and events after a BMI record; estimates adjusted for age, deprivation, calendar year, diabetes, alcohol status (all as defined at date of BMI measure) and stratified for sex. HR=hazard ratio. ICD-10=International Classification of Diseases, 10th revision.

* For transport-related accidents, and self-harm and interpersonal violence, there was little or no evidence against linearity (figure 2) so a single linear effect without change point was estimated.

Estimated associations between BMI and more specific mortality outcomes are shown from non-linear spline models (figure 2) and from linear and piecewise linear models (table 2) in never-smokers (analagous results in the full study population including smokers are provided in the appendix). For 11 of 13 Level 2 mortality outcomes, there was evidence of non-linearity, with two main patterns seen: for eight outcomes (cancer, cardiovascular, respiratory, blood/endocrine, liver cirrhosis, other digestive, musculoskeletal, and urogenital causes), we estimated the mortality risk to reach a nadir at BMIs in the range of 21–25 kg/m^2^, with inverse associations below, and positive associations above, although the magnitude of associations varied; for three outcomes (mental and behavioural, neurological, and accidental [non-transport-related]), we found inverse associations below a BMI of 24–27 kg/m^2^, with little evidence of association at higher BMIs. We estimated a linear inverse association between BMI and deaths from self-harm and interpersonal violence (HR 0·87 per 5 kg/m^2^ increase; 95% CI 0·80–0·94); we found no evidence of association between BMI and deaths from transport-related accidents.

The overall pattern of association between BMI and cardiovascular death was similar for most Level 3 cardiovascular outcomes but was more muted for cerebrovascular deaths (figure 2). Broadly positive associations were observed between BMI and 12 of 15 site-specific cancer mortality outcomes, with evidence of non-linearity for oesophageal, stomach, uterus, prostate, and kidney cancers, generally reflecting a weak association at the lowest BMIs, although for oesophageal cancer there was a J-shaped association. BMI was not associated with deaths from lung cancer, brain or CNS cancer, or malignant melanoma.

Results from categorical BMI models are in the appendix.

**Figure 3:**
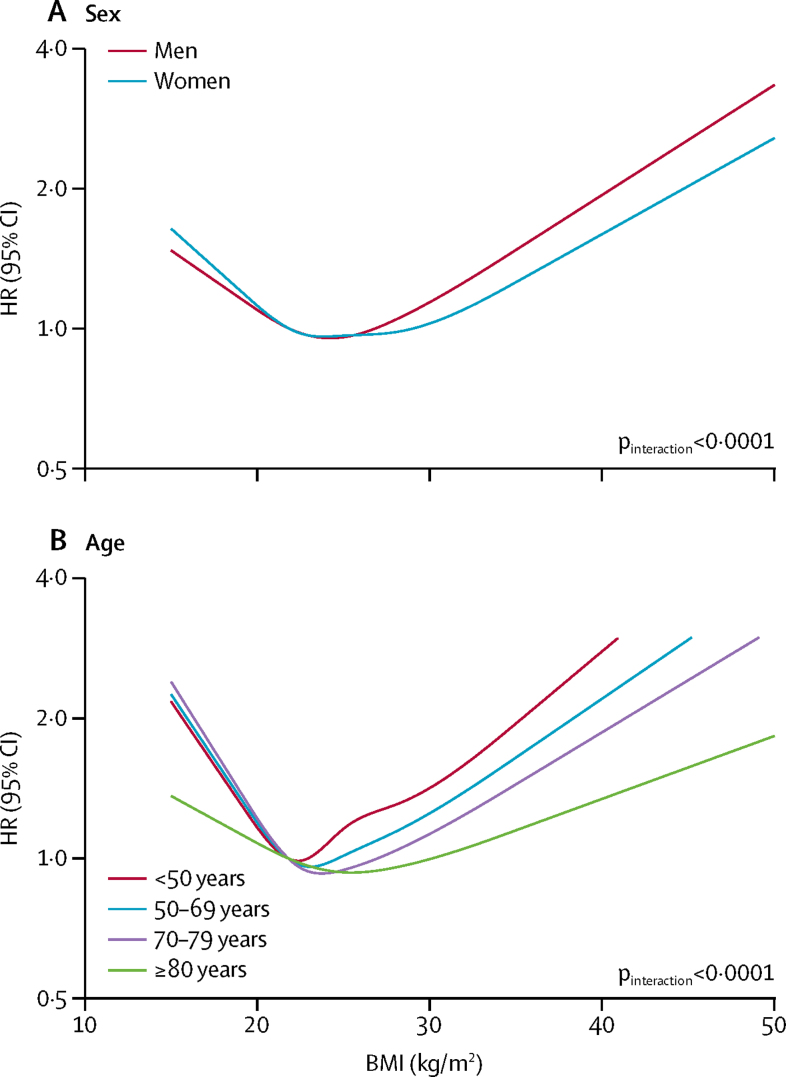
Association between BMI and all-cause mortality among never-smokers, by sex (A) and age (B) 5-year exclusion period applied for person-time and events after a BMI record; estimates adjusted for age, deprivation, calendar year, diabetes, and alcohol status (all as defined at date of BMI measure) and stratified by sex. HR=hazard ratio.

High BMI was more strongly associated with overall and cardiovascular mortality in men than in women (figure 3; appendix). Most associations between BMI and mortality were attenuated at older ages (figure 3; appendix). In a post-hoc analysis we estimated the nadir of all-cause mortality risk to be 23 kg/m^2^ at age younger than 70 years, rising to 25 kg/m^2^ at age 70 years and older. We found no evidence of effect modification by deprivation (appendix). Investigation of effect modification by ethnicity was limited by low power and we only considered all-cause mortality; despite some difference in the observed patterns by ethnicity, particularly at low BMI, there was insufficient evidence to rule out chance variation (appendix).

The expected age of death for a 40-year-old never-smoker with healthy weight was 82·2 years for men and 84·3 years for women (table 3). Underweight, overweight, and obesity were all associated with reductions in these life expectancies: obesity overall was associated with shortening of life expectancy by 4·2 years in men and by 3·5 years in women; class 3 obesity was associated with shortening of life expectancy by 9·1 years in men and by 7·7 years in women.

**Table 3:** Expected age at death for a never-smoker aged 40 years by WHO BMI category,^19^ and estimated reduction in life expectancy compared with an individual of healthy weight

		**Men**		**Women**	
		Expected age of death at age 40 years (years)	Reduction in life expectancy (years)	Expected age of death at age 40 years (years)	Reduction in life expectancy (years)
Underweight (<18·5 kg/m^2^)		77·9	4·3	79·8	4·5
Healthy weight (18·5–24·9 kg/m^2^)		82·2	..	84·3	..
Overweight (25·0–29·9 kg/m^2^)		81·2	1·0	83·5	0·8
Obese (all, ≥30·0 kg/m^2^)		78·0	4·2	80·9	3·5
	Obese class 1 (30·0–34·9 kg/m^2^)	78·7	3·4	81·9	2·4
	Obese class 2 (35·0–39·9 kg/m^2^)	76·2	5·9	79·6	4·7
	Obese class 3 (≥40·0 kg/m^2^)	73·1	9·1	76·6	7·7

Expected age of death at age 40 years estimated from a Poisson model for overall survival with six-category BMI variable, 5-year age bands, sex, and interaction terms for BMI with age at BMI, and BMI with sex (see appendix for details); estimates assume mortality observed in the study remains constant. Reduction in life expectancy is calculated as expected age of death minus expected age at death in the healthy weight category.

Assuming causality, we estimated that 4·3% of all deaths might be attributable to obesity, and 5·5% to overweight including obesity (appendix). Cumulative incidences of cause-specific mortality outcomes by BMI are shown in the appendix.

Exclusion of more initial follow-up time attenuated inverse associations at low BMI for most outcomes; exclusion of prevalent disease gave similar results to exclusion of early person-time (appendix). Other sensitivity analyses made little difference to estimated associations between BMI and mortality (appendix).

## Discussion

We observed a J-shaped association between BMI and all-cause mortality, with lowest mortality at 25 kg/m^2^. BMI was associated with mortality risk from every main category of cause except for transport-related accidents. There were three broad patterns of association: for cancer, cardiovascular, respiratory, blood and endocrine, digestive, musculoskeletal, and urogenital causes of death, we found J-shaped associations with nadirs at 21–25 kg/m^2^, and varying magnitudes of association; for mental and behavioural, neurological, and accidental (non-transport-related) causes, BMI was inversely associated with mortality up to 24–27 kg/m^2^ with little association above this point; and BMI was inversely and linearly associated with deaths from self-harm and interpersonal violence. Associations between high BMI and several mortality outcomes attenuated with age and were stronger in men than in women. Obesity was associated with a 4·2-year reduction in remaining life expectancy for a male 40-year-old never-smoker and a 3·5-year reduction for a female 40-year-old never smoker, when compared with individuals of healthy weight, with longest reductions in life expectancy estimated for the most severely obese (class 3) individuals; underweight was associated with a reduction in life expectancy of more than 4 years.

The J-shaped association we observed between BMI and all-cause mortality was consistent with results from some major studies,^2^, ^4^ but others have estimated a reduced risk among overweight individuals compared with those of healthy weight.^6^ Reverse causality and residual confounding (in particular by smoking) might partly explain the discrepant results between studies; associations between overweight and mortality are attenuated in studies with short follow-up, or in studies that include smokers.^2^ We also observed clear heterogeneity in the associations between BMI and different causes of death, and strong interactions with age, meaning that the association of all-cause mortality with BMI in any individual study will be affected by the age and cause-of-death distributions in the source population.

In the absence of broad evidence summarising cause-specific mortality outcomes, we did a systematic literature review and identified 67 relevant studies from 2007 to 2017 for comparison (appendix). Most studies either excluded an initial follow-up period of 1–10 years (n=20) or excluded people with previous disease (n=11), or excluded both (n=13). Most studies of overall cardiovascular mortality from similar settings found approximately J-shaped associations, consistent with our results (appendix); only a few examined more specific cardiovascular mortality outcomes. J-shaped associations were similarly seen for deaths from coronary heart disease and heart failure, and, in some studies, for cerebrovascular deaths;^4^, ^5^ however, more modest associations between BMI and cerebrovascular death were also observed, as in the present analysis.^24^ Ischaemic and haemorrhagic stroke were rarely distinguished, despite their potentially different associations with BMI.^5^ Most previous evidence about cancer mortality has focused on any cancer type or on breast, colorectal, lung, and prostate cancers, with less common cancers infrequently studied (appendix). Studies into prostate cancer mortality generally imposed linearity or used few BMI categories; our results suggest important non-linearity in the association, with a levelling off or reduction in risk at the highest BMIs. Various studies agreed with our finding of no association between BMI and lung cancer mortality, whereas others found both strong positive and negative associations, possibly reflecting the complications of adequately accounting for smoking. Few studies looked at mortality outcomes other than those from cardiovascular disease and cancer (appendix).

The raised risks of many outcomes at low BMI, coupled with the fact that mental health conditions showed the strongest inverse associations with low BMI, might indicate pervasive effects of mental health problems on a range of outcomes, through pathways that could include poorer self-care and less access to or use of health-care services, or both. The persistence of inverse associations between BMI and deaths from self-harm and interpersonal violence even in sensitivity analyses in which follow-up was started up to 10 years after BMI recording, or when individuals with previously recorded mental illness were excluded, argues against reverse causality. However, it remains possible that depression and related diseases leading to appetite suppression even over a long time period or without a formal diagnosis could partly explain this finding. Imposing a longer period between BMI recording and study entry tended to attenuate associations between low BMI and outcomes; this might have been observed because a side-effect of this approach is to reduce the amount of person-time included at young ages, and we separately found the strongest associations between low BMI and mortality to be in younger people. The analyses stratified by age also suggested that mortality was minimised in older individuals at higher BMI, perhaps indicating increased importance of nutritional reserves in older age. This finding might suggest that healthy weight recommendations need to account for age, but further work is needed to establish whether increased weight is actually beneficial for older individuals: there is increased risk of reverse causation in older people because of the increased prevalence of most diseases, and BMI might be compromised as a measure of adiposity in the oldest individuals because of complications from loss of muscle mass.^25^

In this study, we systematically analysed outcomes at different levels of granularity, used consistent methodology to deal with confounding and reverse causality, and did a wide range of sensitivity analyses. A particular strength was that the size of the study allowed us to retain power while restricting analyses to never-smokers and thus minimising confounding by smoking. BMI data in the CPRD have good validity and representativeness.^12^ In preliminary analyses, we validated linked mortality data by comparing these with data recorded directly in primary care records; dates of death agreed to within 1 month in 97% of cases.

25% of individuals who were otherwise eligible were excluded because they had no BMI records available. This complete case analysis approach is valid provided that absence of data is conditionally independent of the outcome under study;^26^ we considered this approach more appropriate than multiple imputation, because underweight and overweight individuals would be more likely to have their BMI recorded in primary care, contradicting the required missing at random assumption.^27^ Several sensitivity analyses suggested that missing BMI data had little effect on our estimates: there was little change in results when analyses were restricted to more recent calendar years, despite BMI data completeness increasing from 66% to 80% in 2000–15. There might have been inaccuracies in cause of death recording. Different physicians could differently interpret the underlying cause of death, and certification of deaths in hospital could be completed by one of several doctors in a team, increasing the risk of classification errors.^28^ Some causes of death might be particularly prone to misclassification: we noted a relatively large number of deaths attributed to pneumonia, which might have been secondary to other morbidities. Clear national guidance about certification should have helped reduce errors.^28^ If any misclassification was unrelated to BMI, the expected impact on our analysis would be loss of power. We might also have missed deaths that were not registered in the UK because of emigration, but these are likely to represent a small proportion of deaths. We had no data about diet, physical activity, or cardiorespiratory fitness, with which we could have further explored causal pathways and confounding,^29^, ^30^ and we had no data about measures of adiposity other than BMI.

In conclusion, BMI had a J-shaped association with overall mortality, and BMI outside the healthy range was associated with up to several years of lost lifespan, with most of the absolute mortality burden driven by obesity (BMI ≥30 kg/m^2^). However, the overall association between BMI and mortality was driven by varying associations with individual cause-specific mortality outcomes, including predominantly inverse associations for mental and behavioural, neurological, and external causes. Associations between BMI and mortality varied by age; an improved understanding of the reasons for this interaction could help inform age-specific public health recommendations.
