# Supplementary material for: Association of BMI with overall and cause-specific mortality: a population-based cohort study of 3·6 million adults in the UK
Source: Lancet Diabetes Endocrinol. 2018 Dec;6(12):944–53. doi: 10.1016/S2213-8587(18)30288-2 (PMC6249991; doi:10.1016/S2213-8587(18)30288-2)
Supplement: Supplementary appendix [file mmc1.pdf]

THE LANCET
Diabetes & Endocrinology

Supplementary appendix

This appendix formed part of the original submission and has been peer reviewed. We post it as supplied by the authors.

Supplement to: Bhaskaran K, dos-Santos-Silva I, Leon DA, et al. Association of BMI with overall and cause-specific mortality: a population-based cohort study of 3·6 million adults in the UK. *Lancet Diabetes Endocrinol* 2018; published online Oct 30. http://dx.doi.org/10.1016/S2213-8587(18)30288-2.

Supplementary appendix

Supplement to:

Bhaskaran K, dos Santos Silva I, Leon DA, Douglas IJ, Smeeth L. Body mass index and overall and cause-specific mortality - population-based cohort study among 3.6 million UK adults

Table of contents

	Page
**Part 1 - additional methods**	3
A. Research-quality follow-up in CPRD	3
B. Parametrisation of BMI and covariates	3
C. Calculation of expected age of death	3
**Part 2 – additional results**	4
Table S2.1: Distribution of causes of death	5
Table S2.2: Characteristics of study population by gender and BMI category, restricted to individuals with follow-up available from 5-years post-BMI record	6
Table S2.3: Estimated change points in the BMI-mortality association, and associations with mortality below and above change point, from piecewise 2-line models, among full study population including ever-smokers	8
Table S2.4: Adjusted associations between BMI category and cause-specific mortality, in never-smokers	9
Table S2.5 Adjusted associations between BMI category and cause-specific mortality- full study population	10
Table S2.6: Adjusted associations between BMI in 9 categories and cause-specific mortality, in never-smokers	11
Table S2.7: Adjusted associations between BMI in 9 categories and cause-specific mortality, in total study population	13
Table S2.8: Association between BMI (in 9 categories) and all-cause mortality by ethnicity , among never-smokers	15
Table S2.9: Deaths attributable to suboptimal BMI, and population attributable fractions for all-cause mortality, among never-smokers	16
Table S2.10: Cumulative incidence of the four most common cause-specific mortality outcomes among never-smokers at specific ages, by BMI category	17
Table S2.11: Association between BMI (in 9 categories) and CVD, cancer, respiratory, neurological, mental/behavioural and self-harm/violent mortality among never-smokers, including/excluding those with prevalent disease at start of time at risk	18
Figure S2.1: Participant flow diagram	19
Figure S2.2: Association between BMI and level 2 and 3 cause-specific mortality outcomes among total study population including ever-smokers	20
Figure S2.3: Association between BMI and mortality outcomes, by smoking	21
Figure S2.4: Association between BMI and mortality outcomes among never-smokers, by gender	22
Figure S2.5: Association between BMI and mortality outcomes among never-smokers, by current age	23
Figure S2.6: Association between BMI and mortality outcomes among never-smokers, by deprivation quintile	24

Figure S2.7: Association between BMI and all-cause mortality among never-smokers, by ethnicity	25
Figure S2.8: Cumulative incidence of the four most common cause-specific mortality outcomes among never-smokers, by BMI category	26
Figure S2.9: Association between BMI and all-cause/cause-specific mortality outcomes, with various lengths of excluded follow-up time post-BMI record, in never-smokers	27
Figure S2.10: Association between BMI and CVD, cancer, respiratory, neurological, mental/behavioural and self-harm/violent mortality among never-smokers, excluding or including those with prevalent disease at start of time at risk	28
Figure S2.11: Association between BMI and all-cause mortality in various sensitivity analyses	29
**Part 3 – original study protocol**	31
Protocol: Body mass index and cause-specific morality - population-based cohort study using record linkage	32
Known deviations from approved protocol, and reasons	39
**Part 4 – systematic review of studies investigating links between BMI and cause-specific mortality outcomes –methods and results**	40
Methods for systematic review	41
Table S4.1: Description of studies included in the systematic review	42
Figure S4.1: Flow chart of systematic review search and inclusions/exclusions	50
Figure S4.2: Relative risk estimates for association between BMI and cardiovascular disease mortality from studies in European, North American, Australian and trans-continental settings	51
Figure S4.3: Relative risk estimates of association between BMI and cancers mortality outcomes from studies in European, North American, Australian and trans-continental settings	52
Figure S4.4: Relative risk estimates of association between BMI and mortality outcomes other than CVD/cancer from studies in Europe, North American and trans-continental settings	54
Figure S4.5: Relative risk estimates for association between BMI and cardiovascular disease mortality from studies in Asian settings	55
Figure S4.6: Relative risk estimates of association between BMI and cancers mortality outcomes from studies in Asian settings	56
Figure S4.7: Relative risk estimates of association between BMI and mortality outcomes other than CVD/cancer from studies in Asian settings	57
References for studies included in systematic review	58

Part 1 - Additional Methods

A. Research-standard follow-up in CPRD

CPRD has both patient-level and practice-level data quality indicators. A patient's data is designated as "acceptable" following basic checks including for: valid gender and age, logically consistent registration and transfer-out dates, evidence of valid date recording for clinical and other events. A GP practice is considered up-to-standard if mortality rates are within expected ranges, and there are no gaps in data recording in the practice – the up-to-standard date is then defined the latest date for which all subsequent data in the practice pass these checks. For this study, we included only "acceptable" patients, and we considered research-standard follow-up to start at the latest of the practice up-to-standard date, and the patient's registration date with the practice. Deaths are picked up from linked death data even after the end of active CPRD follow-up, therefore our censoring strategy ignored end of CPRD follow-up and we used administrative censoring at the end of the coverage period for the linked death registry data.

B. Parametrisation of BMI and covariates

In the estimation of life expectancy from age 40 years by BMI, we divided obese into class 1 obese (30-34.9 kg/m^2), class 2 obese (35-39.9), and class 3 obese (≥40), following WHO definitions.

When fitting BMI as a restricted cubic spline, equally spaced knots were used, and the number of knots was determined by fitting models with 3-5 knots and selecting the model which minimised the Akaike Information Criterion.

Age was fitted as a three-knot restricted cubic spline to allow for non-linearity; smoking status was classified as never-, current-, ex-smoker; alcohol use was classified as non-drinker, current drinker [light: 1-2 units/day, moderate: 3-6 units/day, heavy: ≥7 units/day, unknown], ex-drinker; index of multiple deprivation, recorded in twentiles, was fitted as a 3-knot restricted cubic spline to allow for non-linearity; and calendar period was categorised as<1989, 1990-4, 1995-9, 2000-4, 2005-9, ≥2010. All covariates were defined based on the date of the BMI record.

C. Calculation of expected age of death

To estimate expected age of death from age 40, we fitted a simplified Poisson model, restricted to never-smokers and including BMI category, 5-year current age bands, gender, and interactions between BMI and age, and BMI and gender. For each gender, log mortality rates per person year by age band could then be directly predicted. The estimated value of the survival function starting from age 40 at each 5-year age cutoff was then calculated by using these predictions to estimate the cumulative hazard (from age 40) at each age cutoff, taking the negative, and exponentiating. This effectively estimated 5-yearly values of the survival function, conditional on survival to age 40. We could then trivially identify the 5-year interval containing the median of the estimated survival function, and we used linear interpolation within the identified 5-year interval to estimate the exact age of median survival, which we interpreted as expected age of death. We validated this approach by using the same method to estimate expected survival from age 65 by gender (ignoring BMI), for which national statistics were available, and our results were close to those published.

Part 2 - Additional Results

Table S2.1: Distribution of causes of death, overall and by BMI group

Cause of death	Under-weight (%)	Healthy weight (%)	Over-weight (%)	Obese (%)	Total (%)
**All-cause deaths**	9275 (100)	152907 (100)	138432 (100)	66898 (100)	367512 (100)
**Level 1 classification**					
Communicable diseases*	892 (9.6)	11340 (7.4)	9222 (6.7)	4396 (6.6)	25850 (7)
Non-communicable diseases	8018 (86.4)	135685 (88.7)	125583 (90.7)	60968 (91.1)	330254 (89.9)
Injuries/External causes	365 (3.9)	5882 (3.8)	3627 (2.6)	1534 (2.3)	11408 (3.1)
**Level 2 classification (ICD chapter/codes)**					
Cancers (C)	1856 (20)	42835 (28)	41495 (30)	18956 (28.3)	105142 (28.6)
Blood and endocrine (D50-89, E)**	125 (1.3)	1838 (1.2)	2196 (1.6)	1902 (2.8)	6061 (1.6)
Mental and behavioural (F)***	41 (0.4)	531 (0.3)	228 (0.2)	94 (0.1)	894 (0.2)
Neurological (G)	998 (10.8)	15354 (10)	11848 (8.6)	4388 (6.6)	32588 (8.9)
Cardiovascular (I)	2249 (24.2)	45381 (29.7)	46568 (33.6)	23742 (35.5)	117940 (32.1)
Respiratory (J23-99)	1561 (16.8)	13492 (8.8)	9327 (6.7)	4375 (6.5)	28755 (7.8)
Liver cirrhosis (K70.3/71.7/74.3-6)	50 (0.5)	814 (0.5)	697 (0.5)	496 (0.7)	2057 (0.6)
Digestive (K, ex cirrhosis)	436 (4.7)	6404 (4.2)	5687 (4.1)	3195 (4.8)	15722 (4.3)
Musculoskeletal (M)	128 (1.4)	1241 (0.8)	941 (0.7)	517 (0.8)	2827 (0.8)
Urologenital (N)**	152 (1.6)	2686 (1.8)	2734 (2)	1536 (2.3)	7108 (1.9)
Accidental – transport related (V)	26 (0.3)	563 (0.4)	359 (0.3)	160 (0.2)	1108 (0.3)
Accidental ex transport (W/X00-59)	237 (2.6)	3466 (2.3)	2285 (1.7)	1000 (1.5)	6988 (1.9)
Self-harm/ violence (X60-Y09)	66 (0.7)	1318 (0.9)	716 (0.5)	255 (0.4)	2355 (0.6)
Other****	1350 (14.6)	16984 (11.1)	13351 (9.6)	6282 (9.4)	37967 (10.3)
**Level 3 classification (ICD codes)**					
Oesophageal cancer (C15)	99 (1.1)	1909 (1.2)	2078 (1.5)	923 (1.4)	5009 (1.4)
Stomach cancer (C16)	60 (0.6)	1254 (0.8)	1399 (1)	622 (0.9)	3335 (0.9)
Colorectal cancer (C18-21)	147 (1.6)	4117 (2.7)	4423 (3.2)	2047 (3.1)	10734 (2.9)
Liver cancer (C22)	20 (0.2)	741 (0.5)	974 (0.7)	651 (01)	2386 (0.6)
Pancriatic cancer (C25)	84 (0.9)	2138 (1.4)	2269 (1.6)	1101 (1.6)	5592 (1.5)
Lung cancer (C34)	564 (6.1)	10031 (6.6)	8062 (5.8)	3134 (4.7)	21791 (5.9)
Malignant melanoma (C43)	19 (0.2)	631 (0.4)	617 (0.4)	255 (0.4)	1522 (0.4)
Female breast cancer (C50)	162 (1.7)	3630 (2.4)	2823 (2)	1769 (2.6)	8384 (2.3)
Uterus (C54-55)	22 (0.2)	354 (0.2)	399 (0.3)	445 (0.7)	1220 (0.3)
Ovarian cancer (C56)	56 (0.6)	1264 (0.8)	1039 (0.8)	633 (0.9)	2992 (0.8)
Prostate cancer (C61)	47 (0.5)	2701 (1.8)	3494 (2.5)	1003 (1.5)	7245 (2)
Kidney cancer (C64)	34 (0.4)	851 (0.6)	1016 (0.7)	598 (0.9)	2499 (0.7)
Bladder cancer (C67)	49 (0.5)	1354 (0.9)	1441 (1)	588 (0.9)	3432 (0.9)
Brain/CNS cancer (C71-72)	36 (0.4)	1074 (0.7)	1055 (0.8)	428 (0.6)	2593 (0.7)
Haematological malignancy (C81-96)	77 (0.8)	3354 (2.2)	3445 (2.5)	1521 (2.3)	8397 (2.3)
Diabetes mellitus (E10-14)	53 (0.6)	1102 (0.7)	1545 (1.1)	1425 (2.1)	4125 (1.1)
Dementia/Alzheim (F00/01/03, G30)	783 (8.4)	11677 (7.6)	8764 (6.3)	3279 (4.9)	24503 (6.7)
Hypertensive heart dis (I11)	25 (0.3)	554 (0.4)	792 (0.6)	587 (0.9)	1958 (0.5)
Ischaemic heart dis(I20-25)	864 (9.3)	19980 (13.1)	22456 (16.2)	11808 (17.7)	55108 (15)
Atrial fibrillation/flutter (I48)	62 (0.7)	1160 (0.8)	1100 (0.8)	542 (0.8)	2864 (0.8)
Heart failure (I50)	103 (1.1)	1862 (1.2)	1911 (1.4)	1142 (1.7)	5018 (1.4)
Cerebrovascular (I60-69)	736 (7.9)	13431 (8.8)	11738 (8.5)	5075 (7.6)	30980 (8.4)
Aortic dissection (I71.0)	21 (0.2)	536 (0.4)	452 (0.3)	200 (0.3)	1209 (0.3)
Aortic aneurysm (I71.1-9)	71 (0.8)	1545 (1)	1921 (1.4)	702 (1)	4239 (1.2)
Peripheral vascular disease (I73)	49 (0.5)	727 (0.5)	554 (0.4)	250 (0.4)	1580 (0.4)
Lower respiratory infection (J09-22)	766 (8.3)	9656 (6.3)	7764 (5.6)	3599 (5.4)	21785 (5.9)
Falls (W00-19)	70 (0.8)	1213 (0.8)	946 (0.7)	379 (0.6)	2608 (0.7)
Suicide (X60-84)	66 (0.7)	1303 (0.9)	709 (0.5)	249 (0.4)	2327 (0.6)
Other	4130 (44.5)	52758 (34.5)	43246 (31.2)	21943 (32.8)	122077 (33.2)

*The most common communicable diseases deaths had underlying cause recorded as pneumonia (n=20009), unspecified lower respiratory infection (n=1309), septicaemia (n=1617) and bacterial intestinal infections (n=1226); total for these causes = 24161/25850 (93% of communicable diseases deaths); 171 deaths were tuberculosis. Of the 21318 individuals with deaths recorded as pneumonia or unspecified lower respiratory infection, 7541 had no other contributing causes listed, 8759 had a circulatory cause listed as contributory, and 2663 had "senility" listed as contributing. **We split the Global Burden of Diseases "urogenital blood and endocrine" outcome into two as it was felt that the combination was difficult to interpret clinically. ***Including alcohol-related disorders (n=373), disorders related to other psychoactive drugs (n-257), inorganic psychoses (n=96), and mood disorders (n=92); we retained the coding used in the death certificate data and did not attempt to recode any of these deaths (e.g. as external causes). ****Including communicable disease (25,850) and external causes (11,408).

Table S2.2: Characteristics of study population by gender and BMI category, restricted to individuals with follow-up available from 5-years post-BMI record

	Men				***Women***			
**Characteristics** *(cell contents are % except where otherwise state)*	**Underweight**	**Healthy weight**	**Overweight**	**Obese**	**Underweight**	**Healthy weight**	**Overweight**	**Obese**
**N**	33,332	725,879	632,463	252,602	78,745	1,068,110	518,896	322,647
**Person-years from BMI date to end follow-up**								
Mean (sd)	12.5 (5.4)	13.4 (5.7)	12.8 (5.4)	11.5 (5.0)	12.0 (5.3)	12.9 (5.6)	12.4 (5.4)	11.5 (5.1)
Median	11.3	12.5	11.8	10.4	10.8	11.7	11.3	10.3
IQR	(8.0 to 16.1)	(8.7 to 17.7)	(8.3 to 16.4)	(7.5 to 14.3)	(7.7 to 15.4)	(8.2 to 16.8)	(8.0 to 15.8)	(7.4 to 14.4)
Total included follow-up (/1000 p-yrs)	0.232	5.693	4.628	1.558	0.525	7.921	3.620	1.999
**Age (yrs)**								
Median (IQR)	22.8 (18.3 to 32.8)	33.1 (24.7 to 47.7)	42.2 (31.6 to 55.8)	43.7 (33.2 to 55.5)	25.5 (19.8 to 36.5)	32.4 (24.1 to 46.6)	42.2 (29.0 to 58.2)	42.9 (30.2 to 57.3)
**Smoking Status**								
Non-smoker	44.6	48.8	49.5	46.6	56.5	59.5	58.7	57.4
Current smoker	45.4	36.9	29.1	27.7	33.2	27.3	24.8	24.1
Ex-smoker	8.0	13.3	20.8	25.0	9.1	12.6	16.0	18.0
Missing	2.1	0.9	0.6	0.7	1.2	0.6	0.5	0.6
**Alcohol use**								
Non-drinker	22.1	12.6	10.4	11.6	25.0	17.4	19.9	23.8
Current drinker -light (1-2 units/day)	43.5	53.9	55.1	52.4	53.3	64.9	62.8	57.4
Current drinker -moderate (3-6/day)	9.5	17.4	20.1	18.2	2.7	4.1	4.1	3.1
Current drinker -heavy (≥7/day)	3.3	2.9	2.9	3.7	1.0	0.7	0.7	0.7
Current drinker -unknown level	5.2	4.6	4.4	4.8	4.5	4.1	3.9	4.2
Ex-drinker	2.2	1.8	1.9	2.8	2.1	1.8	2.4	3.2

Missing	14.2	6.8	5.2	6.5	11.4	7.1	6.3	7.5
**Any previous diabetes diagnosis**	1.2	2.0	3.9	8.0	0.6	1.0	2.8	6.0
**Index of multiple deprivation quintile**								
1 (low)	15.9	22.6	23.7	19.2	20.9	24.8	21.4	16.8
2	17.4	21.0	22.5	20.9	20.4	22.5	21.9	19.5
3	20.2	20.5	20.9	21.2	20.7	21.1	21.2	20.9
4	22.2	19.2	18.2	20.3	19.7	17.8	19.3	21.6
5 (high)	24.0	16.6	14.6	18.2	18.2	13.8	16.2	21.1
**Ethnicity**								
White	31.3	33.1	36.8	41.3	32.9	35.6	37.7	40.5
South Asian	5.6	3.3	2.6	1.9	4.5	2.3	2.3	1.9
Black	1.7	1.6	1.6	1.8	1.2	1.2	2.0	3.0
Other	1.5	1.2	0.9	0.8	2.2	1.2	0.8	0.7
Mixed	0.5	0.5	0.4	0.4	0.6	0.5	0.4	0.5
Missing	59.3	60.4	57.7	53.9	58.7	59.3	56.7	53.4
**Calendar year**								
<1989	0.7	1.0	1.0	0.7	0.7	1.0	1.0	0.8
1990-1994	14.4	21.8	20.1	13.3	14.9	20.7	20.5	15.5
1995-1999	19.2	21.8	21.3	18.1	20.1	22.7	22.6	20.4
2000-2004	25.0	23.1	24.4	27.2	25.3	23.3	24.1	26.4
2005-2009	33.4	26.8	27.6	33.3	31.8	26.7	26.4	30.2
≥2010	7.3	5.5	5.6	7.4	7.1	5.6	5.4	6.8

Note: Characteristics are at time of first BMI record used in study where applicable; smoking was assigned using record from same date as BMI record or within 1 year before where available (84% of patients), else using the nearest record in year after BMI (3%), else using the nearest record >1 year before BMI (8%), else using the nearest record >1 year after BMI (4%); a similar algorithm was used for alcohol; for ethnicity, earliest available record was used.

Table S2.3: Estimated change points in the BMI-mortality association, and associations with mortality below and above change point, from piecewise 2-line models, among full study population including ever-smokers

Outcome	BMI change point (kg/m^2)	HR per $5kg/m^2$ BMI increase below change point*	HR per $5kg/m^2$ BMI increase above change point
**All-cause mortality**	25 (25-25)	0.74 (0.73-0.74)	1.18 (1.17-1.18)
**Level 1 outcomes**			
Communicable diseases	26 (26-26)	0.68 (0.66-0.70)	1.27 (1.24-1.30)
Non-communicable diseases	25 (25-25)	0.75 (0.74-0.76)	1.18 (1.17-1.19)
Injuries/External causes	28 (27-28)	0.73 (0.70-0.76)	1.12 (1.07-1.17)
**Level 2 outcomes (ICD chapters/codes)**			
Cancers (C)	24 (24-25)	0.86 (0.84-0.88)	1.09 (1.08-1.10)
Blood and endocrine (D50-89, E)	28 (28-29)	0.92 (0.87-0.97)	1.59 (1.53-1.65)
Mental and behavioural (F)	26 (25-27)	0.52 (0.45-0.60)	1.05 (0.92-1.21)
Neurological (G)	28 (27-29)	0.72 (0.71-0.74)	1.02 (0.99-1.05)
Cardiovascular (I)	25 (25-25)	0.87 (0.85-0.88)	1.26 (1.25-1.27)
Respiratory (J23-99)	25 (25-25)	0.40 (0.39-0.42)	1.17 (1.15-1.20)
Liver cirrhosis (K70.3/71.7/74.3-6)	25 (23-26)	0.71 (0.62-0.81)	1.30 (1.23-1.38)
Digestive (K, ex cirrhosis)	25 (25-25)	0.69 (0.66-0.72)	1.28 (1.25-1.31)
Musculoskeletal (M)	25 (24-25)	0.52 (0.47-0.58)	1.27 (1.20-1.34)
Urogenital (N)	25 (25-26)	0.83 (0.77-0.89)	1.39 (1.34-1.43)
Accidental transport-related (V)	N/A*	0.96 (0.89-1.04)	
Accidental ex transport (W/X00-59)	28 (27-28)	0.72 (0.68-0.75)	1.19 (1.12-1.25)
Self-harm/interpersonal violence (X60-Y09)	28 (25-30)	0.72 (0.67-0.78)	0.95 (0.85-1.06)

*For transport-related accidents there was little or no evidence against linearity (Fig S2.2) so a single linear effect without change point was estimated.

Table S2.4: Adjusted associations between BMI category and cause-specific mortality, in never-smokers

	HR compared with healthy weight			
**Mortality Outcome**	Underweight (<18.5kg/m²)	Healthy weight (18.5-25 kg/m²)	Overweight (25-30 kg/m²)	Obese (>30 kg/m²)
**All-causes**	1.36 (1.32-1.41)	1.00 (REF)	1.00 (0.99-1.01)	1.25 (1.23-1.26)
**High-level classification**				
Communicable Diseases	1.46 (1.32-1.61)	1.00 (REF)	0.90 (0.87-0.93)	1.14 (1.08-1.19)
Non-communicable Diseases	1.36 (1.31-1.40)	1.00 (REF)	1.01 (1.00-1.02)	1.27 (1.25-1.29)
External causes	1.28 (1.07-1.53)	1.00 (REF)	0.83 (0.78-0.89)	0.89 (0.82-0.97)
**Mid-level classification**				
Cancers	1.05 (0.97-1.15)	1.00 (REF)	1.11 (1.09-1.14)	1.30 (1.27-1.34)
Blood/endocrine	1.72 (1.35-2.19)	1.00 (REF)	1.08 (0.99-1.17)	1.86 (1.70-2.04)
Mental health	2.92 (1.72-4.95)	1.00 (REF)	0.65 (0.48-0.86)	0.85 (0.59-1.21)
Neurological	1.58 (1.45-1.71)	1.00 (REF)	0.79 (0.77-0.82)	0.75 (0.71-0.78)
Cardiovascular	1.16 (1.09-1.23)	1.00 (REF)	1.06 (1.04-1.08)	1.42 (1.39-1.45)
Respiratory	2.30 (2.06-2.57)	1.00 (REF)	0.84 (0.80-0.88)	1.06 (1.00-1.13)
Cirrhosis	1.01 (0.45-2.28)	1.00 (REF)	1.11 (0.92-1.33)	2.12 (1.75-2.57)
Digestive (ex cirrhosis)	1.58 (1.35-1.85)	1.00 (REF)	1.09 (1.04-1.15)	1.58 (1.48-1.68)
Musculoskeletal	2.20 (1.72-2.81)	1.00 (REF)	0.80 (0.71-0.89)	1.11 (0.97-1.27)
Urogenital	1.49 (1.21-1.83)	1.00 (REF)	1.13 (1.05-1.22)	1.74 (1.59-1.89)
Accident– transport related	0.98 (0.52-1.85)	1.00 (REF)	0.93 (0.76-1.13)	1.10 (0.85-1.42)
Accident – non-transport	1.41 (1.15-1.74)	1.00 (REF)	0.79 (0.73-0.86)	0.89 (0.80-0.99)
Self-harm/violence	0.97 (0.58-1.60)	1.00 (REF)	0.96 (0.83-1.10)	0.78 (0.64-0.97)
**Specific Cancers**				
Oesophageal	1.88 (1.36-2.62)	1.00 (REF)	1.13 (1.03-1.25)	1.44 (1.28-1.62)
Gastric	1.08 (0.68-1.74)	1.00 (REF)	1.24 (1.11-1.39)	1.40 (1.21-1.61)
Colorectal	0.97 (0.76-1.24)	1.00 (REF)	1.12 (1.05-1.18)	1.32 (1.23-1.42)
Liver	1.18 (0.63-2.21)	1.00 (REF)	1.37 (1.19-1.59)	2.01 (1.71-2.36)
Pancreas	1.30 (0.94-1.80)	1.00 (REF)	1.10 (1.01-1.20)	1.29 (1.16-1.44)
Lung	0.99 (0.73-1.34)	1.00 (REF)	1.00 (0.94-1.08)	1.01 (0.92-1.11)
Melanoma	1.18 (0.66-2.10)	1.00 (REF)	1.00 (0.86-1.17)	1.16 (0.96-1.41)
Breast	0.93 (0.74-1.16)	1.00 (REF)	1.13 (1.06-1.20)	1.32 (1.22-1.42)
Uterus	1.30 (0.71-2.38)	1.00 (REF)	1.33 (1.12-1.59)	2.71 (2.27-3.22)
Ovarian	1.12 (0.77-1.64)	1.00 (REF)	1.18 (1.06-1.31)	1.34 (1.19-1.52)
Prostate	0.88 (0.56-1.39)	1.00 (REF)	1.12 (1.04-1.20)	1.07 (0.96-1.19)
Kidney	1.07 (0.58-1.94)	1.00 (REF)	1.23 (1.08-1.41)	1.90 (1.62-2.21)
Bladder	0.87 (0.52-1.46)	1.00 (REF)	1.13 (1.01-1.26)	1.24 (1.07-1.44)
Brain/CNS	0.82 (0.48-1.40)	1.00 (REF)	1.05 (0.94-1.18)	1.02 (0.88-1.18)
Haematological	0.91 (0.68-1.21)	1.00 (REF)	1.08 (1.01-1.15)	1.18 (1.09-1.29)
**Specific Endocrine**				
Diabetes	1.05 (0.70-1.56)	1.00 (REF)	1.17 (1.05-1.30)	1.95 (1.74-2.18)
**Specific Neurological**				
Dementia/Alzheimer's	1.53 (1.40-1.68)	1.00 (REF)	0.78 (0.75-0.81)	0.75 (0.71-0.79)
**Specific CVD**				
Hypertensive heart disease	1.29 (0.76-2.22)	1.00 (REF)	1.50 (1.28-1.74)	2.64 (2.23-3.12)
Ischaemic heart disease	1.09 (0.98-1.21)	1.00 (REF)	1.11 (1.08-1.14)	1.57 (1.52-1.62)
Atrial flutter/fibrillation	1.04 (0.74-1.46)	1.00 (REF)	1.01 (0.91-1.12)	1.32 (1.16-1.51)
Heart Failure	1.18 (0.91-1.52)	1.00 (REF)	1.13 (1.03-1.23)	1.82 (1.64-2.01)
Cerebrovascular	1.20 (1.09-1.33)	1.00 (REF)	0.96 (0.93-0.99)	1.05 (1.01-1.10)
Aortic dissection	1.20 (0.66-2.20)	1.00 (REF)	0.96 (0.81-1.14)	1.14 (0.91-1.42)
Aortic aneurysm	0.93 (0.55-1.55)	1.00 (REF)	1.29 (1.14-1.44)	1.25 (1.06-1.48)
Peripheral vascular disease	1.17 (0.73-1.86)	1.00 (REF)	0.91 (0.77-1.07)	1.13 (0.92-1.40)
**Specific respiratory**				
Lower respiratory infection	1.47 (1.32-1.63)	1.00 (REF)	0.89 (0.86-0.93)	1.11 (1.05-1.17)
**Specific external**				
Falls	1.68 (1.23-2.31)	1.00 (REF)	0.86 (0.76-0.97)	0.85 (0.72-1.00)
Suicide	0.98 (0.59-1.62)	1.00 (REF)	0.95 (0.82-1.10)	0.77 (0.62-0.95)

Note: 5-year exclusion period after BMI applied in all models; estimates adjusted for age, deprivation, calendar year, diabetes, alcohol status, and stratified for gender

Table S2.5: Adjusted associations between BMI category and cause-specific mortality- full study population

	HR compared with healthy weight			
**Mortality Outcome**	Underweight (<18.5kg/m²)	Healthy weight (18.5-25 kg/m²)	Overweight (25-30 kg/m²)	Obese (>30 kg/m²)
**All-causes**	1.46 (1.43-1.49)	1.00 (REF)	0.94 (0.93-0.95)	1.13 (1.12-1.14)
**High-level classification**				
Communicable Diseases	1.67 (1.56-1.78)	1.00 (REF)	0.87 (0.84-0.89)	1.07 (1.04-1.12)
Non-communicable Diseases	1.45 (1.42-1.48)	1.00 (REF)	0.95 (0.94-0.96)	1.14 (1.13-1.16)
External causes	1.25 (1.12-1.40)	1.00 (REF)	0.77 (0.74-0.80)	0.81 (0.76-0.86)
**Mid-level classification**				
Cancers	1.19 (1.13-1.24)	1.00 (REF)	1.01 (0.99-1.02)	1.12 (1.10-1.14)
Blood/endocrine	1.70 (1.42-2.04)	1.00 (REF)	1.05 (0.98-1.12)	1.74 (1.62-1.87)
Mental health	1.42 (1.03-1.96)	1.00 (REF)	0.56 (0.47-0.66)	0.66 (0.53-0.83)
Neurological	1.52 (1.43-1.63)	1.00 (REF)	0.79 (0.77-0.81)	0.72 (0.69-0.74)
Cardiovascular	1.19 (1.14-1.24)	1.00 (REF)	1.04 (1.03-1.05)	1.36 (1.34-1.38)
Respiratory	2.75 (2.61-2.90)	1.00 (REF)	0.69 (0.67-0.71)	0.81 (0.78-0.84)
Cirrhosis	1.54 (1.16-2.06)	1.00 (REF)	0.91 (0.82-1.01)	1.42 (1.26-1.59)
Digestive (ex cirrhosis)	1.65 (1.50-1.81)	1.00 (REF)	0.94 (0.90-0.97)	1.28 (1.23-1.34)
Musculoskeletal	2.14 (1.77-2.59)	1.00 (REF)	0.80 (0.73-0.88)	1.09 (0.98-1.22)
Urogenital	1.33 (1.14-1.56)	1.00 (REF)	1.06 (1.00-1.12)	1.54 (1.44-1.64)
Accident– transport related	1.01 (0.68-1.52)	1.00 (REF)	0.88 (0.77-1.01)	0.99 (0.83-1.20)
Accident – non-transport	1.37 (1.20-1.57)	1.00 (REF)	0.76 (0.72-0.81)	0.84 (0.78-0.91)
Self-harm/violence	1.05 (0.81-1.37)	1.00 (REF)	0.75 (0.68-0.83)	0.67 (0.59-0.77)
**Specific Cancers**				
Oesophageal	1.70 (1.39-2.08)	1.00 (REF)	1.01 (0.95-1.08)	1.19 (1.10-1.29)
Gastric	1.23 (0.93-1.62)	1.00 (REF)	1.10 (1.02-1.20)	1.22 (1.11-1.35)
Colorectal	1.05 (0.89-1.24)	1.00 (REF)	1.07 (1.02-1.11)	1.24 (1.18-1.32)
Liver	0.91 (0.59-1.41)	1.00 (REF)	1.27 (1.15-1.41)	1.85 (1.66-2.07)
Pancreas	1.11 (0.89-1.39)	1.00 (REF)	1.08 (1.02-1.15)	1.24 (1.15-1.34)
Lung	1.36 (1.25-1.48)	1.00 (REF)	0.84 (0.82-0.87)	0.80 (0.77-0.84)
Melanoma	0.97 (0.61-1.54)	1.00 (REF)	1.05 (0.93-1.18)	1.12 (0.96-1.30)
Breast	0.95 (0.80-1.12)	1.00 (REF)	1.14 (1.08-1.20)	1.30 (1.23-1.38)
Uterus	1.45 (0.92-2.28)	1.00 (REF)	1.38 (1.19-1.60)	2.72 (2.35-3.15)
Ovarian	1.08 (0.82-1.43)	1.00 (REF)	1.10 (1.01-1.20)	1.24 (1.12-1.37)
Prostate	0.74 (0.55-1.01)	1.00 (REF)	1.08 (1.03-1.14)	1.07 (0.99-1.15)
Kidney	1.13 (0.78-1.63)	1.00 (REF)	1.17 (1.06-1.29)	1.67 (1.50-1.87)
Bladder	1.12 (0.84-1.48)	1.00 (REF)	1.02 (0.95-1.11)	1.19 (1.07-1.31)
Brain/CNS	0.92 (0.64-1.34)	1.00 (REF)	1.10 (1.01-1.21)	1.05 (0.93-1.18)
Haematological	0.75 (0.60-0.93)	1.00 (REF)	1.03 (0.98-1.09)	1.13 (1.06-1.21)
**Specific Endocrine**				
Diabetes	1.09 (0.81-1.46)	1.00 (REF)	1.13 (1.04-1.23)	1.79 (1.64-1.95)
**Specific Neurological**				
Dementia/Alzheimer's	1.48 (1.38-1.59)	1.00 (REF)	0.77 (0.75-0.79)	0.72 (0.69-0.75)
**Specific CVD**				
Hypertensive heart disease	1.16 (0.78-1.73)	1.00 (REF)	1.45 (1.29-1.62)	2.76 (2.45-3.12)
Ischaemic heart disease	1.14 (1.06-1.22)	1.00 (REF)	1.10 (1.07-1.12)	1.47 (1.43-1.50)
Atrial flutter/fibrillation	1.14 (0.88-1.46)	1.00 (REF)	1.00 (0.92-1.09)	1.25 (1.13-1.40)
Heart Failure	1.21 (0.99-1.47)	1.00 (REF)	1.12 (1.05-1.20)	1.77 (1.64-1.92)
Cerebrovascular	1.23 (1.14-1.32)	1.00 (REF)	0.92 (0.90-0.95)	1.02 (0.98-1.05)
Aortic dissection	1.11 (0.72-1.71)	1.00 (REF)	0.95 (0.83-1.08)	1.12 (0.94-1.32)
Aortic aneurysm	1.08 (0.85-1.38)	1.00 (REF)	1.23 (1.14-1.32)	1.33 (1.21-1.46)
Peripheral vascular disease	1.38 (1.04-1.84)	1.00 (REF)	0.79 (0.70-0.89)	0.95 (0.82-1.11)
**Specific respiratory**				
Lower respiratory infection	1.65 (1.53-1.78)	1.00 (REF)	0.86 (0.84-0.89)	1.06 (1.02-1.10)
**Specific external**				
Falls	1.34 (1.05-1.71)	1.00 (REF)	0.80 (0.73-0.87)	0.81 (0.72-0.91)
Suicide	1.05 (0.80-1.37)	1.00 (REF)	0.75 (0.68-0.82)	0.66 (0.58-0.76)

Note: 5-year exclusion period after BMI applied in all models; estimates adjusted for age, deprivation, calendar year, diabetes, alcohol status, smoking (all as defined at date of BMI measure), and stratified for gender

Table S2.6: Adjusted associations between BMI in 9 categories and cause-specific mortality, in never-smokers

	**Hazard ratio**								
BMI category (kg/m2)	*<18.5*	*18.5-19.9*	*20.0-22.4*	*22.5-24.9*	*25.0-27.4*	*27.5-29.9*	*30.0-34.9*	*35.0-39.9*	*≥40.0*
**Mortality Outcome**									
**All-causes**	1.42 (1.37-1.47)	1.20 (1.17-1.23)	1.07 (1.05-1.08)	1.00 (REF)	1.01 (1.00-1.03)	1.07 (1.05-1.08)	1.20 (1.19-1.22)	1.48 (1.44-1.52)	2.10 (2.02-2.18)
**High-level classification**									
Communicable Diseases	1.58 (1.43-1.75)	1.36 (1.25-1.47)	1.14 (1.08-1.20)	1.00 (REF)	0.96 (0.91-1.01)	0.98 (0.93-1.04)	1.12 (1.05-1.19)	1.50 (1.36-1.65)	2.23 (1.92-2.60)
Non-communicable Diseases	1.40 (1.35-1.45)	1.17 (1.14-1.21)	1.06 (1.04-1.07)	1.00 (REF)	1.02 (1.01-1.04)	1.08 (1.06-1.10)	1.22 (1.20-1.24)	1.49 (1.45-1.53)	2.12 (2.04-2.21)
External causes	1.42 (1.18-1.70)	1.38 (1.20-1.58)	1.19 (1.09-1.29)	1.00 (REF)	0.93 (0.85-1.00)	0.88 (0.80-0.97)	0.93 (0.84-1.03)	1.06 (0.89-1.26)	1.15 (0.87-1.54)
**Mid-level classification**									
Cancers	1.05 (0.96-1.14)	0.97 (0.92-1.03)	0.99 (0.96-1.02)	1.00 (REF)	1.08 (1.05-1.11)	1.15 (1.12-1.19)	1.25 (1.21-1.29)	1.38 (1.32-1.46)	1.60 (1.48-1.73)
Blood/endocrine	1.72 (1.34-2.20)	1.18 (0.94-1.47)	0.96 (0.83-1.10)	1.00 (REF)	1.09 (0.98-1.22)	1.05 (0.93-1.19)	1.53 (1.36-1.72)	2.39 (2.05-2.78)	4.48 (3.73-5.38)
Mental health	3.97 (2.25-7.01)	2.44 (1.45-4.10)	1.60 (1.09-2.33)	1.00 (REF)	0.80 (0.53-1.19)	0.92 (0.59-1.43)	1.27 (0.83-1.94)	0.83 (0.36-1.95)	0.00 (0.00-0.00)
Neurological	1.75 (1.61-1.90)	1.43 (1.33-1.53)	1.19 (1.14-1.25)	1.00 (REF)	0.89 (0.85-0.93)	0.83 (0.79-0.87)	0.82 (0.77-0.86)	0.78 (0.70-0.86)	1.04 (0.89-1.23)
Cardiovascular	1.18 (1.11-1.26)	1.11 (1.06-1.16)	1.03 (1.00-1.06)	1.00 (REF)	1.04 (1.02-1.07)	1.14 (1.11-1.17)	1.32 (1.28-1.35)	1.75 (1.67-1.82)	2.68 (2.51-2.86)
Respiratory	2.60 (2.32-2.92)	1.73 (1.56-1.91)	1.21 (1.13-1.29)	1.00 (REF)	0.92 (0.87-0.98)	0.96 (0.89-1.03)	1.09 (1.02-1.18)	1.36 (1.21-1.53)	2.28 (1.91-2.71)
Cirrhosis	1.03 (0.45-2.34)	0.85 (0.48-1.52)	1.08 (0.82-1.43)	1.00 (REF)	0.99 (0.78-1.26)	1.33 (1.04-1.71)	1.95 (1.54-2.46)	2.69 (1.97-3.68)	3.10 (2.00-4.80)
Digestive (ex cirrhosis)	1.59 (1.35-1.86)	1.01 (0.88-1.17)	1.01 (0.93-1.09)	1.00 (REF)	1.07 (0.99-1.14)	1.15 (1.06-1.24)	1.43 (1.32-1.55)	1.87 (1.66-2.10)	2.97 (2.51-3.50)
Musculoskeletal	2.54 (1.96-3.28)	1.69 (1.34-2.13)	1.26 (1.08-1.47)	1.00 (REF)	0.93 (0.81-1.08)	0.88 (0.74-1.05)	1.13 (0.95-1.34)	1.58 (1.22-2.04)	2.42 (1.67-3.50)
Urogenital	1.48 (1.20-1.83)	1.13 (0.95-1.35)	0.95 (0.85-1.07)	1.00 (REF)	1.05 (0.96-1.16)	1.25 (1.13-1.38)	1.52 (1.37-1.69)	2.26 (1.93-2.64)	3.67 (2.93-4.61)
Accident– transport related	1.05 (0.54-2.01)	1.15 (0.74-1.78)	1.13 (0.86-1.47)	1.00 (REF)	0.98 (0.77-1.26)	0.96 (0.72-1.29)	1.23 (0.92-1.66)	0.62 (0.30-1.27)	1.90 (0.93-3.91)
Accident – non-transport	1.54 (1.25-1.91)	1.35 (1.14-1.60)	1.17 (1.05-1.30)	1.00 (REF)	0.85 (0.77-0.94)	0.87 (0.77-0.98)	0.89 (0.78-1.01)	1.20 (0.97-1.47)	1.33 (0.94-1.89)
Self-harm/violence	1.10 (0.66-1.84)	1.45 (1.07-1.98)	1.22 (1.00-1.49)	1.00 (REF)	1.15 (0.96-1.38)	0.91 (0.72-1.14)	0.93 (0.73-1.18)	0.68 (0.42-1.10)	0.73 (0.34-1.56)
**Specific Cancers**									
Oesophageal	1.90 (1.36-2.65)	1.38 (1.06-1.80)	0.94 (0.80-1.10)	1.00 (REF)	1.02 (0.90-1.16)	1.32 (1.16-1.51)	1.42 (1.23-1.64)	1.44 (1.13-1.85)	1.99 (1.36-2.91)
Gastric	1.17 (0.73-1.89)	1.33 (0.97-1.82)	1.16 (0.97-1.39)	1.00 (REF)	1.27 (1.10-1.47)	1.43 (1.22-1.68)	1.42 (1.20-1.69)	1.71 (1.30-2.26)	2.01 (1.29-3.14)
Colorectal	0.95 (0.74-1.22)	0.98 (0.83-1.16)	0.95 (0.86-1.04)	1.00 (REF)	1.06 (0.99-1.15)	1.15 (1.06-1.25)	1.26 (1.16-1.38)	1.39 (1.20-1.60)	1.51 (1.19-1.91)
Liver	1.08 (0.57-2.04)	1.02 (0.66-1.59)	0.74 (0.57-0.95)	1.00 (REF)	1.13 (0.94-1.35)	1.48 (1.22-1.78)	1.66 (1.37-2.02)	2.34 (1.79-3.07)	2.80 (1.88-4.18)
Pancreas	1.30 (0.94-1.81)	0.89 (0.68-1.16)	1.03 (0.91-1.18)	1.00 (REF)	1.11 (1.00-1.24)	1.09 (0.96-1.24)	1.27 (1.12-1.44)	1.52 (1.25-1.85)	0.97 (0.65-1.45)
Lung	0.99 (0.73-1.34)	1.19 (0.98-1.44)	0.97 (0.87-1.08)	1.00 (REF)	1.01 (0.92-1.10)	1.00 (0.91-1.11)	1.04 (0.93-1.15)	1.03 (0.85-1.26)	0.58 (0.37-0.91)
Melanoma	1.16 (0.65-2.09)	0.82 (0.52-1.29)	1.00 (0.80-1.26)	1.00 (REF)	1.04 (0.86-1.26)	0.91 (0.72-1.14)	1.19 (0.95-1.50)	0.80 (0.50-1.26)	1.57 (0.89-2.77)

Breast	0.91 (0.73-1.14)	0.92 (0.79-1.07)	0.98 (0.90-1.08)	1.00 (REF)	1.05 (0.97-1.15)	1.21 (1.10-1.33)	1.31 (1.19-1.43)	1.27 (1.10-1.46)	1.36 (1.10-1.67)
Uterus	1.22 (0.66-2.25)	0.57 (0.32-1.03)	0.94 (0.72-1.23)	1.00 (REF)	1.11 (0.88-1.41)	1.48 (1.16-1.89)	2.08 (1.66-2.61)	2.95 (2.23-3.91)	5.89 (4.30-8.06)
Ovarian	1.13 (0.77-1.65)	0.74 (0.55-1.01)	1.07 (0.92-1.25)	1.00 (REF)	1.17 (1.02-1.35)	1.20 (1.03-1.41)	1.26 (1.08-1.48)	1.45 (1.16-1.81)	1.86 (1.37-2.51)
Prostate	0.85 (0.54-1.35)	1.01 (0.77-1.32)	0.89 (0.79-1.01)	1.00 (REF)	1.06 (0.97-1.16)	1.14 (1.03-1.26)	1.06 (0.94-1.19)	0.96 (0.72-1.26)	0.68 (0.34-1.36)
Kidney	1.11 (0.61-2.04)	0.68 (0.41-1.14)	1.19 (0.96-1.47)	1.00 (REF)	1.17 (0.98-1.40)	1.45 (1.20-1.74)	1.77 (1.46-2.14)	2.31 (1.74-3.07)	3.91 (2.70-5.67)
Bladder	0.91 (0.54-1.52)	1.05 (0.75-1.47)	1.11 (0.93-1.33)	1.00 (REF)	1.08 (0.93-1.25)	1.31 (1.12-1.54)	1.29 (1.08-1.54)	1.23 (0.88-1.72)	1.43 (0.80-2.55)
Brain/CNS	0.81 (0.48-1.39)	0.92 (0.66-1.27)	0.99 (0.83-1.18)	1.00 (REF)	1.06 (0.92-1.23)	1.01 (0.86-1.20)	1.01 (0.84-1.21)	1.07 (0.79-1.44)	0.84 (0.49-1.43)
Haematological	0.93 (0.69-1.24)	1.01 (0.84-1.23)	1.06 (0.96-1.18)	1.00 (REF)	1.10 (1.01-1.19)	1.10 (1.00-1.21)	1.19 (1.08-1.32)	1.18 (0.99-1.40)	1.51 (1.16-1.97)
**Specific Endocrine**									
Diabetes	1.01 (0.68-1.52)	1.01 (0.74-1.38)	0.91 (0.76-1.09)	1.00 (REF)	1.18 (1.03-1.35)	1.09 (0.93-1.26)	1.58 (1.38-1.82)	2.60 (2.18-3.11)	3.58 (2.83-4.53)
**Specific Neurological**									
Dementia/Alzheimer’s	1.70 (1.55-1.87)	1.40 (1.29-1.51)	1.20 (1.14-1.26)	1.00 (REF)	0.88 (0.84-0.92)	0.82 (0.78-0.87)	0.83 (0.78-0.88)	0.75 (0.67-0.84)	1.11 (0.92-1.34)
**Specific CVD**									
Hypertensive heart disease	1.30 (0.75-2.24)	0.88 (0.54-1.41)	1.03 (0.80-1.33)	1.00 (REF)	1.39 (1.14-1.69)	1.67 (1.35-2.06)	2.08 (1.68-2.58)	3.56 (2.68-4.73)	9.02 (6.51-12.51)
Ischaemic heart disease	1.10 (0.99-1.22)	1.06 (0.98-1.14)	1.01 (0.96-1.05)	1.00 (REF)	1.05 (1.02-1.09)	1.23 (1.18-1.28)	1.44 (1.39-1.50)	1.96 (1.84-2.09)	2.73 (2.48-3.01)
Atrial flutter/fibrillation	1.08 (0.77-1.53)	1.12 (0.87-1.45)	1.08 (0.92-1.27)	1.00 (REF)	1.06 (0.92-1.22)	1.02 (0.87-1.20)	1.24 (1.06-1.46)	1.75 (1.35-2.26)	2.43 (1.59-3.71)
Heart Failure	1.16 (0.89-1.50)	0.95 (0.76-1.18)	0.97 (0.85-1.10)	1.00 (REF)	1.04 (0.93-1.16)	1.23 (1.09-1.39)	1.54 (1.36-1.74)	2.30 (1.90-2.78)	5.29 (4.12-6.77)
Cerebrovascular	1.26 (1.13-1.39)	1.23 (1.14-1.33)	1.06 (1.01-1.12)	1.00 (REF)	1.00 (0.95-1.04)	0.99 (0.94-1.04)	1.04 (0.99-1.10)	1.19 (1.09-1.30)	1.64 (1.42-1.90)
Aortic dissection	1.22 (0.66-2.26)	1.21 (0.79-1.87)	0.99 (0.77-1.29)	1.00 (REF)	1.04 (0.83-1.29)	0.86 (0.66-1.13)	1.20 (0.92-1.56)	0.96 (0.57-1.60)	1.08 (0.44-2.65)
Aortic aneurysm	0.86 (0.51-1.45)	0.80 (0.55-1.17)	0.84 (0.69-1.02)	1.00 (REF)	1.20 (1.04-1.38)	1.22 (1.04-1.44)	1.17 (0.97-1.41)	1.22 (0.85-1.76)	1.15 (0.54-2.43)
Peripheral vascular disease	1.26 (0.78-2.02)	1.28 (0.89-1.85)	1.14 (0.90-1.44)	1.00 (REF)	1.01 (0.81-1.24)	0.91 (0.71-1.17)	1.18 (0.92-1.52)	1.41 (0.91-2.19)	1.08 (0.40-2.92)
**Specific respiratory**									
Lower respiratory infection	1.59 (1.43-1.77)	1.37 (1.25-1.50)	1.14 (1.08-1.21)	1.00 (REF)	0.95 (0.90-1.00)	0.98 (0.92-1.04)	1.08 (1.02-1.16)	1.46 (1.31-1.62)	2.34 (1.99-2.75)
**Specific external**									
Falls	1.72 (1.25-2.38)	1.11 (0.83-1.49)	1.03 (0.87-1.23)	1.00 (REF)	0.89 (0.77-1.03)	0.85 (0.72-1.01)	0.81 (0.67-0.98)	1.03 (0.74-1.43)	1.20 (0.69-2.10)
Suicide	1.12 (0.67-1.88)	1.49 (1.09-2.03)	1.23 (1.01-1.50)	1.00 (REF)	1.14 (0.95-1.37)	0.91 (0.73-1.15)	0.92 (0.72-1.17)	0.65 (0.39-1.06)	0.74 (0.35-1.58)

Note: 5-year exclusion period after BMI applied in all models; estimates adjusted for age, deprivation, calendar year, diabetes, alcohol status, and stratified for gender

Table S2.7: Adjusted associations between BMI in 9 categories and cause-specific mortality, in total study population

	Hazard ratio								
BMI category (kg/m2)	*<18.5*	*18.5-19.9*	*20.0-22.4*	*22.5-24.9*	*25.0-27.4*	*27.5-29.9*	*30.0-34.9*	*35.0-39.9*	*≥40.0*
**Mortality Outcome**									
**All-causes**	1.56 (1.52-1.59)	1.29 (1.27-1.32)	1.11 (1.10-1.12)	1.00 (REF)	0.98 (0.97-0.99)	1.01 (1.00-1.03)	1.12 (1.10-1.13)	1.36 (1.33-1.38)	1.88 (1.83-1.93)
**High-level classification**									
Communicable Diseases	1.84 (1.72-1.98)	1.44 (1.35-1.53)	1.18 (1.13-1.23)	1.00 (REF)	0.94 (0.90-0.98)	0.97 (0.93-1.01)	1.07 (1.02-1.12)	1.42 (1.32-1.53)	2.26 (2.02-2.52)
Non-communicable Diseases	1.54 (1.50-1.57)	1.27 (1.25-1.30)	1.10 (1.09-1.11)	1.00 (REF)	0.99 (0.98-1.00)	1.03 (1.01-1.04)	1.13 (1.11-1.14)	1.37 (1.34-1.40)	1.89 (1.83-1.94)
External causes	1.43 (1.27-1.60)	1.45 (1.33-1.58)	1.22 (1.15-1.29)	1.00 (REF)	0.88 (0.84-0.94)	0.82 (0.76-0.87)	0.88 (0.82-0.95)	0.93 (0.82-1.05)	1.09 (0.90-1.32)
**Mid-level classification**									
Cancers	1.22 (1.16-1.28)	1.13 (1.09-1.17)	1.04 (1.02-1.06)	1.00 (REF)	1.01 (0.99-1.03)	1.06 (1.04-1.08)	1.11 (1.08-1.13)	1.24 (1.19-1.28)	1.38 (1.30-1.45)
Blood/endocrine	1.78 (1.48-2.14)	1.24 (1.05-1.47)	1.07 (0.97-1.19)	1.00 (REF)	1.12 (1.03-1.22)	1.06 (0.97-1.17)	1.47 (1.35-1.61)	2.36 (2.10-2.64)	4.29 (3.74-4.92)
Mental health	1.73 (1.23-2.42)	1.62 (1.24-2.11)	1.34 (1.10-1.62)	1.00 (REF)	0.63 (0.50-0.79)	0.72 (0.56-0.93)	0.79 (0.60-1.02)	0.90 (0.59-1.40)	0.47 (0.17-1.26)
Neurological	1.69 (1.58-1.81)	1.42 (1.34-1.50)	1.20 (1.16-1.24)	1.00 (REF)	0.90 (0.87-0.93)	0.81 (0.78-0.85)	0.79 (0.75-0.82)	0.76 (0.70-0.82)	0.99 (0.87-1.13)
Cardiovascular	1.22 (1.17-1.28)	1.13 (1.09-1.17)	1.04 (1.02-1.06)	1.00 (REF)	1.03 (1.01-1.05)	1.12 (1.09-1.14)	1.27 (1.25-1.30)	1.65 (1.60-1.70)	2.49 (2.37-2.61)
Respiratory	3.40 (3.21-3.59)	2.13 (2.02-2.25)	1.38 (1.33-1.44)	1.00 (REF)	0.84 (0.81-0.87)	0.83 (0.80-0.87)	0.90 (0.86-0.94)	1.12 (1.04-1.21)	1.79 (1.61-1.99)
Cirrhosis	1.71 (1.27-2.30)	1.21 (0.95-1.55)	1.23 (1.06-1.42)	1.00 (REF)	0.95 (0.82-1.09)	1.06 (0.91-1.24)	1.43 (1.23-1.65)	1.84 (1.50-2.27)	2.15 (1.59-2.90)
Digestive (ex cirrhosis)	1.79 (1.62-1.97)	1.34 (1.23-1.46)	1.14 (1.08-1.20)	1.00 (REF)	0.99 (0.94-1.04)	1.03 (0.98-1.09)	1.26 (1.20-1.34)	1.59 (1.46-1.73)	2.33 (2.07-2.63)
Musculoskeletal	2.50 (2.04-3.05)	1.73 (1.44-2.07)	1.28 (1.13-1.45)	1.00 (REF)	0.94 (0.84-1.06)	0.90 (0.79-1.04)	1.11 (0.97-1.27)	1.62 (1.33-1.97)	2.27 (1.69-3.05)
Urogenital	1.34 (1.14-1.58)	1.08 (0.94-1.24)	0.99 (0.91-1.08)	1.00 (REF)	0.98 (0.91-1.06)	1.20 (1.11-1.29)	1.36 (1.26-1.48)	2.04 (1.81-2.30)	3.18 (2.66-3.79)
Accident– transport related	1.09 (0.72-1.66)	1.06 (0.79-1.44)	1.18 (0.98-1.42)	1.00 (REF)	0.97 (0.81-1.16)	0.89 (0.71-1.10)	1.11 (0.90-1.39)	0.76 (0.48-1.20)	1.31 (0.71-2.40)
Accident – non-transport	1.56 (1.36-1.80)	1.49 (1.34-1.66)	1.23 (1.14-1.33)	1.00 (REF)	0.88 (0.81-0.94)	0.83 (0.76-0.90)	0.90 (0.82-0.98)	1.05 (0.91-1.22)	1.32 (1.04-1.66)
Self-harm/violence	1.20 (0.92-1.58)	1.45 (1.22-1.74)	1.22 (1.08-1.38)	1.00 (REF)	0.90 (0.79-1.01)	0.75 (0.64-0.87)	0.79 (0.67-0.93)	0.62 (0.46-0.85)	0.66 (0.40-1.09)
**Specific Cancers**									
Oesophageal	1.80 (1.47-2.20)	1.40 (1.19-1.65)	1.07 (0.97-1.19)	1.00 (REF)	1.01 (0.93-1.10)	1.14 (1.04-1.25)	1.21 (1.09-1.33)	1.38 (1.18-1.62)	1.64 (1.27-2.12)
Gastric	1.30 (0.98-1.73)	1.29 (1.05-1.59)	1.11 (0.98-1.26)	1.00 (REF)	1.10 (0.99-1.22)	1.26 (1.13-1.41)	1.24 (1.10-1.40)	1.47 (1.21-1.79)	1.59 (1.14-2.21)
Colorectal	1.06 (0.89-1.25)	1.11 (0.98-1.25)	0.99 (0.92-1.06)	1.00 (REF)	1.05 (0.99-1.11)	1.10 (1.03-1.17)	1.21 (1.13-1.29)	1.37 (1.23-1.52)	1.53 (1.28-1.82)
Liver	0.88 (0.57-1.37)	1.12 (0.85-1.48)	0.87 (0.74-1.03)	1.00 (REF)	1.13 (0.99-1.28)	1.39 (1.21-1.59)	1.62 (1.41-1.85)	2.38 (1.98-2.87)	2.34 (1.74-3.15)
Pancreas	1.11 (0.89-1.40)	1.01 (0.85-1.19)	1.00 (0.91-1.10)	1.00 (REF)	1.08 (0.99-1.17)	1.10 (1.00-1.20)	1.19 (1.09-1.30)	1.51 (1.32-1.73)	1.06 (0.81-1.38)
Lung	1.46 (1.34-1.59)	1.35 (1.26-1.44)	1.12 (1.08-1.17)	1.00 (REF)	0.92 (0.88-0.95)	0.88 (0.84-0.92)	0.87 (0.83-0.91)	0.86 (0.79-0.93)	0.74 (0.63-0.87)
Melanoma	0.97 (0.61-1.55)	1.08 (0.80-1.47)	0.98 (0.82-1.17)	1.00 (REF)	1.02 (0.88-1.19)	1.09 (0.92-1.29)	1.14 (0.95-1.35)	0.85 (0.60-1.20)	1.72 (1.13-2.61)

Breast	0.92 (0.78-1.09)	0.88 (0.78-1.00)	0.97 (0.90-1.04)	1.00 (REF)	1.08 (1.00-1.15)	1.17 (1.09-1.27)	1.26 (1.17-1.36)	1.28 (1.15-1.43)	1.33 (1.12-1.57)
Uterus	1.30 (0.82-2.07)	0.67 (0.43-1.04)	0.82 (0.65-1.03)	1.00 (REF)	1.06 (0.87-1.29)	1.54 (1.26-1.88)	2.01 (1.66-2.42)	2.81 (2.21-3.55)	5.80 (4.47-7.52)
Ovarian	1.07 (0.80-1.42)	0.78 (0.62-0.98)	1.02 (0.90-1.15)	1.00 (REF)	1.06 (0.94-1.18)	1.12 (0.99-1.27)	1.17 (1.04-1.33)	1.21 (1.00-1.45)	1.67 (1.31-2.13)
Prostate	0.73 (0.54-0.99)	0.97 (0.81-1.16)	0.95 (0.87-1.03)	1.00 (REF)	1.03 (0.97-1.10)	1.12 (1.04-1.20)	1.05 (0.96-1.14)	1.06 (0.89-1.26)	1.19 (0.83-1.69)
Kidney	1.12 (0.77-1.62)	1.05 (0.80-1.37)	0.95 (0.82-1.11)	1.00 (REF)	1.06 (0.94-1.20)	1.31 (1.15-1.49)	1.52 (1.33-1.73)	1.95 (1.60-2.39)	2.78 (2.09-3.71)
Bladder	1.19 (0.89-1.58)	1.33 (1.09-1.63)	1.10 (0.98-1.25)	1.00 (REF)	1.02 (0.92-1.13)	1.17 (1.05-1.31)	1.23 (1.09-1.38)	1.23 (0.99-1.53)	1.83 (1.31-2.56)
Brain/CNS	0.91 (0.63-1.33)	0.99 (0.78-1.26)	0.97 (0.85-1.11)	1.00 (REF)	1.09 (0.97-1.22)	1.10 (0.96-1.25)	1.04 (0.91-1.19)	1.07 (0.85-1.34)	0.87 (0.58-1.32)
Haematological	0.75 (0.60-0.94)	0.92 (0.80-1.06)	1.03 (0.96-1.11)	1.00 (REF)	1.02 (0.96-1.09)	1.07 (0.99-1.14)	1.13 (1.05-1.21)	1.12 (0.99-1.28)	1.36 (1.11-1.67)
**Specific Endocrine**									
Diabetes	1.13 (0.84-1.53)	1.09 (0.87-1.38)	1.11 (0.97-1.27)	1.00 (REF)	1.21 (1.09-1.35)	1.14 (1.01-1.27)	1.56 (1.40-1.74)	2.55 (2.23-2.92)	3.28 (2.75-3.93)
**Specific Neurological**									
Dementia/Alzheimer’s	1.65 (1.53-1.78)	1.40 (1.31-1.49)	1.22 (1.17-1.27)	1.00 (REF)	0.88 (0.85-0.92)	0.80 (0.77-0.84)	0.79 (0.76-0.83)	0.75 (0.68-0.82)	0.94 (0.80-1.11)
**Specific CVD**									
Hypertensive heart disease	1.16 (0.78-1.74)	0.88 (0.62-1.24)	1.03 (0.85-1.24)	1.00 (REF)	1.31 (1.13-1.52)	1.66 (1.42-1.94)	2.27 (1.95-2.65)	3.62 (2.95-4.44)	7.85 (6.14-10.0)
Ischaemic heart disease	1.15 (1.07-1.23)	1.06 (1.00-1.12)	1.02 (0.98-1.05)	1.00 (REF)	1.05 (1.02-1.07)	1.20 (1.17-1.24)	1.36 (1.32-1.40)	1.80 (1.72-1.88)	2.49 (2.33-2.67)
Atrial flutter/fibrillation	1.20 (0.93-1.55)	1.18 (0.96-1.45)	1.11 (0.97-1.26)	1.00 (REF)	1.05 (0.94-1.17)	1.07 (0.94-1.21)	1.18 (1.03-1.34)	1.77 (1.45-2.16)	2.23 (1.59-3.13)
Heart Failure	1.21 (0.99-1.48)	1.03 (0.87-1.22)	1.00 (0.90-1.10)	1.00 (REF)	1.06 (0.97-1.16)	1.22 (1.11-1.35)	1.51 (1.37-1.66)	2.40 (2.08-2.77)	5.07 (4.18-6.16)
Cerebrovascular	1.29 (1.19-1.39)	1.24 (1.17-1.32)	1.08 (1.04-1.12)	1.00 (REF)	0.96 (0.93-1.00)	0.96 (0.93-1.00)	1.03 (0.99-1.07)	1.13 (1.05-1.21)	1.44 (1.28-1.62)
Aortic dissection	1.13 (0.73-1.74)	1.08 (0.78-1.50)	1.02 (0.84-1.24)	1.00 (REF)	0.97 (0.82-1.14)	0.95 (0.79-1.16)	1.18 (0.98-1.44)	0.84 (0.56-1.25)	1.32 (0.74-2.35)
Aortic aneurysm	1.02 (0.80-1.30)	0.89 (0.73-1.09)	0.85 (0.76-0.95)	1.00 (REF)	1.16 (1.06-1.26)	1.16 (1.05-1.28)	1.25 (1.12-1.39)	1.27 (1.04-1.55)	1.42 (0.99-2.05)
Peripheral vascular disease	1.61 (1.20-2.17)	1.69 (1.33-2.13)	1.28 (1.09-1.51)	1.00 (REF)	0.96 (0.82-1.12)	0.83 (0.69-0.99)	1.10 (0.92-1.31)	1.15 (0.83-1.59)	0.89 (0.44-1.81)
**Specific respiratory**									
Lower respiratory infection	1.83 (1.69-1.97)	1.43 (1.33-1.53)	1.18 (1.13-1.23)	1.00 (REF)	0.94 (0.90-0.98)	0.96 (0.92-1.01)	1.06 (1.01-1.11)	1.38 (1.28-1.50)	2.31 (2.05-2.61)
**Specific external**									
Falls	1.44 (1.12-1.86)	1.28 (1.05-1.57)	1.16 (1.02-1.31)	1.00 (REF)	0.89 (0.80-1.00)	0.80 (0.70-0.91)	0.83 (0.72-0.96)	0.94 (0.73-1.21)	1.19 (0.79-1.79)
Suicide	1.20 (0.91-1.58)	1.46 (1.22-1.75)	1.21 (1.07-1.37)	1.00 (REF)	0.89 (0.79-1.01)	0.74 (0.63-0.86)	0.78 (0.66-0.91)	0.61 (0.45-0.84)	0.67 (0.40-1.10)

Note: 5-year exclusion period after BMI applied in all models; estimates adjusted for age, deprivation, calendar year, diabetes, alcohol status, smoking (all as defined at date of BMI measure), and stratified for gender

Table S2.8: Association between BMI (in 9 categories) and all-cause mortality by ethnicity , among never-smokers

	**Hazard Ratio**								
BMI category (kg/m2)	*<18.5*	*18.5-19.9*	*20.0-22.4*	*22.5-24.9*	*25.0-27.4*	*27.5-29.9*	*30.0-34.9*	*35.0-39.9*	*≥40.0*
White	1.77 (1.64, 1.91)	1.40 (1.32, 1.48)	1.14 (1.10, 1.18)	1.00 (REF)	1.02 (0.99, 1.05)	1.08 (1.04, 1.12)	1.20 (1.16, 1.25)	1.57 (1.49, 1.65)	2.34 (2.17, 2.53)
S Asian	1.14 (0.77, 1.67)	1.27 (0.90, 1.78)	1.17 (0.94, 1.44)	1.00 (REF)	1.11 (0.92, 1.35)	1.06 (0.85, 1.32)	1.22 (0.99, 1.52)	1.55 (1.11, 2.15)	2.77 (1.79, 4.31)
Black	1.54 (0.67, 3.51)	2.01 (1.21, 3.34)	1.00 (0.68, 1.46)	1.00 (REF)	1.20 (0.91, 1.57)	1.03 (0.77, 1.38)	1.27 (0.96, 1.67)	1.35 (0.93, 1.97)	2.15 (1.35, 3.40)
Other	2.43 (1.23, 4.77)	1.12 (0.58, 2.14)	0.97 (0.63, 1.49)	1.00 (REF)	1.13 (0.76, 1.67)	0.97 (0.62, 1.51)	1.38 (0.91, 2.11)	2.02 (1.10, 3.71)	2.40 (1.03, 5.59)
Mixed	5.38 (1.21, 23.9)	2.40 (0.85, 6.73)	1.18 (0.55, 2.56)	1.00 (REF)	1.35 (0.66, 2.75)	1.04 (0.44, 2.43)	1.17 (0.51, 2.67)	2.08 (0.74, 5.84)	3.50 (1.00, 12.3)

Note: 5-year exclusion period applied for person-time and events after a BMI record; estimates adjusted for age at BMI record, calendar year, diabetes, alcohol status (all as defined at date of BMI measure), and stratified for gender

Table S2.9: Deaths attributable to suboptimal BMI (assuming causality), and population attributable fractions for all-cause mortality, among never-smokers

BMI category (kg/m²)	*N deaths observed*	*% of all deaths*	*Adjusted hazard ratio*	*Population attributable fraction (%)**	*N deaths attributable*
<18.5	4207	2.2	1.42	0.7	28
18.5-19.9	7001	3.7	1.2	0.6	43
20.0-22.4	26114	13.9	1.07	0.9	228
22.5-24.9 (REF)	43097	22.9	1.00 (REF)	0	0
25.0-27.4	43151	22.9	1.01	0.3	125
27.5-29.9	28711	15.3	1.07	0.9	272
30.0-34.9	25672	13.7	1.2	2.3	596
35.0-39.9	7187	3.8	1.48	1.2	89
>=40	2917	1.6	2.1	0.8	24
Total	188057	100.0	-	-	-
Totals attributable					
Overweight including obesity (≥25)	-	-	-	*5.5*	*1106*
Obesity (≥30)	-	-	-	*4.3*	*709*
All suboptimal (<22.5 or ≥25)				*7.5*	*1405*

** calculated as p_d*(HR-1)/(HR) where p_d is the proportion of all observed deaths that were in that category, HR is the hazard ratio for that category*

Table S2.10: Cumulative incidence of the four most common cause-specific mortality outcomes among never-smokers at specific ages, by BMI category

	Cumulative incidence (%) of cause-specific/overall mortality at age (yrs)…				
	50	60	70	80	90
**Underweight (BMI<18.5kg/m^2)**					
Cancer	0.7	1.9	4.3	9.7	14.7
Cardiovascular	0.1	0.8	2.1	8.1	21.1
Neurological	0.2	0.5	1.4	4.7	11.7
Respiratory	0.1	0.3	1.4	4.9	9
Other	1.1	2.1	4.1	10.4	21.4
**Healthy weight (18.5≤BMI<25kg/m^2)**					
Cancer	0.6	1.7	4.2	9.8	17.6
Cardiovascular	0.2	0.5	1.5	6.3	21
Neurological	0.1	0.2	0.5	2.1	7.8
Respiratory	0	0.1	0.2	1.2	3.7
Other	0.6	1	1.8	4.3	13.3
**Overweight (25≤BMI<30kg/m^2)**					
Cancer	0.6	1.8	4.7	11.1	19.6
Cardiovascular	0.3	0.8	2.3	8	22.8
Neurological	0.1	0.2	0.4	1.6	6
Respiratory	0	0.1	0.2	1	3.2
Other	0.7	1.2	2.1	4.6	13
**Obese (BMI>30kg/m^2)**					
Cancer	0.7	2.1	5.6	12.3	19.9
Cardiovascular	0.6	1.6	3.9	11.2	25.9
Neurological	0.1	0.2	0.5	1.5	5.3
Respiratory	0.1	0.2	0.5	1.5	3.5
Other	1.1	1.9	3.4	7.3	16.8

Note: 5-year exclusion period applied for person-time and events after a BMI record; cumulative incidence figures for cause-specific mortality outcomes treat deaths from other causes as competing risks

Table S2.11: Association between BMI (in 9 categories) and CVD, cancer, respiratory, neurological, mental/behavioural and self-harm/violent mortality among never-smokers, including/excluding those with prevalent disease at start of time at risk

	**Hazard Ratio**								
BMI category (kg/m2)	*<18.5*	*18.5-19.9*	*20.0-22.4*	*22.5-24.9*	*25.0-27.4*	*27.5-29.9*	*30.0-34.9*	*35.0-39.9*	*≥40.0*
Cardiovascular									
Ignore prior disease	1.18 (1.11, 1.26)	1.11 (1.06, 1.16)	1.03 (1.00, 1.06)	1.00 (REF)	1.04 (1.02, 1.07)	1.14 (1.11, 1.17)	1.32 (1.28, 1.35)	1.75 (1.67, 1.82)	2.68 (2.51, 2.86)
Exclude prior disease	1.21 (1.12, 1.31)	1.17 (1.10, 1.24)	1.02 (0.98, 1.06)	1.00 (REF)	1.06 (1.02, 1.09)	1.15 (1.11, 1.19)	1.37 (1.32, 1.42)	1.86 (1.76, 1.97)	3.12 (2.88, 3.38)
Cancer									
Ignore prior disease	1.05 (0.96, 1.14)	0.97 (0.92, 1.03)	0.99 (0.96, 1.02)	1.00 (REF)	1.08 (1.05, 1.11)	1.15 (1.12, 1.19)	1.25 (1.21, 1.29)	1.38 (1.32, 1.46)	1.60 (1.48, 1.73)
Exclude prior disease	1.06 (0.96, 1.16)	0.95 (0.89, 1.01)	0.98 (0.95, 1.02)	1.00 (REF)	1.09 (1.06, 1.13)	1.18 (1.14, 1.21)	1.28 (1.24, 1.33)	1.48 (1.40, 1.56)	1.74 (1.60, 1.89)
Respiratory									
Ignore prior disease	2.60 (2.32, 2.92)	1.73 (1.56, 1.91)	1.21 (1.13, 1.29)	1.00 (REF)	0.92 (0.87, 0.98)	0.96 (0.89, 1.03)	1.09 (1.02, 1.18)	1.36 (1.21, 1.53)	2.28 (1.91, 2.71)
Exclude prior disease	2.29 (2.00, 2.62)	1.58 (1.41, 1.78)	1.17 (1.09, 1.27)	1.00 (REF)	0.95 (0.89, 1.01)	1.04 (0.97, 1.12)	1.15 (1.06, 1.25)	1.37 (1.20, 1.56)	2.45 (2.03, 2.96)
Neurological									
Ignore prior disease	1.75 (1.61, 1.90)	1.43 (1.33, 1.53)	1.19 (1.14, 1.25)	1.00 (REF)	0.89 (0.85, 0.93)	0.83 (0.79, 0.87)	0.82 (0.77, 0.86)	0.78 (0.70, 0.86)	1.04 (0.89, 1.23)
Exclude prior disease	1.68 (1.53, 1.84)	1.39 (1.29, 1.50)	1.19 (1.13, 1.25)	1.00 (REF)	0.91 (0.87, 0.95)	0.85 (0.81, 0.89)	0.84 (0.80, 0.89)	0.81 (0.73, 0.90)	1.10 (0.93, 1.30)
Mental/behavioural									
Ignore prior disease	3.97 (2.25, 7.00)	2.44 (1.45, 4.10)	1.60 (1.09, 2.33)	1.00 (REF)	0.80 (0.53, 1.19)	0.92 (0.59, 1.43)	1.27 (0.83, 1.94)	0.83 (0.36, 1.95)	(too few events)
Exclude prior disease	3.97 (2.00, 7.90)	2.12 (1.09, 4.09)	1.66 (1.06, 2.61)	1.00 (REF)	0.83 (0.51, 1.34)	0.74 (0.42, 1.32)	1.17 (0.69, 1.99)	1.11 (0.43, 2.84)	(too few events)
Self-harm/violent									
Ignore prior disease	1.10 (0.66, 1.84)	1.45 (1.07, 1.98)	1.22 (1.00, 1.49)	1.00 (REF)	1.15 (0.96, 1.38)	0.91 (0.72, 1.14)	0.93 (0.73, 1.18)	0.68 (0.42, 1.10)	0.73 (0.34, 1.56)
Exclude prior disease	0.98 (0.51, 1.87)	1.54 (1.06, 2.22)	1.21 (0.95, 1.55)	1.00 (REF)	1.12 (0.89, 1.40)	0.99 (0.75, 1.29)	0.91 (0.67, 1.23)	0.57 (0.29, 1.12)	0.74 (0.27, 2.00)

Note: 5-year exclusion period applied for person-time and events after a BMI record; estimates adjusted for age at BMI record, calendar year, diabetes, alcohol status (all as defined at date of BMI measure), and stratified for gender

Figure S2.1: Participant flow diagram

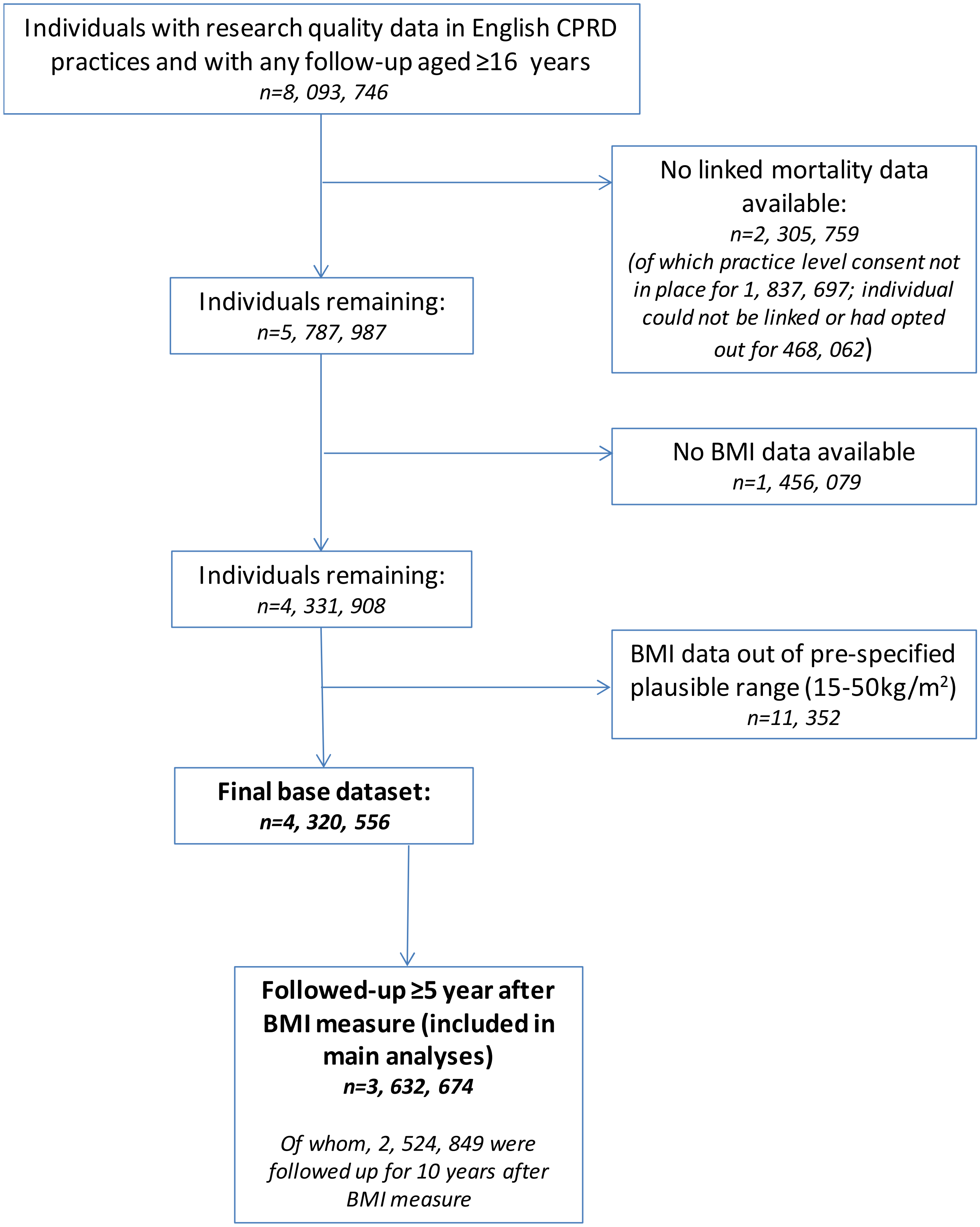

Figure S2.2: *Association between BMI and level 2 and 3 cause-specific mortality outcomes among total study population including ever-smokers*

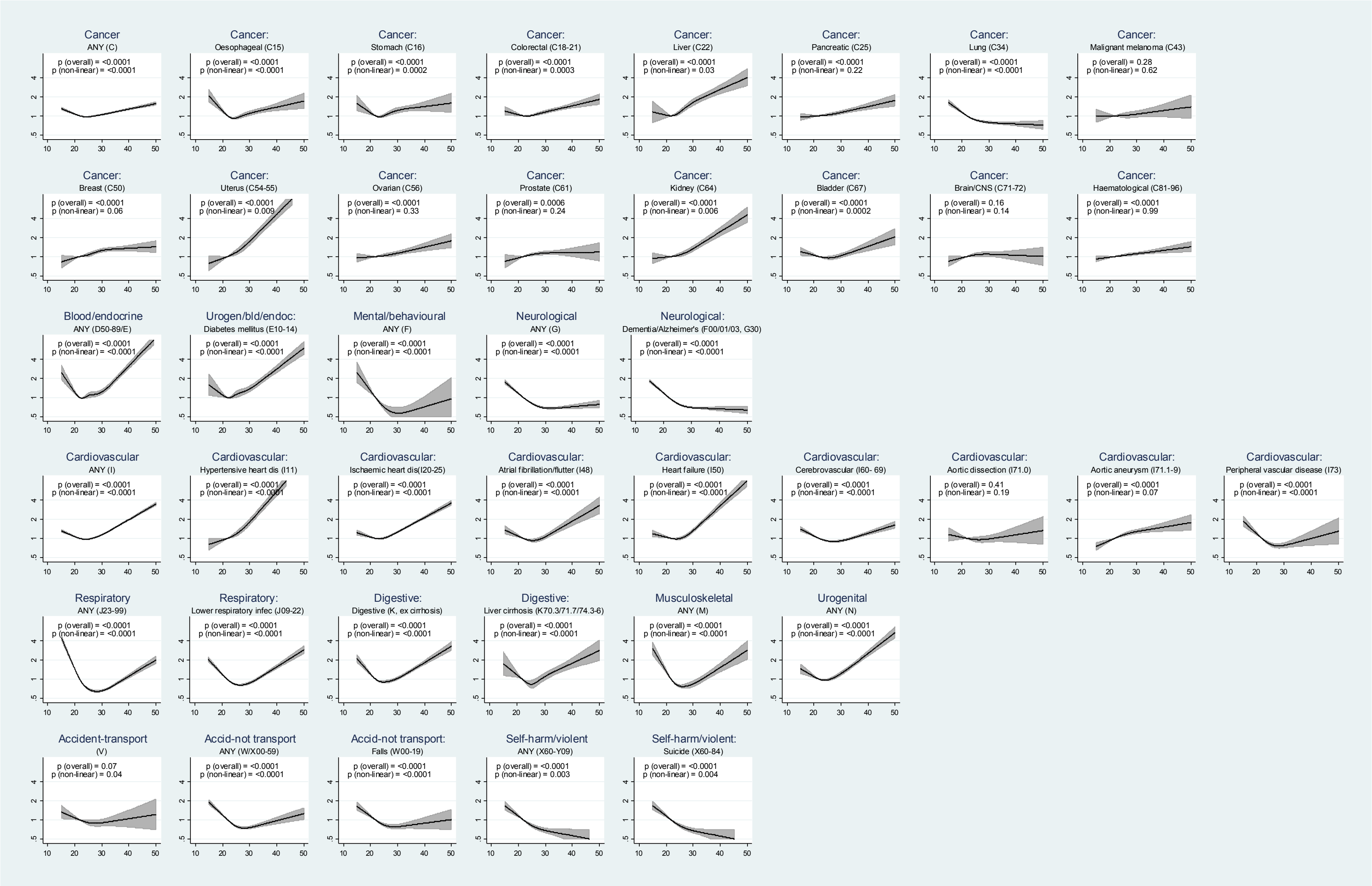

Notes: x-axes are BMI in kg/m2, y-axes are hazard ratios (95% CIs). 5-year exclusion period applied for person-time and events after a BMI record; estimates adjusted for age, deprivation, calendar year, diabetes, smoking, alcohol status (all as defined at date of BMI measure, and stratified for gender; abbreviation urogen/bld/endoc = urogenital/blood/endocrine

Figure S2.3: Association between BMI and mortality outcomes, by smoking

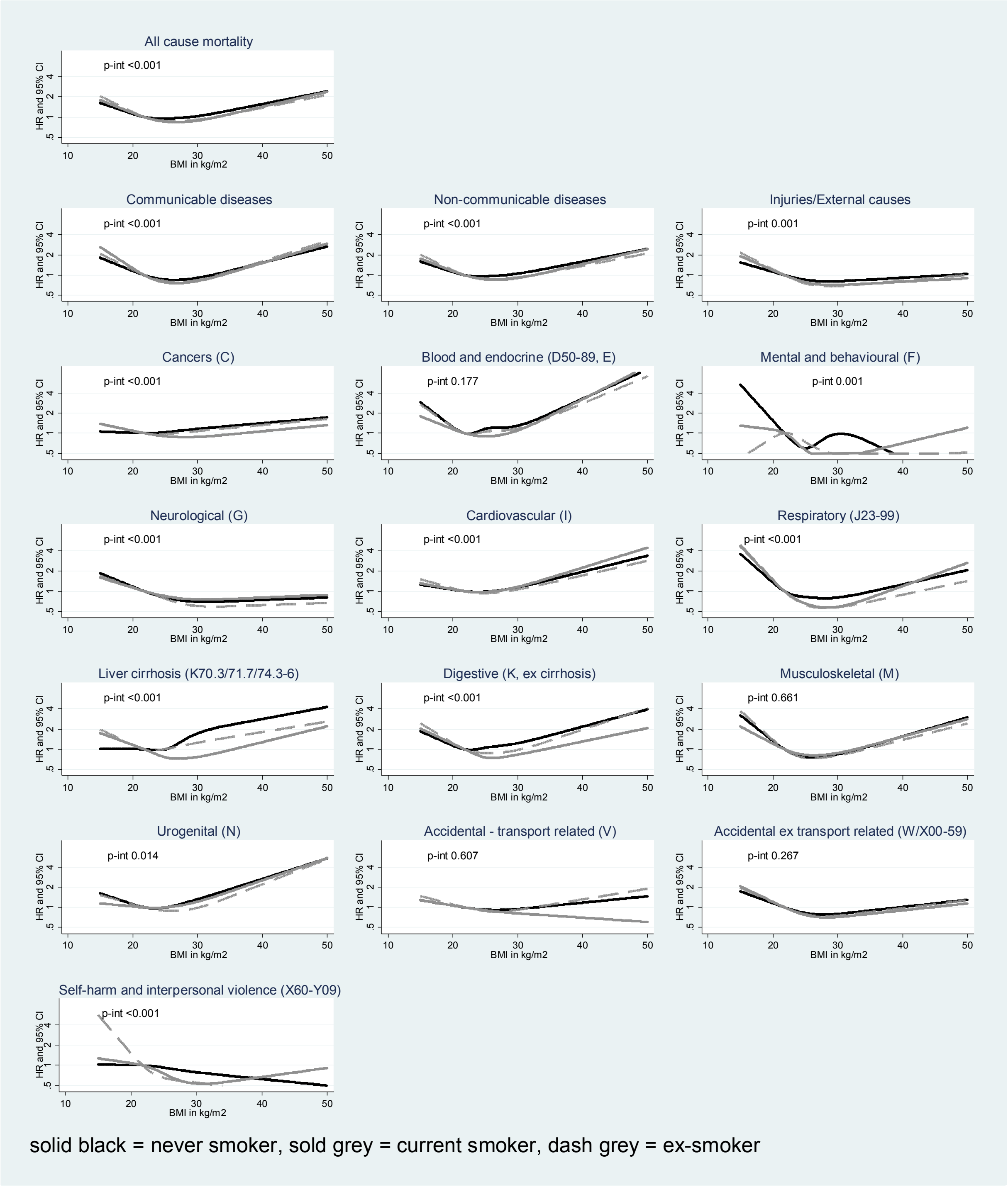

Note: 5-year exclusion period applied for person-time and events after a BMI record; estimates adjusted for age at BMI record, deprivation, calendar year, diabetes, alcohol status (all as defined at date of BMI measure), and stratified for gender

Figure S2.4: Association between BMI and mortality outcomes among never-smokers, by gender

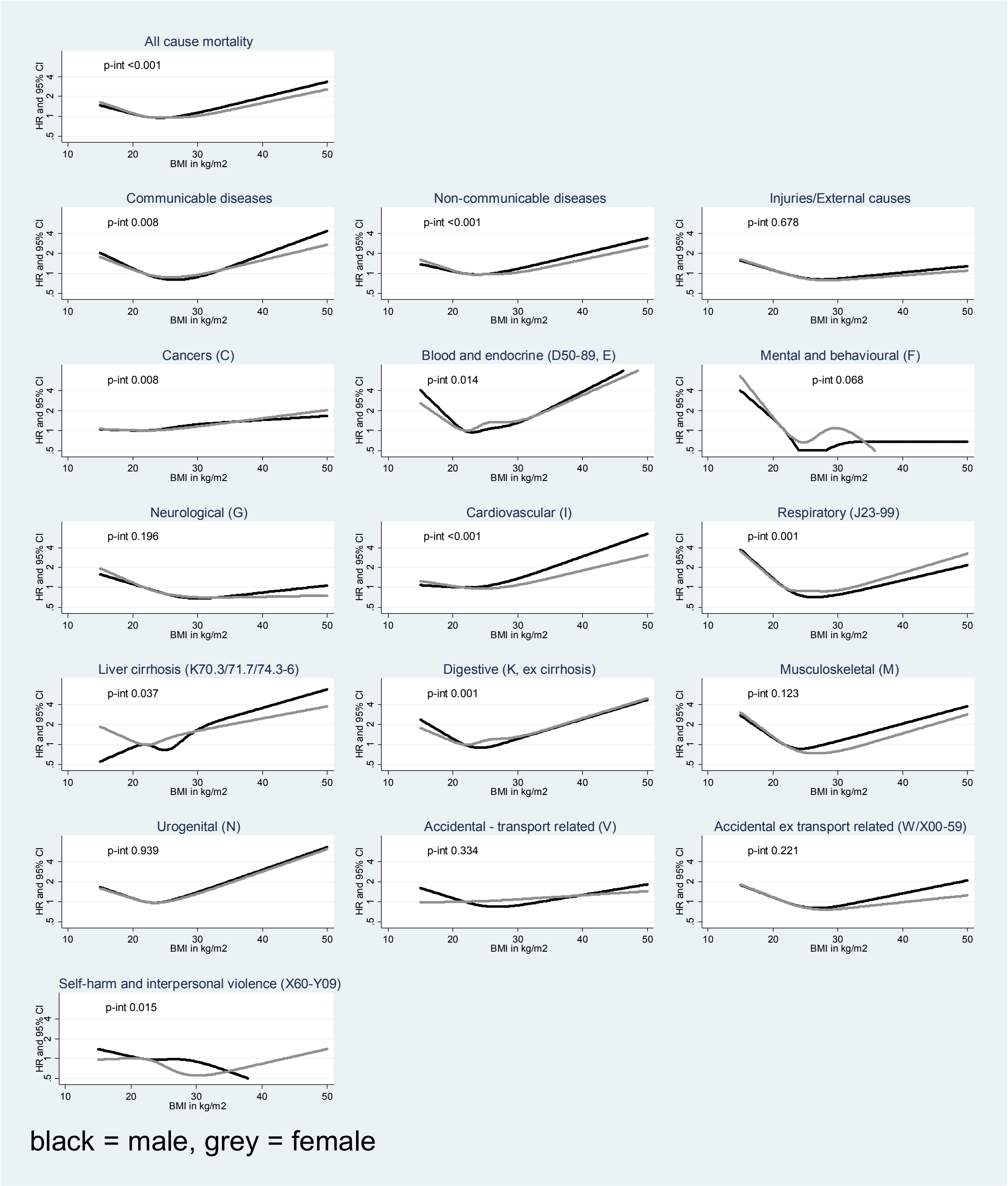

Note: 5-year exclusion period applied for person-time and events after a BMI record; estimates adjusted for age at BMI record, deprivation, calendar year, diabetes, alcohol status (all as defined at date of BMI measure)

Figure S2.5: Association between BMI and mortality outcomes among never-smokers, by current age

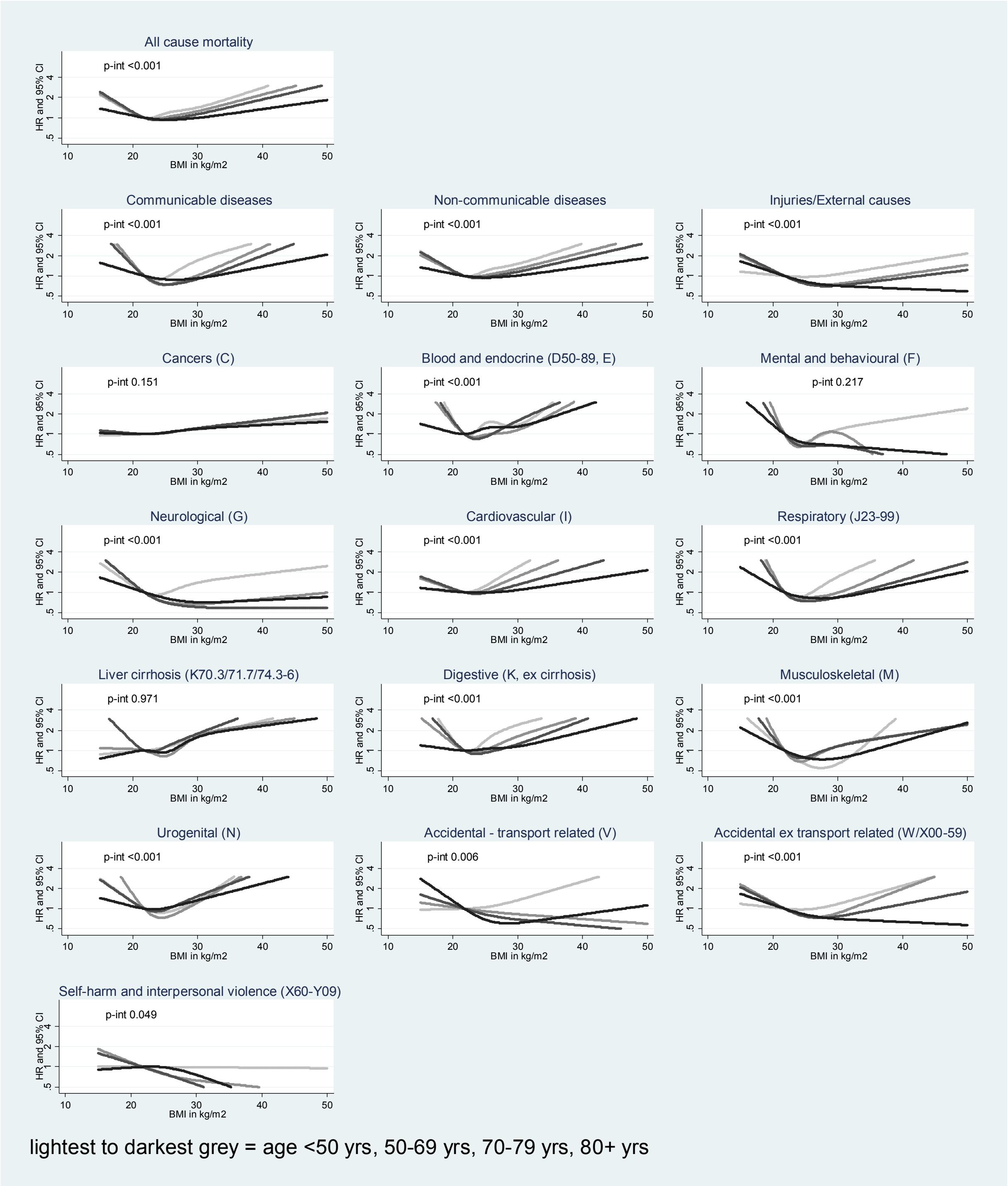

Note: 5-year exclusion period applied for person-time and events after a BMI record; estimates adjusted for age at BMI record, calendar year, diabetes, alcohol status (all as defined at date of BMI measure), and stratified for gender

Figure S2.6: Association between BMI and mortality outcomes among never-smokers, by deprivation quintile

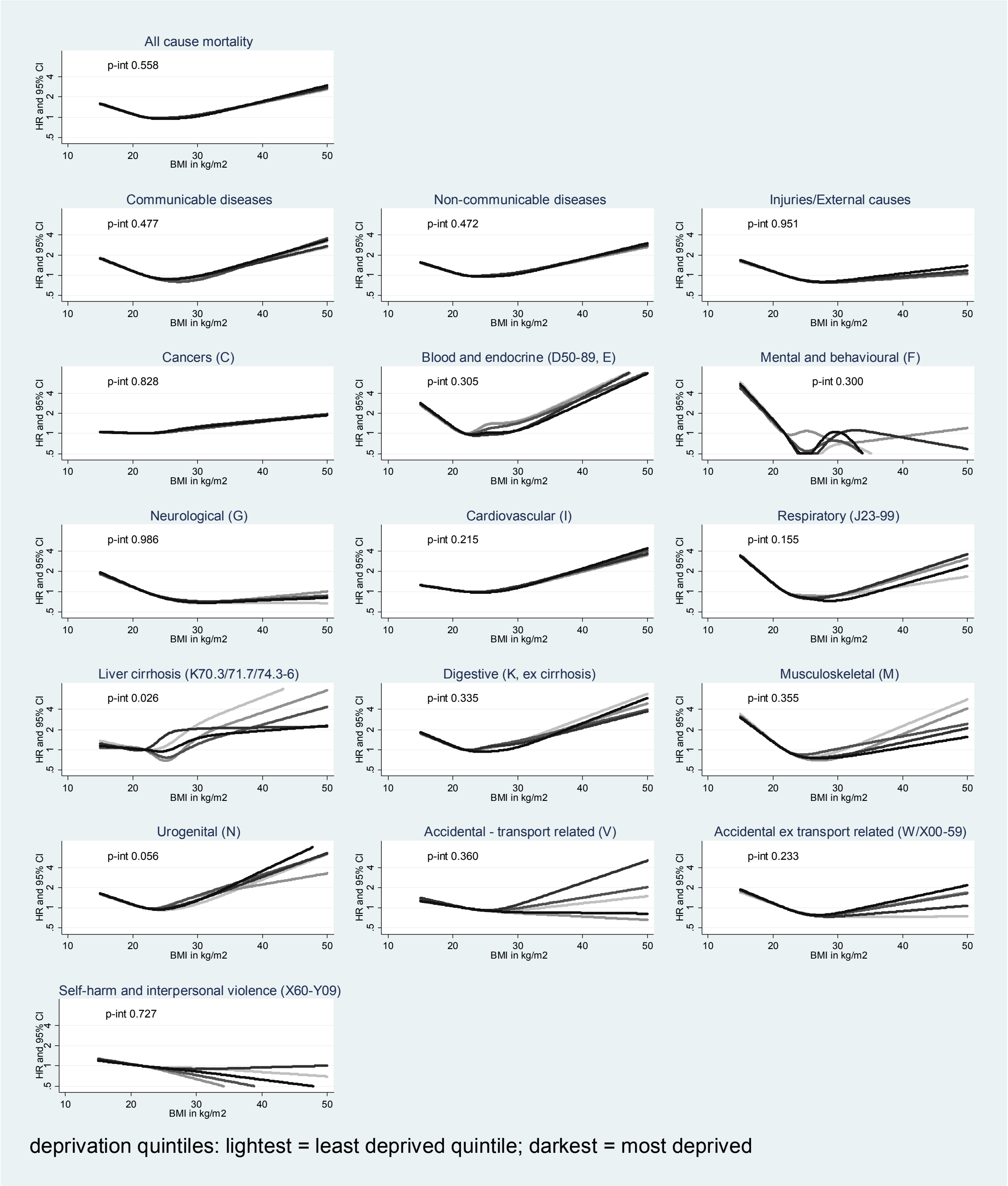

Note: 5-year exclusion period applied for person-time and events after a BMI record; estimates adjusted for age at BMI record, calendar year, diabetes, alcohol status (all as defined at date of BMI measure), and stratified for gender

Figure S2.7: Association between BMI and all-cause mortality among never-smokers, by ethnicity

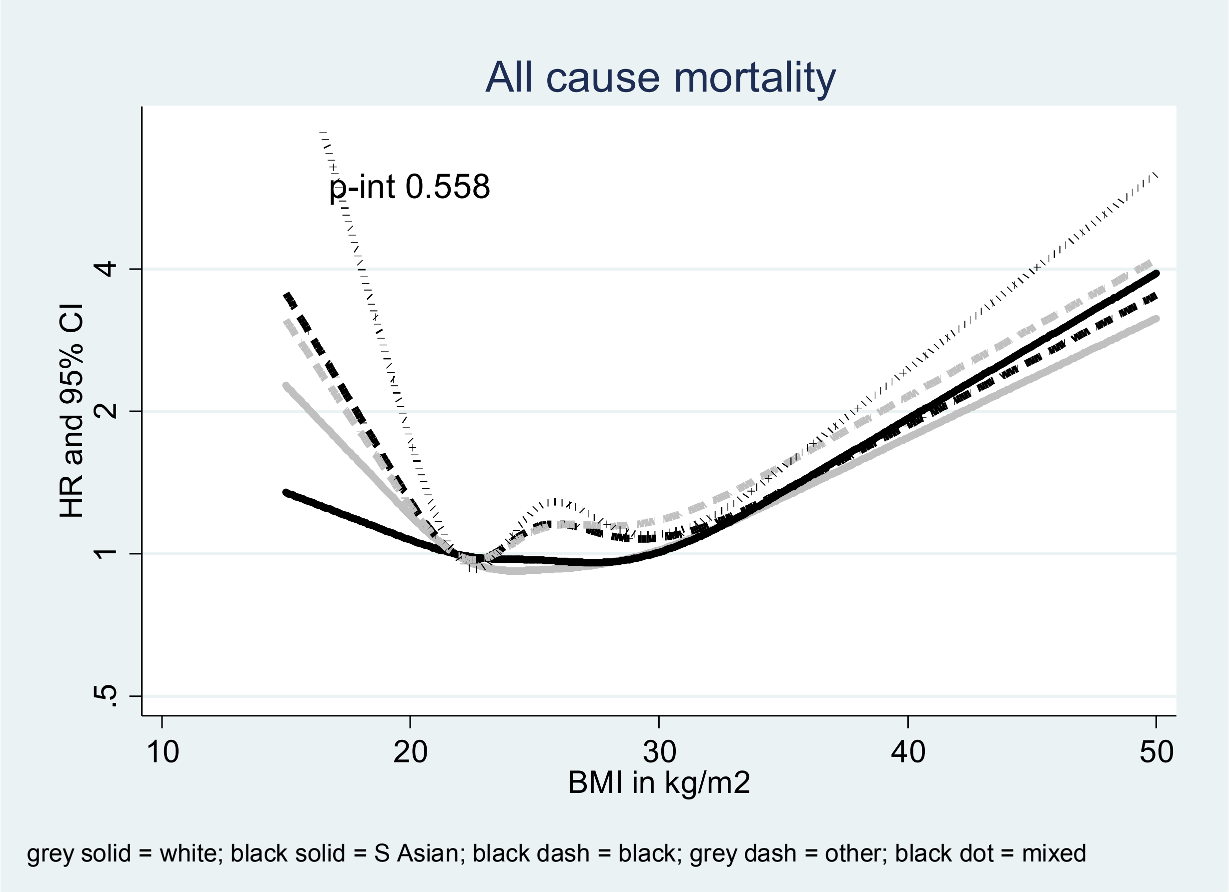

Note: 5-year exclusion period applied for person-time and events after a BMI record; estimates adjusted for age at BMI record, calendar year, diabetes, alcohol status (all as defined at date of BMI measure), and stratified for gender

Figure S2.8: Cumulative incidence of the four most common cause-specific mortality outcomes among never-smokers, by BMI category

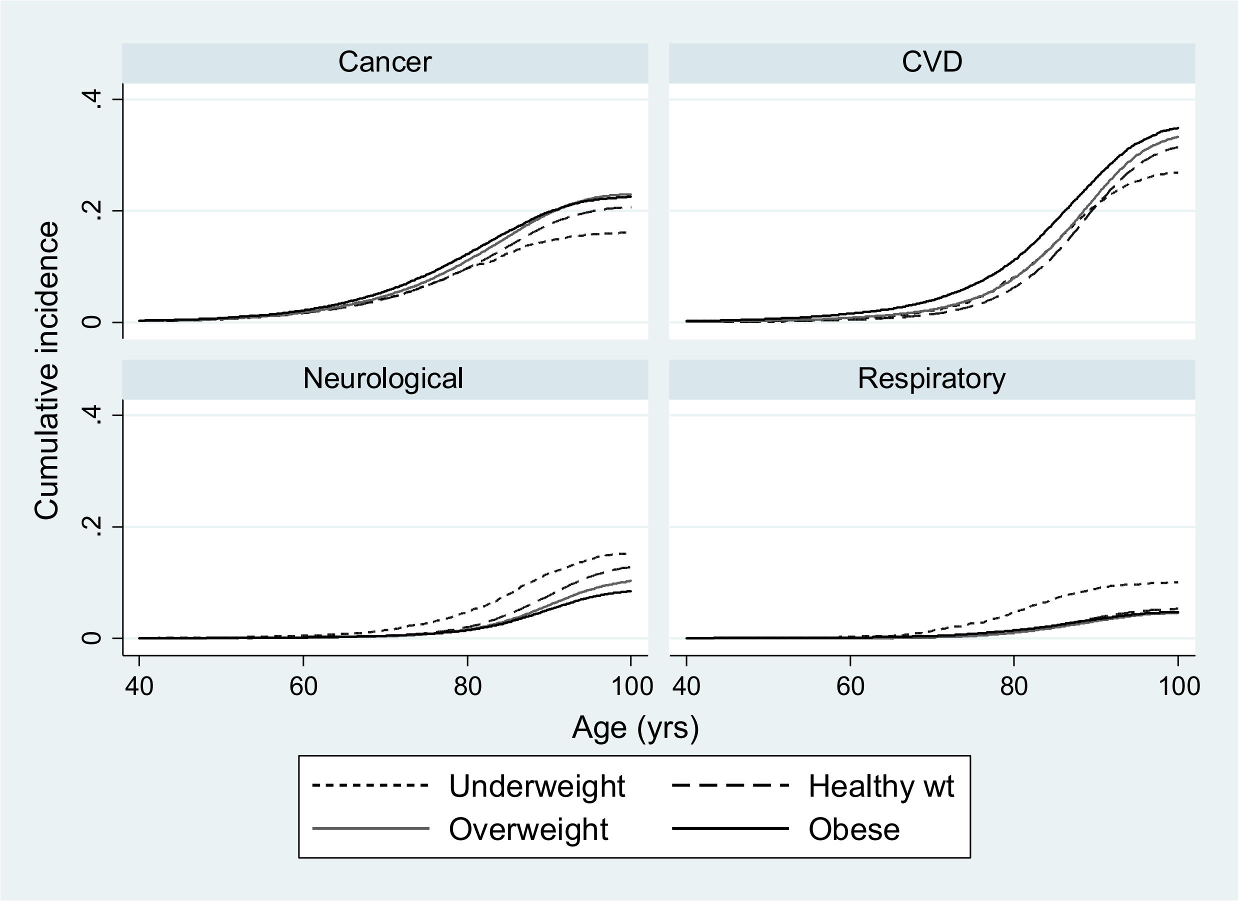

Figure S2.9: Association between BMI and all-cause/cause-specific mortality outcomes, with various lengths of excluded follow-up time post-BMI record, in never-smokers

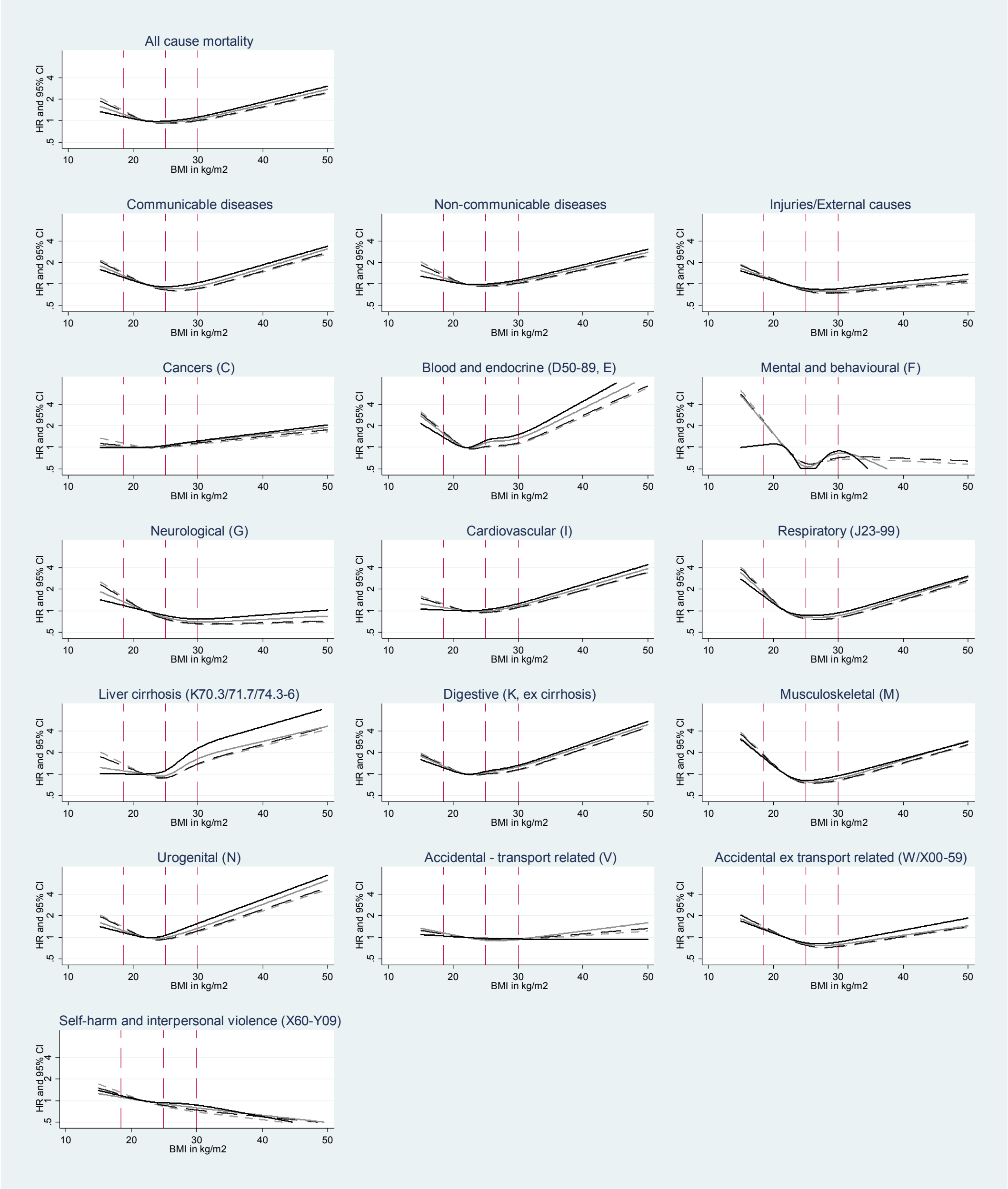

Solid black = 10 year exclusion; solid grey = 5y, dashed black = 1y, dashed grey = 0y
Dashed vertical lines represent WHO BMI category thresholds of 18.5 (underweight to healthy), 25 (healthy weight to overweight), 30 (overweight to obese)
Estimates adjusted for age at BMI record, deprivation, calendar year, diabetes, alcohol status, smoking (all as defined at date of BMI measure, and stratified for gender

Figure S2.10: Association between BMI and CVD, cancer, respiratory, neurological, mental/behavioural and self-harm/violent mortality among never-smokers, excluding or including those with prevalent disease at start of time at risk

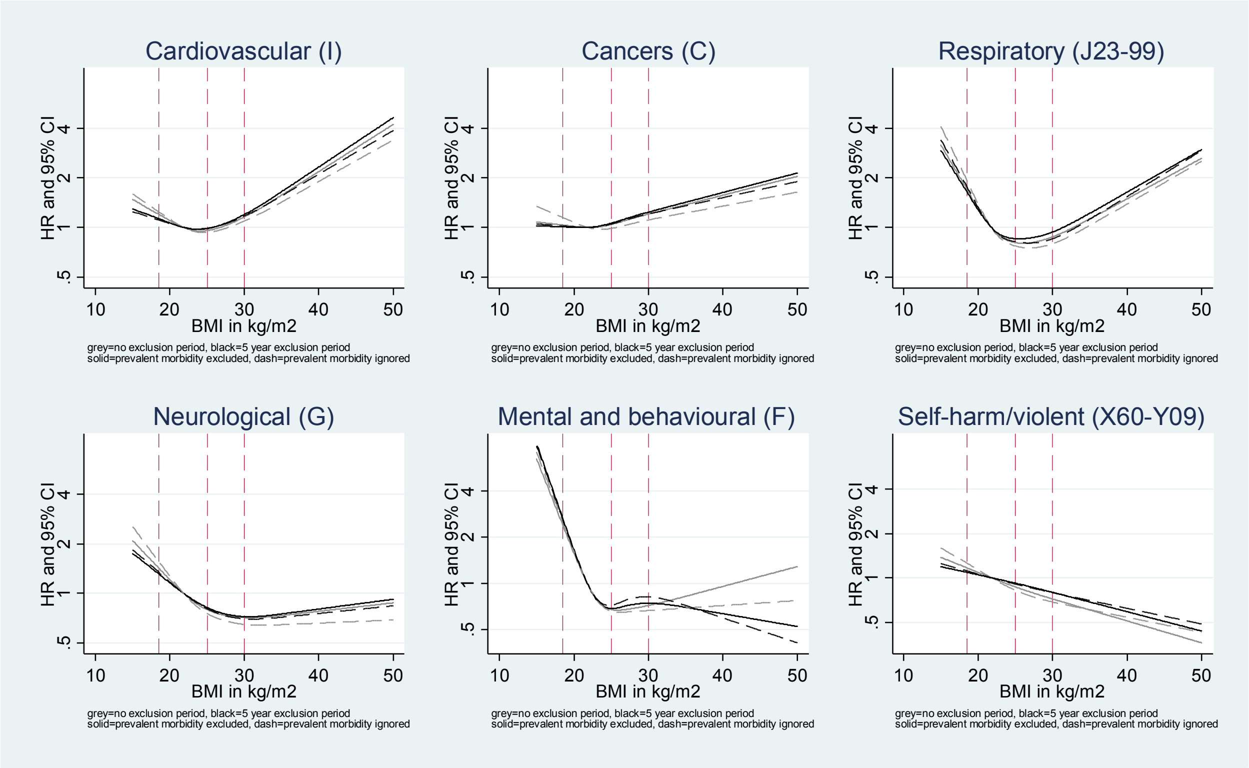

Note: estimates adjusted for age at BMI record, calendar year, diabetes, alcohol status (all as defined at date of BMI measure), and stratified for gender

Figure S2.11: Association between BMI and all-cause mortality in various sensitivity analyses

(i) *Different ways of parametrising BMI*

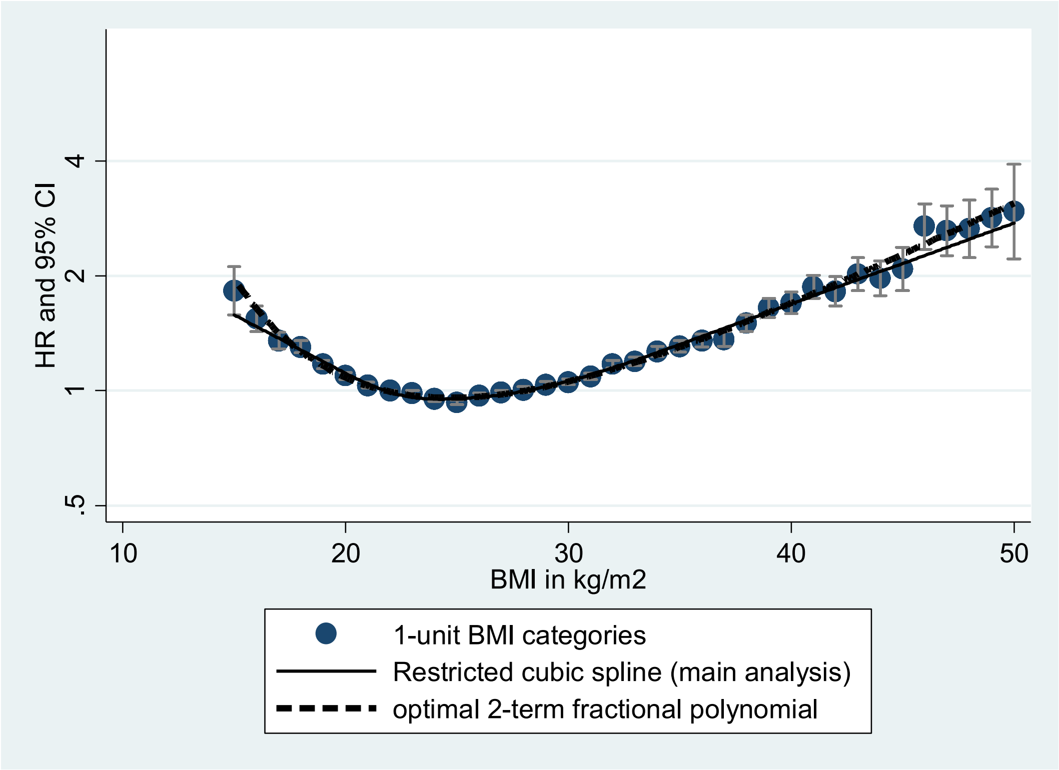

(ii) *Handling of ethnicity*

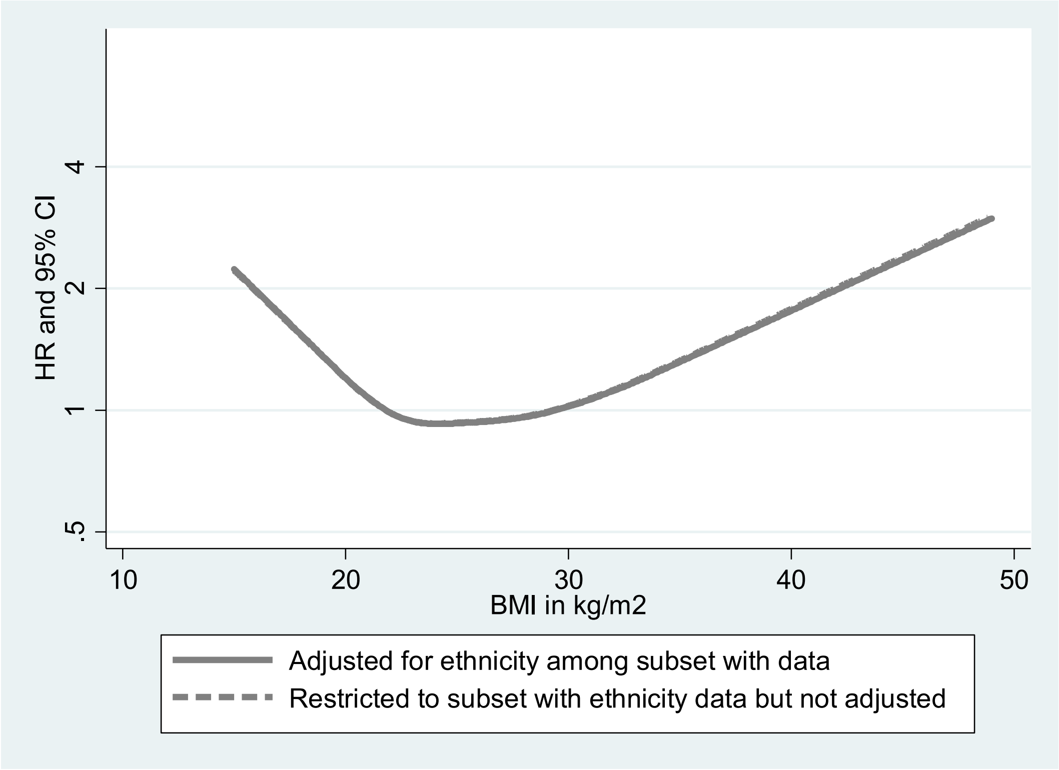

(iii) Handling of diabetes

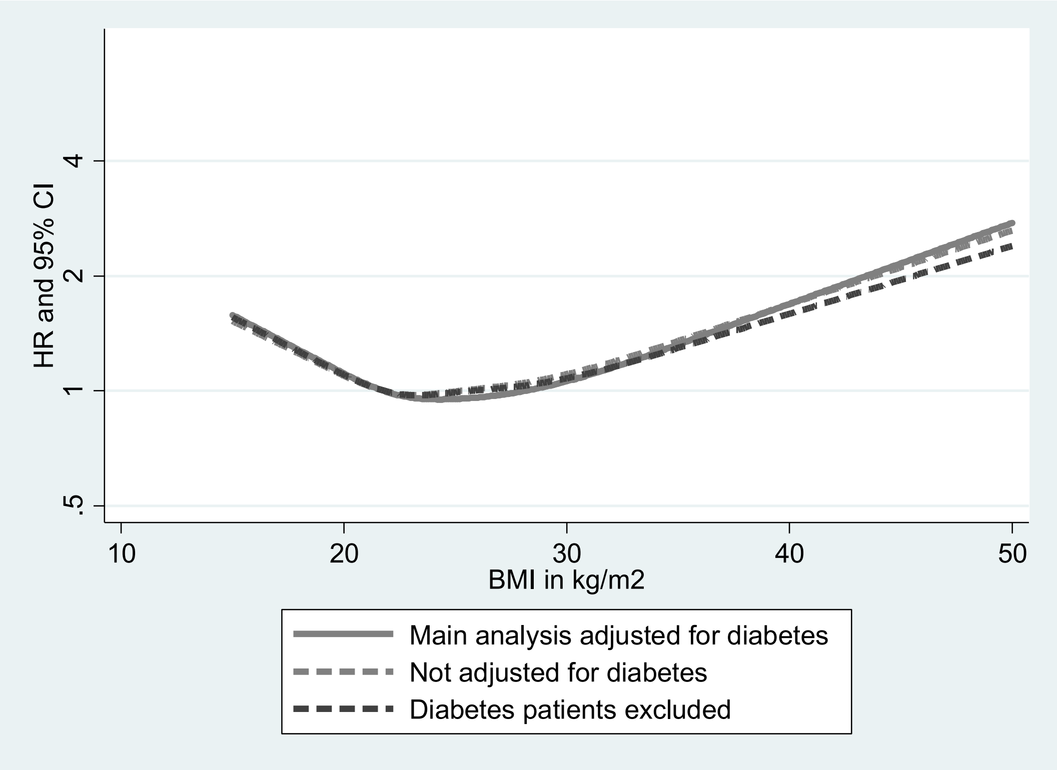

(iv) Analyses to investigate impact of selective BMI missingness

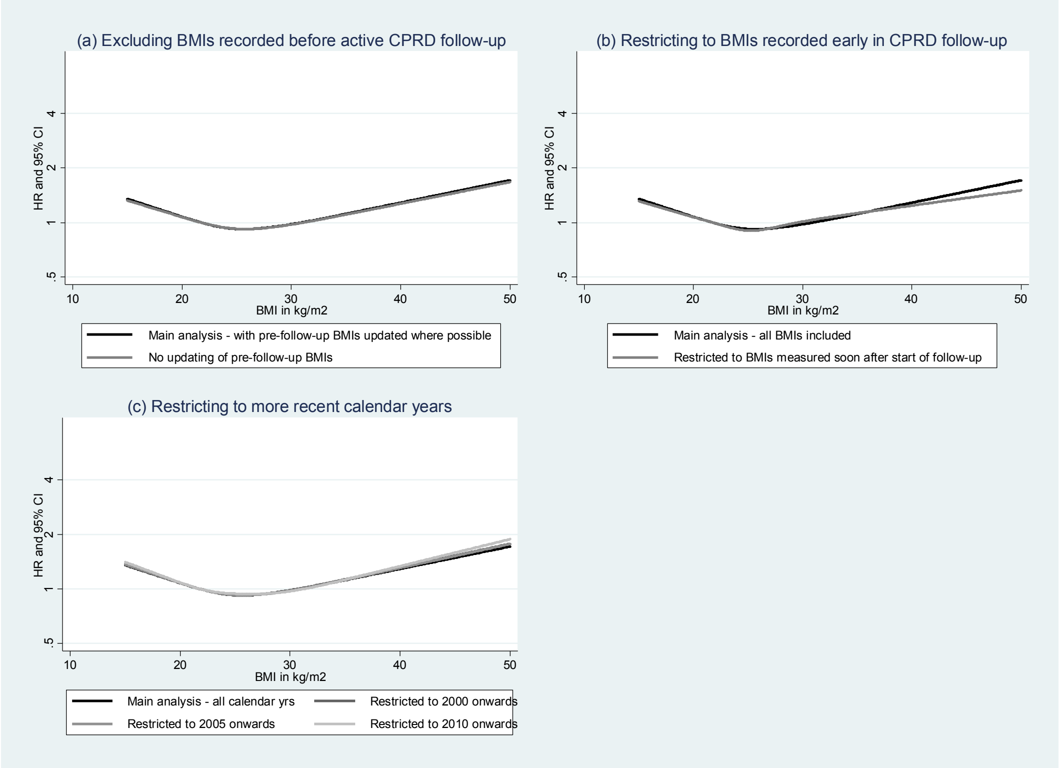

Part 3 – original study protocol

(as approved by the Independent Scientific Advisory Committee for MHRA Database Research (ISAC) on 24th August 2016; ; deviations from protocol with justification are listed at the end)

Protocol:

Body mass index and cause-specific morality - population-based cohort study using record linkage

A. Lay Summary (Max. 200 words)

Previous studies have shown that body mass index (BMI), a measure that captures a person's weight taking into account their height, is associated with the overall risk of dying at a given age. However, few large studies have explored how BMI is related to dying from specific causes. Our objective is to comprehensively investigate how BMI is associated with broad and specific causes of death. We will used primary care data (which includes information on a person's BMI) linked to cause of death data as recorded on death certificates. We will fit statistical models to look at how BMI is associated with each of a wide range of causes of death, taking into account information on other factors like smoking that could be linked to both BMI and the risk of dying from particular diseases. We will look at whether the role of BMI is different for different subgroups of the population. Finally, within groups of people with similar BMI levels, we will estimate the proportion dying from different causes at specific ages.

B. Technical Summary (Max. 200 words)

Body mass index (BMI) is associated with all-cause mortality, but few large studies have explored associations with death from specific causes. Our objective is to comprehensively investigate associations between BMI and specific causes of death. We will used CPRD primary care data linked to Office of National Statistics mortality data. All individuals with a BMI record in the Clinical Practice Research Datalink (CPRD) will be included. Outcomes will be underlying causes of death, using categorisations developed as part of the Global Burden of Disease project. We will look at both broad and specific categories/groupings of causes of death. We will use Cox regression models based on cause-specific hazards to model the associations between BMI and each cause-specific mortality outcome, adjusting for key potential confounders. We will use cubic splines to allow for non-linearity, and we will fit interactions to investigate effect modification by individual level factors. We will also use competing risks methods to estimate cumulative incidences for each outcome, stratified by BMI category.

C. Objectives, Specific Aims and Rationale

The broad research objective is to provide a comprehensive description of the associations between BMI and death from a full range of different causes.

The specific aims are:

- To estimate the association between BMI and all-cause mortality
- To estimate associations between BMI and cause-specific mortality outcomes, with causes of death divided both into broad groupings, and into more specific disease categories
- To estimate the cumulative incidence of death from specific causes, within strata of BMI, allowing for other causes of death as competing risks

D. **Background**

A number of major papers have investigated the associations between BMI and all-cause mortality. The Prospective Studies Collaboration[1] pooled individual data from 57 prospective studies (N=900,000) and Berrington de Gonzalez et al[2] pooled data from 19 prospective studies, (N=1.46 million); both found an approximately U-shaped relationship between BMI and hazard of death, with increases in risk for both underweight and overweight/obese individuals, compared to those with healthy weight. However, Flegal et al, in a recent systematic review, challenged the developing orthodoxy, suggesting that overweight was associated with a reduced risk of death.[3]

Of the above studies, only the Prospective Studies Collaboration looked into cause-specific mortality.[1] The authors fitted linear models stratified by BMI below/above 25 kg/m^2 to separate out effects among the underweight/healthy weight vs overweight/obese. They also looked in more detail at the shape of the relationship for certain key causes of death (ischaemic heart disease, stroke, all vascular, lung/oral/oesophageal cancer, other cancer, all respiratory, chronic obstructive pulmonary disease), estimated overall life span by BMI category, and modelled cause-specific cumulative mortality by combining estimated relative risks with cause-specific published death rates. We intend to build on the knowledge generated in this study in the following ways: (i) our study is anticipated to be 2 to 3 times larger, increasing the precision and enabling us to look at a wider range of cause of death outcomes; (ii) we will be using a single large contemporary population-based cohort with largely measured BMI data, and with uniform inclusion and exclusion criteria; (iii) we plan to fit a fully flexible non-linear model for all categories of cause of death, rather than only selected ones; (iv) we will consider a much broader range of individual specific causes of death; (v) we will systematically investigate whether a broad range of possible confounders such as age, sex, comorbidities, smoking, alcohol use, ethnicity, and socioeconomic status affect estimated associations; (vi) we will directly estimate cumulative incidences of each cause-specific mortality outcome by BMI status, using competing risks methods.

A number of other studies have also looked at the associations between BMI and a more limited range of mortality outcomes or at relationships with disease incidence. For example, Nordestgaard et al used a Mendelian randomisation approach to demonstrate a convincing causal link between higher BMI and ischaemic heart disease risk.[4] Parr et al used data from the Asia-Pacific Cohort Studies Collaboration to show an increased risk among overweight and obese people of death from cancer overall, and from specific cancers including colorectal, ovarian, cervical, prostate, and leukaemia.[5] In our own previous work, published in the Lancet, we used CPRD data to comprehensively describe the relationship between BMI and site-specific cancer incidence (previous ISAC protocol number 12_090A2).[6] We used similar methods to those proposed for the present study to investigate non-linear associations between BMI and cancer outcomes, and to investigate effect modification by individual-level factors. We found strong effects of BMI on cancer risk, with considerable variation in the nature of the effects by cancer site. The methods and algorithms we developed and the experience gained in doing this work leave us well placed to conduct this new proposed study.

E. Study Type

This is an exploratory or “hypothesis generating” piece of research

F. Study Design

This is a cohort study design in which all eligible individuals with body mass index recorded are followed-up to death or end of follow-up. The cohort design is suitable for looking at the associations between a single well-defined exposure and a number of different outcomes (in this case different causes of death).

G. Sample Size

We conducted some feasibility counts using the dataset from our previous study looking at BMI and cancer in CPRD.[6] In this dataset, there are over 2 million patients available with BMI data, linkage to the ONS cause of death dataset and with follow-up beyond 5 years from their BMI record (as required in our analysis plan – see section L), and over 129,000 deaths. Each of the broad “second-level” cause-specific mortality outcomes specified in Table 1 below have at least 1000 events observed (ranging up to over 46,000 for cardiovascular/circulatory causes); for the more specific “third-level” outcomes in Table 1 below, the number of outcomes ranges from >500 to >23,000; clearly the precision available will vary widely for different outcomes, but with these numbers we will be able to produce informative estimates of the

relationship between BMI and all planned outcomes. Since we are not specifically testing any primary hypotheses, a formal power calculation is not applicable. The dataset used for these feasibility counts was cut at July 2012; we plan to use updated data from at least January 2016 for our study, making the counts above quite conservative.

H. Data Linkage Required (if applicable)

We require linkage to ONS mortality data, because our outcomes are specific causes of death, and we require death certificate data to ascertain this. We also require linkage to individual-level deprivation data, in order to allow us to adjust for, and investigate effect modification by socioeconomic status.

I. Study Population

We will include all patients with a valid BMI measure and with any up-to-standard CPRD follow-up at least 5 years after this measure was recorded (because we will exclude the first 5 years of follow-up after a BMI record, to guard against reverse causality, see also section L). Only those eligible for linkage to ONS mortality and IMD data will be included. We will restrict to the coverage period for ONS mortality data.

J. Selection of comparison group(s) or controls

There is no separate control group – people at different BMI levels within the main study cohort will be compared.

K. Exposures, Outcomes and Covariates

The main exposure is BMI. BMI will be derived based on weight and height measurements as defined in our previous work.[7]

The outcomes are all-cause mortality, and deaths from grouped and then specific causes, ascertained from linked ONS mortality data, using the underlying cause of death field. We will group cause of death into categories using the Global Burden of Disease categorisation system.[8] This categorises causes of death in a four-level hierarchy. At the highest level is a broad categorisation into communicable diseases, non-communicable diseases, and injuries. At the next level is broad disease groupings such as cancers and cardiovascular/circulatory diseases. At the third level are specific diseases (or injury types) such as lung cancer, or stroke. The fourth level subdivides these further where applicable (e.g. haemorrhagic stroke).

Within this system, we will focus on the associations between BMI and selected outcomes at various levels of the hierarchy which are either common causes of death in the UK,[9] or are *a priori* expected to have important associations with BMI. The outcomes to be considered and the corresponding ICD-10 codes/chapters are shown in Table 1.

Table 1: Cause of death outcomes to be investigated at various levels of the Global Burden of Diseases classification hierarchy

**Cause of death outcomes**	**Corresponding ICD-10 chapters/codes**
**All-cause mortality**	n/a
**Top-level outcomes**	
Communicable diseases	A, B, J00-22
Non-communicable diseases	C through R
Injuries/external	S through Y

**Second-level outcomes**	
Neoplasms	C
Cardiovascular/circulatory	I
Chronic respiratory diseases	J23-99
Liver cirrhosis	K70.3, K71.7, K74.3-6
Digestive other than cirrhosis	K except codes above
Neurological	G
Mental and behavioural	F
Diabetes, urogenital, blood and endocrine	D50-89, E, N
Musculoskeletal	M
**Third-level outcomes (selected)**	
Specific communicable diseases	
Lower respiratory infections	J09-22
Specific neoplasms	
Oesophageal cancer	C15
Stomach cancer	C16
Colorectal cancer	C18-21
Liver cancer	C22
Pancreatic cancer	C25
Lung cancer	C34
Malignant melanoma	C43
Breast cancer	C50
Ovarian cancer	C56
Prostate cancer	C61
Kidney cancer	C64
Bladder cancer	C67
Brain/CNS cancer	C71-72
Haematological malignancy	C81-96
Specific cardiovascular/circulatory	
Hypertensive heart disease	I11
Ischaemic heart disease	I20-I25
Atrial fibrillation/flutter	I48
Heart failure	I50
Cerebrovascular disease	I69
Aortic Aneurysm	I71
Peripheral vascular disease	I73
Specific neurological	
Dementia and Alzheimer's	F00-01, F03, G30
Specific endocrine	
Diabetes mellitus	E10-14
Specific injuries/external	
Suicide/intentional self-harm	X60-84

Our models will also be adjusted for the following covariates, measured at time of BMI record:

- age at BMI record

- calendar year at BMI record
- diabetes (yes/no)
- alcohol status (never, current – low level, current – moderate level, current – high level, current – unknown level, ex)
- smoking status (never, current, ex)
- index of multiple deprivation (categorised into quintiles)
- gender (stratification variable)

The codelists/definitions/algorithms for these variables will be as we used in our previous study (protocol 12_090A2).[6]

L. Data/ Statistical analysis

Time-to event methods will be used. Date of birth (approximated as the mid-point of the year of birth, due to limitations in accessing full date of birth) will be taken as the origin, and individuals will "late-enter"[10] at the latest of: up-to-standard CPRD follow-up, 5 years since first BMI record. The 5-year exclusion period is to avoid reverse causality, whereby a pre-existing disease preceding death affects the BMI. The length of this period will be varied in sensitivity analyses (see below). In the analysis we will estimate both the cause-specific associations between BMI and individual cause of death outcomes, and cumulative incidences for each outcome in the presence of the competing risks of death from other causes, as follows:

1. Cause specific regression modelling: Cox regression models for all-cause mortality and then for each cause-specific death outcome (Table 1) will be fitted on an underlying age timescale, based on the cause-specific hazard (censoring competing causes), stratified by gender and adjusted for confounders (section K). BMI will be fitted initially in WHO categories (<18.5 = underweight, 18.5-24.9=healthy weight, 25.0-29.9=overweight, ≥30=obese) and then as a natural cubic spline to visualise non-linearity. By fitting interaction terms we will investigate effect modification of the BMI/outcome relationships by age, sex, smoking status, alcohol use, ethnicity (dependent on completeness) and socioeconomic status (index of multiple deprivation quintile).

Should there be evidence for non-linearity in our spline models then we will estimate simplified piecewise linear approximations to the cubic spline curves to quantify this. We will do this by estimating best-fitting thresholds/turning points (fitting all possible thresholds and choosing the model that maximises the likelihood).

2. Cumulative incidences: Cumulative incidences for each broad "second-level" cause of death category (Table 1) will be calculated within WHO BMI categories (see above), using competing risks methods.[11] This will allow us to see how the overall cumulative incidence curve for death by age, is constituted in terms of individual causes of death, and to describe differences in the distribution of causes of death by BMI group.

Sensitivity/secondary analyses

1. *Exclusion of person-time after BMI measure:* We will vary the exclusion period for follow-up after a BMI measure to 1, 5, and 10 years to help exclude/identify reverse causality

2. *Incidence based mortality analysis*: for specific ("third-level") causes of deaths with suspected causal links to BMI, we will conduct a sensitivity analysis that includes only deaths among those whose disease was known to be incident during follow-up (defined as first coded in clinical data at least 12 months after current registration date) to help identify reverse causality.

3. *Restriction to those with "administratively recorded" BMI:* We will restrict to people with a BMI recorded within 12 months of start of CPRD follow-up, in order to restrict to people in whom BMI

was more likely to be recorded for administrative rather than health reasons (and thus to exclude selection bias due to BMI missingness)

4. *Restriction to later calendar time:* We will successively exclude early periods of CPRD data during which BMI completeness was low, in order to exclude selection bias due to BMI missingness.

M. Plan for addressing confounding

As per sections K/L we will use an underlying age timescale in our model to provide close control for age; we will stratify by gender; and we will adjust for variables measured at or close to the time of the BMI record (age at BMI, calendar year, diabetes status, alcohol status, smoking status and deprivation), to reduce confounding. The modelling will be carried out both in the full study population and in never-smokers only, to exclude residual confounding by smoking dose (which we observed in our previous study on BMI and cancer[6]).

N. Plan for addressing missing data

A substantial proportion of patients in CPRD will have no BMI recorded. We will exclude such patients; this is known as a "complete case" or "complete records analysis". Such an approach is unbiased in a regression analysis providing the probability of being a complete case is conditionally independent of the outcome (in this case cause-specific deaths).[12] As we argued in our previous study,[6] we consider this to be a preferable assumption than "missing at random" (used in multiple imputation) which would assume that missingness of BMI to be unrelated to BMI itself (unlikely in routine primary care records).[13]

We will conduct a number of sensitivity analyses to exclude the possibility of our "complete case" approach leading to bias (see also section L): (i) restricting the analysis to people with a BMI recorded within 12 months of GP registration (more likely to be recorded for administrative rather than health reasons); (ii) successively restricting the analysis to more recent calendar periods, when we have shown that BMI completeness increased substantially.[7]

There may also be individuals with missing smoking and alcohol, whom we will similarly exclude (based on the same logic), but from previous experience, we think the large majority of people with a BMI record will also have smoking and alcohol status records.

O. Limitations of the study design, data sources and analytical methods

- There are limitations to the quality of death certificate data. Cause of death is not always known with certainty and a presumptive cause may be entered on the death certificate, leading to misclassification of outcomes in our analysis. Furthermore, if physicians' choices of assumed cause of death are related to patient BMI (e.g. a tendency to assume cardiovascular-related deaths among the obese), then this could bias our estimated BMI-cause-of-death associations. There may also be delays in death registrations, particularly for causes of death such as injuries/external. These limitations will be discussed in our outputs.
- There may be selection bias due to excluding those with no BMI record. We will discuss the assumption needed for our analysis to be unbiased. We will conduct a number of sensitivity analyses to try and exclude this. See also section N.

P. Patient or user group involvement (if applicable)

None.

Q. **Plans for disseminating and communicating study results, including the presence or absence of any restrictions on the extent and timing of publication**

The protocol is expected to lead to at least one peer-reviewed paper in a scientific journal describing the main findings. We may also present our data at appropriate conferences.

R. **References**

1. Prospective Studies C, Whitlock G, Lewington S, et al. Body-mass index and cause-specific mortality in 900 000 adults: collaborative analyses of 57 prospective studies. *Lancet* 2009; **373**(9669): 1083-96.

2. Berrington de Gonzalez A, Hartge P, Cerhan JR, et al. Body-mass index and mortality among 1.46 million white adults. *N Engl J Med* 2010; **363**(23): 2211-9.

3. Flegal KM, Kit BK, Orpana H, Graubard BI. Association of all-cause mortality with overweight and obesity using standard body mass index categories: a systematic review and meta-analysis. *JAMA* 2013; **309**(1): 71-82.

4. Nordestgaard BG, Palmer TM, Benn M, et al. The effect of elevated body mass index on ischemic heart disease risk: causal estimates from a Mendelian randomisation approach. *PLoS Med* 2012; **9**(5): e1001212.

5. Parr CL, Batty GD, Lam TH, et al. Body-mass index and cancer mortality in the Asia-Pacific Cohort Studies Collaboration: pooled analyses of 424,519 participants. *Lancet Oncol* 2010; **11**(8): 741-52.

6. Bhaskaran K, Douglas I, Forbes H, dos-Santos-Silva I, Leon DA, Smeeth L. Body-mass index and risk of 22 specific cancers: a population-based cohort study of 5.24 million UK adults. *Lancet* 2014; **384**(9945): 755-65.

7. Bhaskaran K, Forbes HJ, Douglas I, Leon DA, Smeeth L. Representativeness and optimal use of body mass index (BMI) in the UK Clinical Practice Research Datalink (CPRD). *BMJ open* 2013; **3**(9): e003389.

8. GBD 2013 Mortality and Causes of Death Collaborators. Global, regional, and national age-sex specific all-cause and cause-specific mortality for 240 causes of death, 1990-2013: a systematic analysis for the Global Burden of Disease Study 2013. *Lancet* 2015; **385**(9963): 117-71.

9. Newton JN, Briggs AD, Murray CJ, et al. Changes in health in England, with analysis by English regions and areas of deprivation, 1990-2013: a systematic analysis for the Global Burden of Disease Study 2013. *Lancet* 2015; **386**(10010): 2257-74.

10. Collett D. Modelling survival data in medical research: CRC press; 2015.

11. Andersen PK, Geskus RB, de Witte T, Putter H. Competing risks in epidemiology: possibilities and pitfalls. *Int J Epidemiol* 2012; **41**(3): 861-70.

12. White IR, Carlin JB. Bias and efficiency of multiple imputation compared with complete-case analysis for missing covariate values. *Stat Med* 2010; **29**(28): 2920-31.

13. Bhaskaran K, Smeeth L. What is the difference between missing completely at random and missing at random? *Int J Epidemiol* 2014; **43**(4): 1336-9.

Known deviations from approved protocol, and reasons

1) Uterus cancer mortality was added as an outcome because of the strong known link between BMI and uterus cancer risk
2) Deaths due to external causes were explored in more detail than originally planned in the protocol. The following "second-level" outcomes were added: accidental death (transport-related – ICD-10 chapter V), accidental death (not transport-related – ICD-10 chapter W and X00-59), and self-harm/interpersonal violence (ICD-10 X60-Y09); deaths due to falls (ICD-10 W00-19) was also added as an additional third-level outcome. These outcomes were added in order to further explore and characterise the observed inverse association between BMI and deaths from external causes.
3) We divided the "second-level" urogenital, blood and endocrine outcome into two categories - blood and endocrine; and urogenital. This is a departure from the Global Burden of Diseases classification on which our outcomes were based but we felt on discussion that combining these disparate outcomes into one was difficult to clinically justify.
4) The ICD-10 list for cerebrovascular diseases was corrected to include all of "I60-69"
5) Deprivation data (index of multiple deprivation) was obtained in twentiles rather than quintiles, to give greater granularity to the control for deprivation; it was then fitted using a non-linear spline, rather than a categorical variable, again to improve the degree of control for deprivation.
6) An additional analysis was added to calculate expected lifespan from age 40 by BMI category, accounting for key covariates and interactions, to provide information on absolute effects of BMI, and to help put relative risks into context.

Part 4 - Systematic review of studies investigating links between BMI and cause-specific mortality outcomes –methods and results

Methods for systematic review

Databases searched: Pubmed

Search string: title search for ("body mass index" OR bmi OR obes*OR overweight) AND (mortality OR death), filtered to articles with abstracts from the last 10 years (2007-2017)

Inclusion criteria: Articles were included if they provided estimates of association between BMI (treated as a continuous or multi-categorical (i.e. more than binary) variable), and one or more cause-specific mortality outcomes in adults.

Exclusion criteria: The following were excluded: studies of children, studies of patients with specific prior diseases (e.g. diabetes), intervention studies, studies of weight change, commentary pieces.

Extraction of study characteristics: The following were extracted from each included study where information was available: study design, setting population, number of people in study, average follow-up, strategy for dealing with reverse causality (e.g. exclusion of early person-time, exclusion of people with prior disease), outcomes considered.

Extraction of study results: Relative risk estimates were extracted directly where presented numerically. Where both linear and non-linear (e.g. categorical, or fitted curve) estimates of BMI-outcome associations were presented in a study, we extracted for presentation the results that allowed for non-linearity. Where results were only presented graphically, these were extracted using DigitizeIt software (www.digitizeit.de) – at least 4 data points were extracted across the range of BMI, but more were extracted if this was considered necessary to adequately characterise the curve.

Processing of study results: Descriptive characteristics of the included studies are presented in table form. Results of all studies are summarised graphically. Studies in predominantly Asian settings were presented separately to those from Europe/North America/Australia because of different patterns of diseases and different BMI distributions in Asian settings. Results are organised into cardiovascular disease mortality outcomes, cancer mortality outcomes, and other mortality outcomes. Where BMI-outcome associations in the original study were estimated in categories, these are presented as scatter plots, with estimates plotted at the mid-point of the relevant BMI category. Where BMI-outcome associations were estimated as linear effects in the original study (e.g. HR per 5kg/m^2) these are presented as straight line plots with the reference point at 21.75kg/m^2 (the mid-point of the "healthy weight" category); though where two-line models were fitted, the reference was instead placed at the join-point of the two lines. Where BMI-outcome associations were estimated as non-linear curves in the original study, these are presented as scatter plots, with points plotted at the same values of BMI as were used for the graphical extraction of the data (see above). Finally, we excluded from the graphical presentation of results studies where the categories of BMI were unclear in the original study (e.g. categorised into quintiles, but quintiles boundaries not given), or where linear effects were presented but not well-defined (e.g. per standard deviation, but standard deviation not given). Results from studies including data from European/North American/Australian settings are summarised separately to those from Asian settings, because they would be expected to be more comparable to our UK-based data analysis study.

Table S4.1: Description of studies included in the systematic review

Author and year	Design/Setting	Study population	Number included	Av follow-up (yrs)	Strategy for dealing with reverse causality	Mortality outcomes (grouped as CVD, cancer, respiratory, other)
Adabag 2015[1]	Cohort: Atherosclerosis Risk in Communities study, 4 US communities	African American and white men and women age 45-64	14941	12.6	Not mentioned	CVD: Sudden cardiac death
Arnlov 2009[2]	Cohort: Uppsala Longitudinal Study of Adult Men	Men aged 50 years without diabetes	1758	30	Excluded if prior hospitalisation for CVD	CVD: CVD composite (CVD death or hospitalisation for MI, stroke, heart failure)
Aune 2016[3]	Systematic review and meta-analysis:	23 prospective studies	647388	varying per constituent study	Not mentioned	CVD: Heart failure
Batty 2008[4]	Cohort: Whitehall Study, UK	Male government employees aged 40-69	18863		First 10y excluded in a secondary analysis	OTHER: Liver disease mortality
Batty 2009[5]	Pooled cohort study: Asia Pacific Cohort Study Collaboration (Pooled IPD from 44 cohort studies)	Men and women aged 20+ years	405799	4	Not mentioned	CANCER: Liver cancer
Bessanova 2011[6]	Cohort: California Teachers Study	Female California public school teachers	115433	11.5	Excluded if prevalent disease at baseline	CANCER: Any cancer, breast cancer<br>CVD: CVD mortality<br>RESPIRATORY: Respiratory disease mortality
Bethea 2014[7]	Pooled cohort study: Pooled IPD from 7 US cohort studies	African American men and women	239597	11.6	First year excluded; only those followed up for 5+ years included in main analysis	CANCER: Pancreatic cancer
Bombelli 2013[8]	Cohort: PAMELA cohort study, Monza, Italy	Male and female residents of Monza, Italy, age 25-74	2005	Not stated	Not mentioned	CVD: CVD mortality
Borrell 2014[9]	Cohort: US NHANES III study linked to national death registration	US adults aged 18+	16868	13.2	Not mentioned	CVD: CVD mortality

Cao 2011[10]	Systematic review and meta-analysis: 6 cohort studies	6 cohort studies	1263483	varying per constituent study	Not mentioned	CANCER: Prostate cancer
Carlsson 2011[11]	Cohort: Swedish Twin Registry	Same sex twin pairs born 1886-1958	44258	25.7	First 2y excluded	CVD: CVD mortality and CHD mortality
Chen Y 2013[12]	Pooled cohort study: Pooled IPD from >20 Asian cohort studies	Men and women from Japan, China, Korea, India, Taiwan, Bangladesh and Singapore	1124897	9.7	First 3y excluded	CVD: CVD, CHD, Stroke
Chen Z 2012[13]	Cohort: Population-based Chinese cohort study in men	Men aged 40-79 at baseline from 45 areas across China	142214	Not stated	Excluded if prevalent disease at baseline, minimum 5y follow-up required	CANCER: Cancer, Lung cancer, liver cancer, stomach cancer, upper aerodigestive cancer<br>CVD: CVD, CHD, Stroke<br>RESPIRATORY: Respiratory disease, COPD<br>OTHER: Diabetes, Kidney disease, Liver disease,
Chu 2011[14]	Cohort: People undergoing health exam in a private screening centre in Taiwan	Men and women aged 19-98 in Taiwan	383956	7.2	Excluded if prior cancer, First 2y excluded	CANCER: NHL, leukaemia
Cohen 2014[15]	Pooled cohort study: Pooled IPD from 7 US cohort studies	African American men and women	239526	11.7	First year excluded	CANCER: Cancer<br>CVD: CVD
Crump 2017[16]	Cohort: Historical cohort of military conscripts linked to national registry data	Male military conscripts in Sweden aged 18 at baseline in 1969-1997	1547478	28.2	Not mentioned	CVD: CVD
Czernichow 2011[17]	Pooled cohort study: Pooled IPD from 9 UK population-based cross-sectional surveys linked to mortality data	Men and women in the UK	82864	8.2	Not mentioned	CVD: CVD

de Hollander 2012[18]	Cohort study: Participants in the Survey in Europe on Nutrition and the Elderly (SENECA) study, based in European Towns	Adults aged 70-75	1970	Not stated	Not mentioned	CANCER: Cancer<br>CVD: CVD<br>RESPIRATORY: Respiratory disease
Dehal 2012[19]	Cohort: People from NHANES 1 epidemiology f-up study (NHEFS), US	US adults aged 25-74; underweight excluded	7016	17.0	Excluded if prior cancer	CANCER: Colorectal, lung, breast, prostate cancers
Dudina 2011[20]	Pooled cohort study: 12 European cohort studies in 10 countries - 95% population-based, 5% occupational	Men and women across Europe	186308	Not stated	Sensitivity analysis excluding first 2 and 5y	CVD: CVD
Eranti 2015[21]	Cohort: Finnish Social Insurance Institution Coronary Heart Disease Study	Men and women aged 30-59 in 35 areas of Finland	10957	Not stated	Not mentioned	CVD: Sudden cardiac death
Faeh 2011[22]	Cohort: Swiss MONICA cohort study	Men and women aged 25-74 in Switzerland	9853	18.6	Not mentioned	CANCER: Cancer<br>CVD: CVD
Fowke 2015[23]	Pooled cohort study: Asia Cohort Consortium	18 prospective cohorts from 6 countries in East and South Asia	522736	9.2	Adjusted for prevalent cancer at baseline	CANCER: Prostate cancer
Funada 2008[24]	Cohort: Ohsaki Study, Japan	Men and women aged 40-79 in Japan	43916	6.2	Excluded if prior cancer, ischaemic heart disease or stroke at baseline	CVD : CVD, stroke, IHD
Genkinger 2015[25]	Pooled cohort study: National Cancer Institute (US) BMI and Mortality Cohort Consortium	20 prospective cohorts of US men and women aged 18 to 85	1096492	Not stated	Excluded if prior cancer; 1y minimum follow-up required	CANCER: Pancreatic cancer
Ghaem Maralani 2013[26]	Cohort: Blue Mountains Eye Study, Australia	White male and female permanent residents aged 49+ of 2 urban postcode areas of New South Wales, Australia	2216	14.7	Adjusted for pre-existing disease (also examined as an effect modifier)	CANCER: CA<br>CVD: CVD

Global BMI Mortality Collaboration 2016[27]	Systematic review and meta-analysis: 239 prospective cohort studies	239 prospective cohort studies from Asia, Australia, New Zealand and Europe; restricted to never-smokers	3.9 million	13.7	First 5y excluded, excluded if prior chronic disease diagnoses	CANCER: Cancer<br>CVD: CHD, stroke<br>RESPIRATORY: Respiratory disease
Gray 2012[28]	Cohort: Harvard Alumni Health Study	Male Harvard alumni with a physical examination between 1914 and 1952 (at mean age of 18.4)	19593	56.5	Min 10-y f-up required; sensitivity analyses excluding first 3y	CANCER: 17 site specific cancers
Han 2014[29]	Cohort: Atherosclerosis Risk in Communities study, 4 US communities	Men and women age 45-64	13901	Not stated	First 3y excluded	CANCER: Any cancer, obesity related w/o breast, postmenopausal breast cancer, colorectal, endometrial
Hart 2011[30]	Cohort: Renfrew and Paisley Study, Scotland	Female never-smokers aged 45-64 at recruitment, from community based cohort study in West Central Scotland	3613	22.7	Not mentioned	CANCER: Cancer, breast cancer<br>CVD: CVD, chd, stroke<br>RESPIRATORY: Respiratory disease
Hong 2007[31]	Cohort: Kangwha Cohort Study, Korea	Male residents of 10 administrative districts of Korea, aged 55 and over at baseline	2608	Not stated	Sensitivity analysis excluding first 5y	CVD: CVD, cerebrovascular disease
Hong 2015[32]	Cohort: Kangwha Cohort Study, Korea	Residents of rural communities, aged 55+	6166	12.5	Adjusted for pre-existing chronic diseases; sensitivity analysis excluding first 5y and those with prior chronic diseases or cancer	CANCER: Cancer<br>CVD: CVD<br>RESPIRATORY: Respiratory
Hong 2016[33]	Cohort: Korean Veterans Health Study	Male Korean veterans	113478	6.4	First 2y excluded, and excluded if prior cancers	CANCER: Cancer, overall and site-specific
Hwang 2011[34]	Cohort: Six communities in Taiwan	Men and women aged 20-65 at baseline	6603	24	First 3y excluded, and excluded if prior stroke or cancer	CANCER: Cancer<br>CVD: CVD<br>DIAB: Diabetes
Jia 2016[35]	Cohort: Swedish Multigeneration Register linked to	Men with conscription records aged 18-20 in Sweden	743398	35.9	Not mentioned	OTHER: All unintentional injury, road accidents, poisoning, falls, fire, drowning

	national registries/death data					
Kee 2017[36]	Cohort: National Health and Morbidity Survey, linked to death registrations, Malaysia	Men and women aged 18+	32844	4.8	First 2y excluded	CVD: CVD
Kim 2015[37]	Cohort: National Health Insurance Service Database, Korea	Men and women in Korea	153484	7.91	Excluded if prior CVD or cancer	CANCER: Cancer<br>CVD: CVD
Kivimaki 2008[38]	Cohort: Whitehall Study	Working men in UK civil service, aged 40-69	18860	35	Sensitivity analysis excluding first 5y, secondary analysis in healthy sub-cohort (n=7865)	CANCER: Cancer<br>CVD: CVD<br>RESPIRATORY: Respiratory disease
Klenk 2009[39]	Cohort: Vorarlberg Health Monitoring and Promotion Program (VHM&PP), Austria	Men and women in an Austrian province (population-based)	184697	15.1	Sensitivity analyses excluding first 1y, 3y	CANCER: Cancer<br>CVD: CVD<br>RESPIRATORY: Respiratory disease
Korkeila 2009[40]	Cohort: Finnish Twin Cohort	Same sex twin pairs born 1886-1958, aged 24-60 in 1981	15424	Not stated	Not mentioned	CVD: CVD, CHD<br>OTHER: Violent causes (accidental plus suicide)
Leitzmann 2011[41]	Cohort: NIH-AARP Diet and Health Study	AARO members aged 50-71 in 6 US states/2 metropolitan areas; uw excluded	225712	8.7	Not mentioned	CANCER: Cancer, Lung cancer<br>CVD: CVD, CHD<br>RESPIRATORY: Chronic respiratory disease<br>OTHER: Injuries
Li 2013[42]	Cohort: Japan Collaborative Cohort study	Men and women aged 40-79 in Japan	72473	19	First 10y excluded	CANCER: Liver cancer
Lin 2013[43]	Pooled cohort study: Asia Cohort Consortium	16 pspective cohorts from 6 countries in East and South Asia	883529	9.7	Excluded if prior cancer	CANCER: Pancreatic cancer
Ma 2011[44]	Cohort: US National Health Interview Survey linked to mortality data	Men and women aged 18-39 at baseline	112328	16	Sensitivity analyses excluding first 5y and restricting to healthy participants at baseline	CANCER: Cancer<br>CVD: CVD
Mirbolouk 2015[45]	Cohort study: Tehran Lipid and Glucose study	People aged 65+ in district 13 of Tehran	1199	9.74	Not mentioned	CVD: CVD

Nakade 2015[46]	Cohort: AGES Cohort study, Japan	Men and women aged 65+ and physically/cognitively independent at baseline	14931	3.8	Deaths within first 1y excluded	CANCER: Cancer<br>CVD: CVD<br>RESPIRATORY: Respiratory disease
Odegaard 2010[47]	Cohort: Singapore Chinese Health Study	Chinese male and female never-smokers aged 45-74	30538	12.7	Excluded if prior cancer, prevalent CVD, diabetes, or respiratory disease; mortality within first 5y excluded	CANCER: Cancer<br>CVD: CVD
Park 2014[48]	Pooled cohort study: 10 US cohort studies	10 prospective cohorts with data on Asian American adults in the US	20672	Not stated	Excluded if prior cancer or heart disease	CANCER: Cancer<br>CVD: CVD
Parr 2010[49]	Pooled cohort study: Asia Pacific Cohort Study Collaboration (Pooled IPD from 40 cohort studies)	Men and women aged 20+ years	424519	6.7	First 3y excluded	CANCER: Cancer, multiple specific cancers
Paulsen 2017[50]	Cohort: the HUNT study, Norway	Men and women aged 20+ participating in population-based survey from one county in Norway	64027	14.8	Not mentioned	OTHER: Bloodstream infection
Pednekar 2008[51]	Cohort: Cohort study in Mumbai	Men and women registered on voters list and aged 35+, excluding affluent housing, in Mumbai, India	~100,000	Not stated	First 2y excluded	CANCER: Cancer<br>CVD: CVD<br>RESPIRATORY: Respiratory<br>OTHER: Tuberculosis, digestive system
Prospective Studies Collaboration 2012[52]	Pooled cohort study: 57 prospective studies mostly in western Europe and N America	57 prostpective cohort studies; 92% of participants from Europe, US, Australia; 8% from Japan	894576	8	First 5y excluded	CANCER: Cancer, multiple specific cancers<br>CVD: CVD, aortic aneurysm, atherosclerosis, CHD, heart failure, pulmonary embolism, rheumatic heart disease, sudden cardiac death, stroke<br>RESPIRATORY: Respiratory, COPD, pneumonia

						OTHER Respiratory tuberculosis, diabetes, kidney disease, liver disease, cirrhosis, external causes
Reeves 2007[53]	Cohort: UK cohort study	Women aged 50-64 in the UK	1.2 million	7.0	Excluded if prior cancer	CANCER: Cancer and several site-specific cancers
Sasazuki 2011[54]	Pooled cohort study: 7 cohort studies in Japan	Men and women from 7 population-based cohort studies in Japan	353422	12.5	First 5y excluded	CANCER: Cancer<br>CVD: Heart disease (I20-52), cerebrovascular disease
Sauvaget 2008[55]	Cohort: Trivandrum Oral Cancer Study - cluster RCT in Kerala, India	Men and women aged 35+ in Kerala (never-smoker results shown here)	75868	8	First 3y excluded	CANCER: Cancer<br>CVD: CVD, CHD, Stroke<br>RESPIRATORY: Respiratory disease<br>OTHER: Diabetes
Song 2012[56]	Pooled cohort study: 33 prospective studies in 11 European countries	Men and women aged 24+ in Europe, never-smoker results shown here	135745	16.8	Sensitivity analysis with first 5y excluded	CANCER: Cancer<br>CVD: CVD
Staiano 2012[57]	Cohort: Canadian Health Survey	National survey in Canada sampled from health insurance registries	8061	13	Not mentioned	CANCER: Cancer, prostate, breast, colon, lung cancers<br>CVD: CVD, IHD, Stroke, CHF/other
Taghizadeh 2015[58]	Cohort: Vlagtwedde-Vlaardingen cohort, Netherlands	Caucasian individuals of Dutch descent, aged 20-65 at baseline	8465	Not stated	Not mentioned	CANCER: Cancer, lung, colorectal, prostate, breast cancers
Takata 2013[59]	Cohort: Cohort study in Japan	Elderly people (80+) in urban, surburban and rural areas of Japan's Fukuoka Prefecture	675	Not stated	Not mentioned	CANCER: Cancer<br>CVD: CVD<br>RESPIRATORY: Respiratory disease
Wang 2016[60]	Cohort: Linxian Nutrition Intervention Trial study population in China	Residents of a rural county in N China aged 40-69 with no history of cancer or debilitating disease	29446	21.2	Excluded if prior cancer	CANCER: Oesophogeal squamous cell carcinoma

Yang 2009[61]	Cohort: Population-based Chinese cohort study in men	Men aged 40-79 at baseline from 45 areas across China	217180	~15	First 3y excluded	CANCER: Lung cancer
Yang 2010[62]	Cohort: Population-based Chinese cohort study in men	Men aged 40-79 at baseline from 45 areas across China	221194	~15	Excluded if prior respiratory disease or impaired lung function; sensitivity analysis excluding first 5y	RESPIRATORY: COPD
Yi 2009[63]	Cohort: Kangwha Cohort Study, Korea	Female residents of 10 districts in S Korea, , aged 55+	3321	17.8	Not mentioned	CVD: Stroke
Yun 2010[64]	Cohort: Insurance data from government workers and teachers in Korea	Middle aged government workers/teachers in Korea, aged 30-64	473358	Not stated	Excluded if prior cancer or CVD	CVD: CVD
Zajacova 2012[65]	Cohort: Participants in the National Health Interview Survey (nationally representative interview survey)	Non-Hispanic white men and women aged 50-80 with BMI between 15 and 45	266302	Not stated	Sensitivity analysis adjusting for self-rated health and bed-disability days was done in a sensitivity analysis	CANCER: Cancer<br>CVD: CVD<br>RESPIRATORY: Respiratory<br>Other: Diabetes
Zheng 2011[66]	Pooled cohort study: Asia Cohort Consortium	Participants in 19 cohorts from East and South Asia; (never-smoker results extracted)	1.14 million	9.2	First 3y excluded; excluded if prior disease	CANCER: Cancer<br>CVD: CVD<br>RESPIRATORY: Respiratory disease
Zhou 2008[67]	Cohort: 45 disease surveillance points in 45 areas across China	Men aged 40+ in 45 areas across China, intended to be nationally representative; (never-smoker results extracted)	211946	10	First 3y excluded; excluded if prior stroke or heart disease	CVD: Stroke

Figure S4.1: Flow chart of systematic review search and inclusions/exclusions

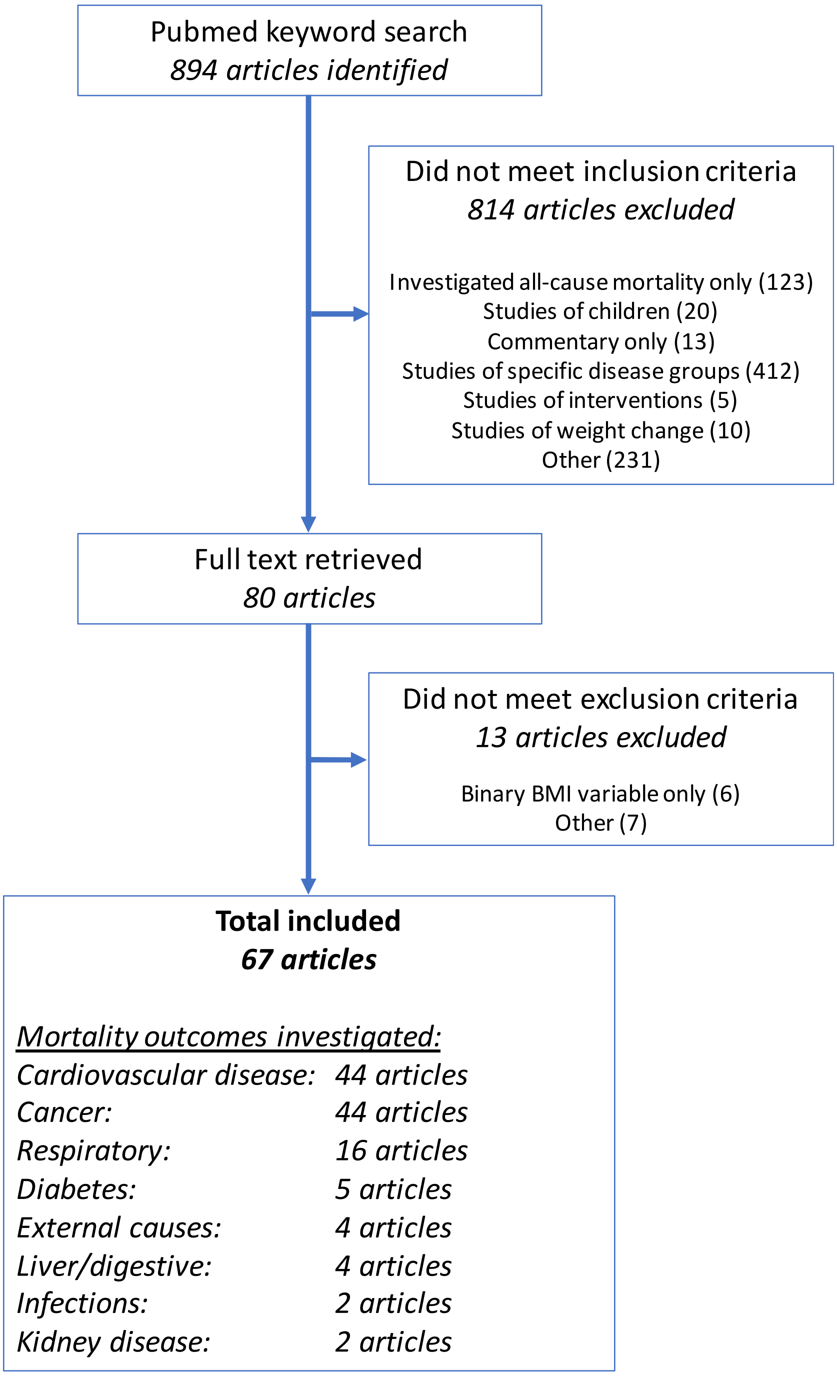

Figure S4.2: Relative risk estimates for association between BMI and cardiovascular disease mortality from studies in European, North American, Australian and trans-continental settings

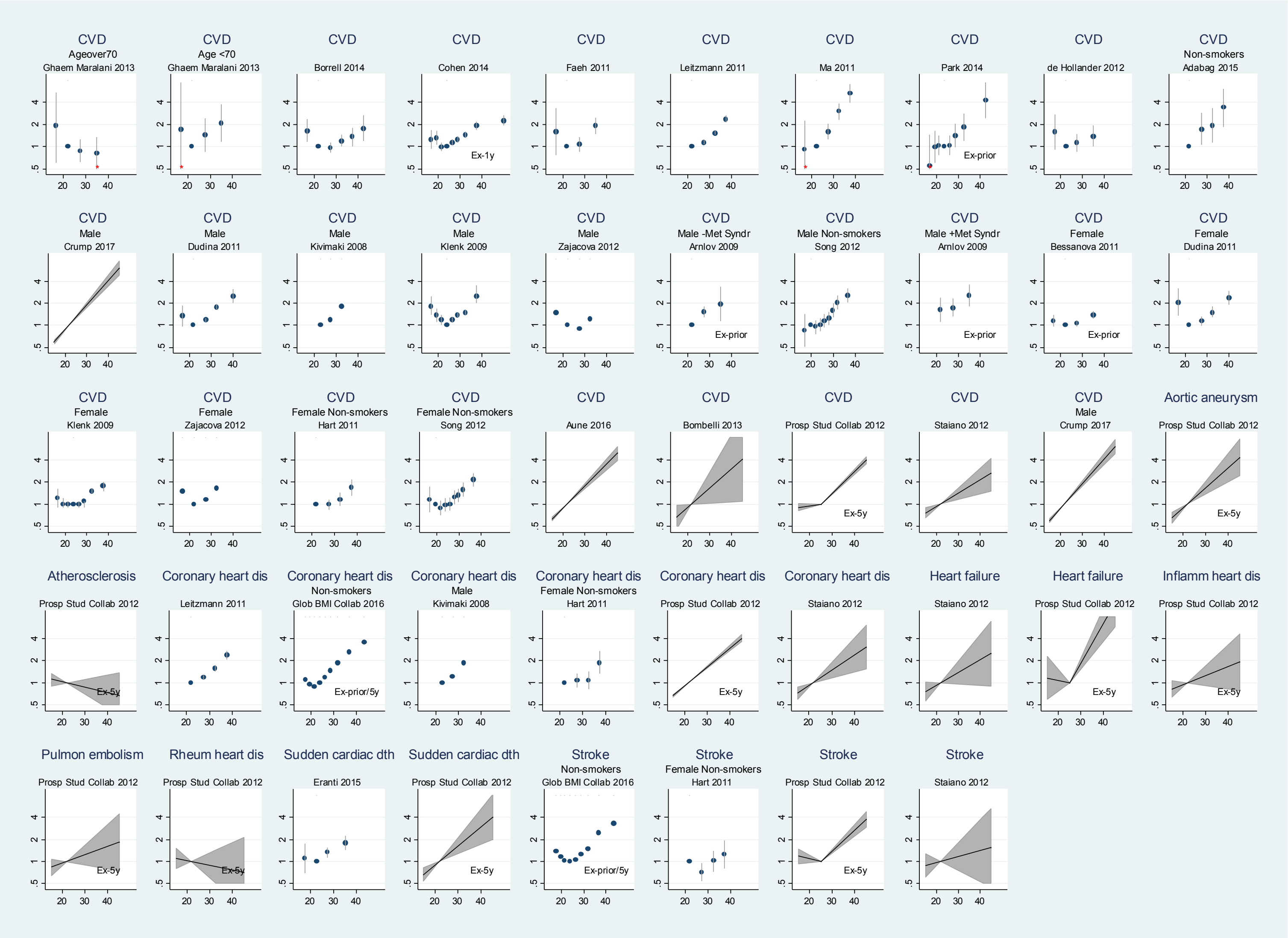

Notes: x-axis is BMI in kg/m², y-axis is relative risk; details of studies in webappendix table W2.1. "Ex-prior" indicates the authors excluded prior/prevalent disease at baseline. "Ex-1y", "Ex-5y" etc indicates that the authors excluded early follow-up for the stated number of years.

Figure S4.3: Relative risk estimates of association between BMI and cancers mortality outcomes from studies in European, North American, Australian and trans-continental settings

(a) Mortality from "any cancer", colorectal, lung, breast, prostate cancers

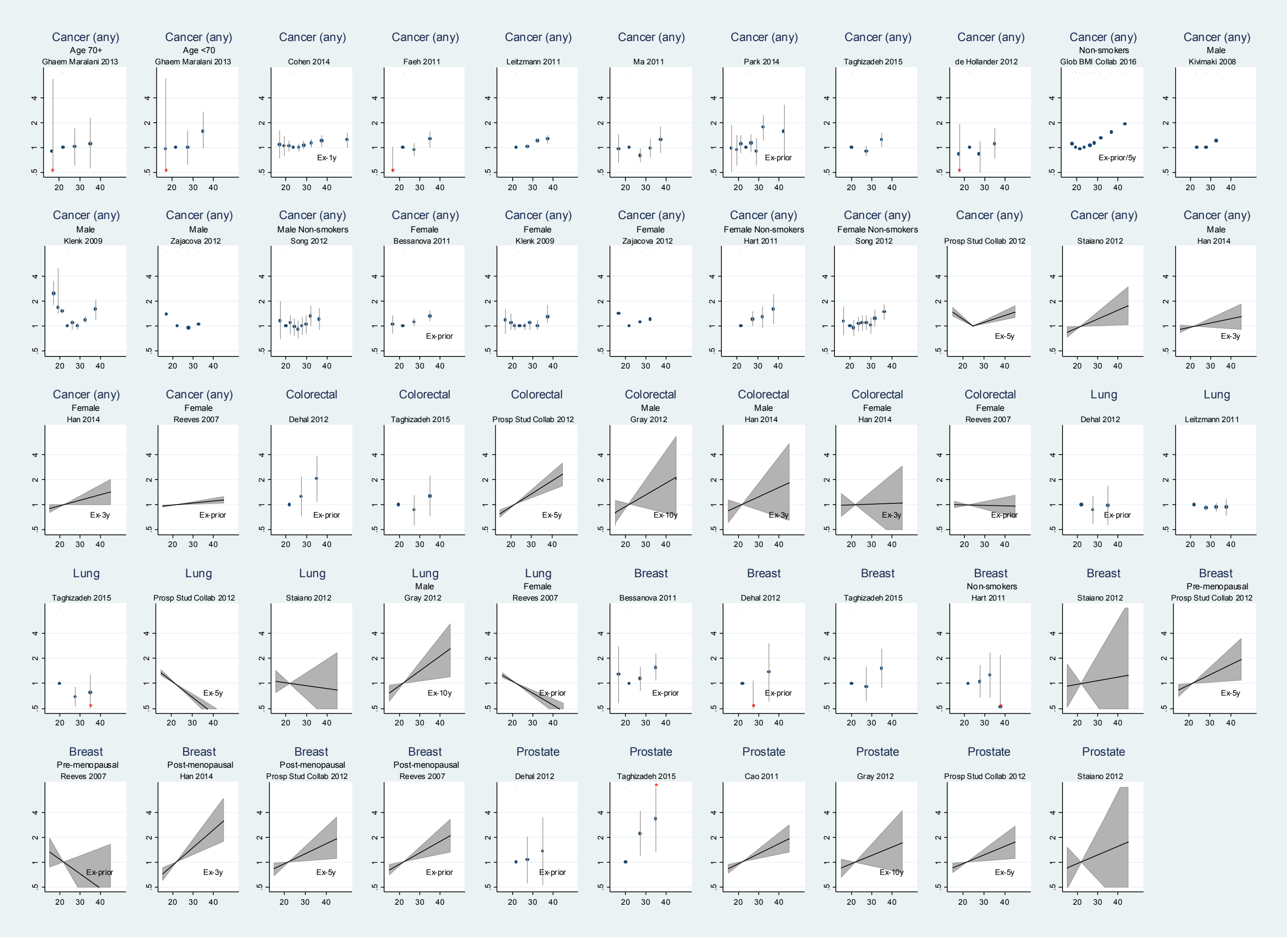

Note: x-axis is BMI in kg/m², y-axis is relative risk; details of studies in webappendix table W2.1; * indicates estimate or CI are beyond the range of the y-axis. "Ex-prior" indicates the authors excluded prior/prevalent disease at baseline. "Ex-1y", "Ex-5y" etc indicates that the authors excluded early follow-up for the stated number of years.

(b) Mortality from less common cancers

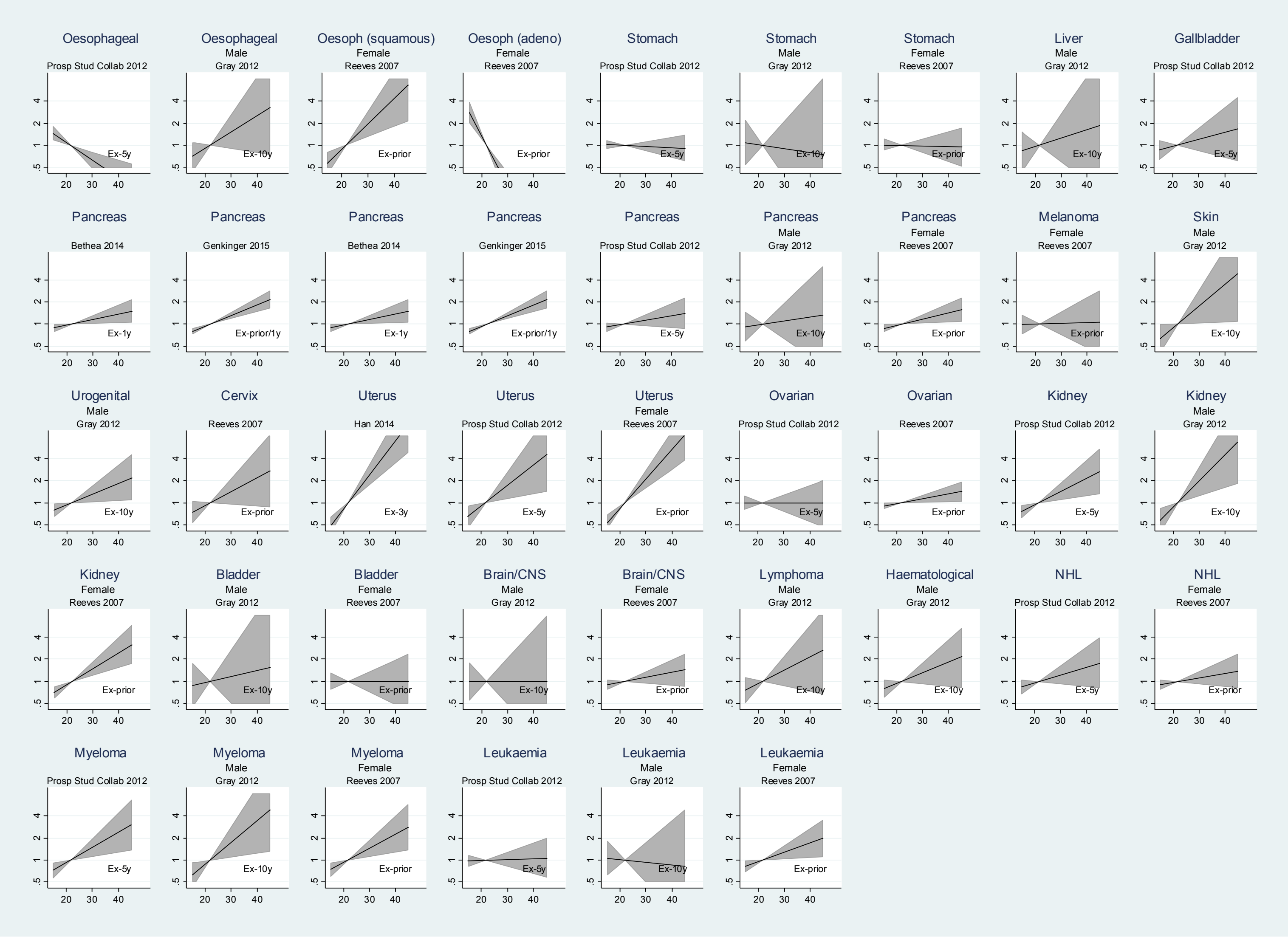

Note: x-axis is BMI in kg/m^2, y-axis is relative risk; details of studies in webappendix table W2.1. “Ex-prior” indicates the authors excluded prior/prevalent disease at baseline. “Ex-1y”, “Ex-5y” etc indicates that the authors excluded early follow-up for the stated number of years.

Figure S4.4: Relative risk estimates of association between BMI and mortality outcomes other than CVD/cancer from studies in Europe, North American and trans-continental settings

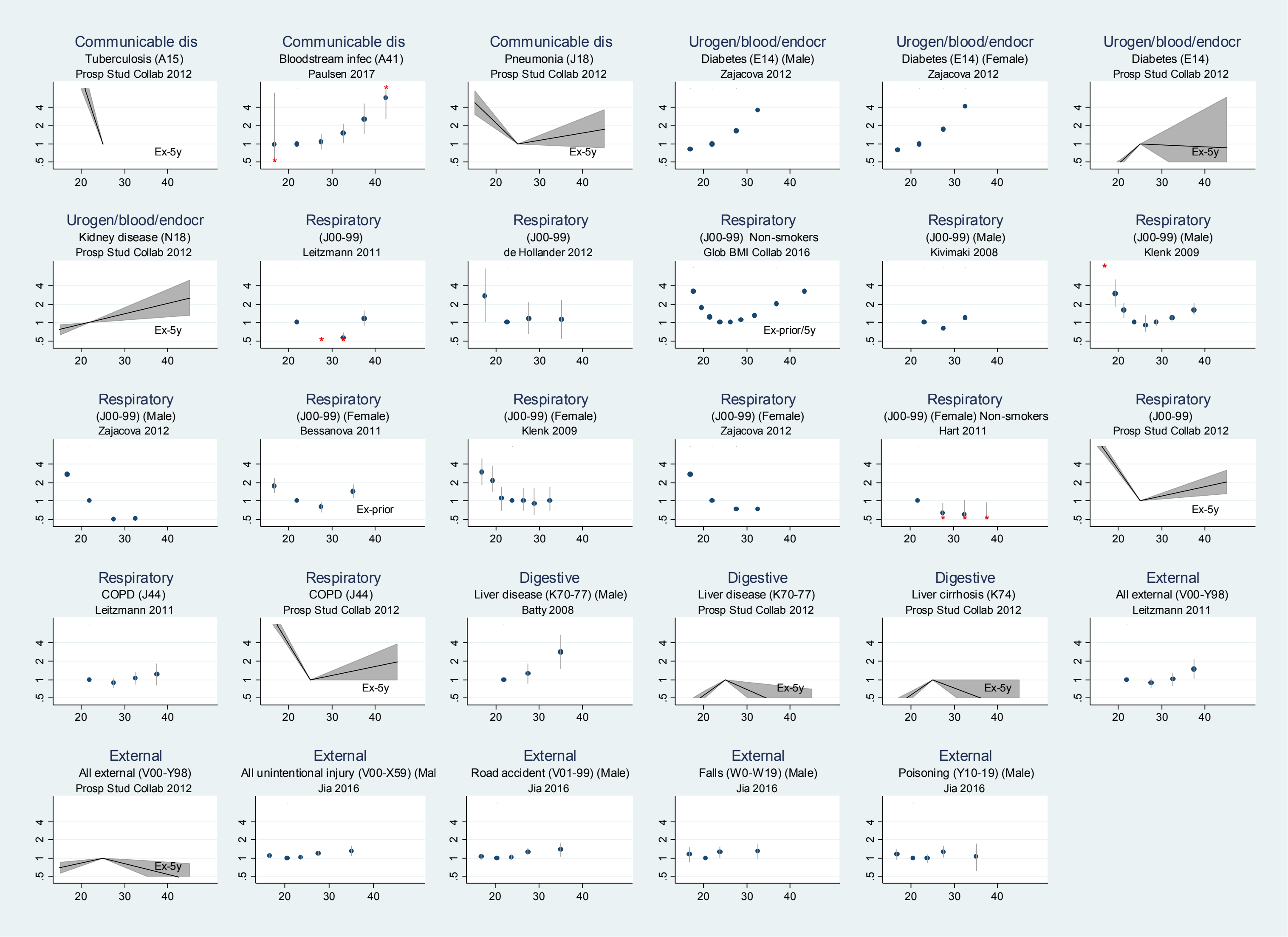

Note: x-axis is BMI in kg/m^2, y-axis is relative risk; details of studies in webappendix table W2.1; * indicates estimate or CI are beyond the range of the y-axis. "Ex-prior" indicates the authors excluded prior/prevalent disease at baseline. "Ex-1y", "Ex-5y" etc indicates that the authors excluded early follow-up for the stated number of years.

Figure S4.5: Relative risk estimates for association between BMI and cardiovascular disease mortality from studies in Asian settings

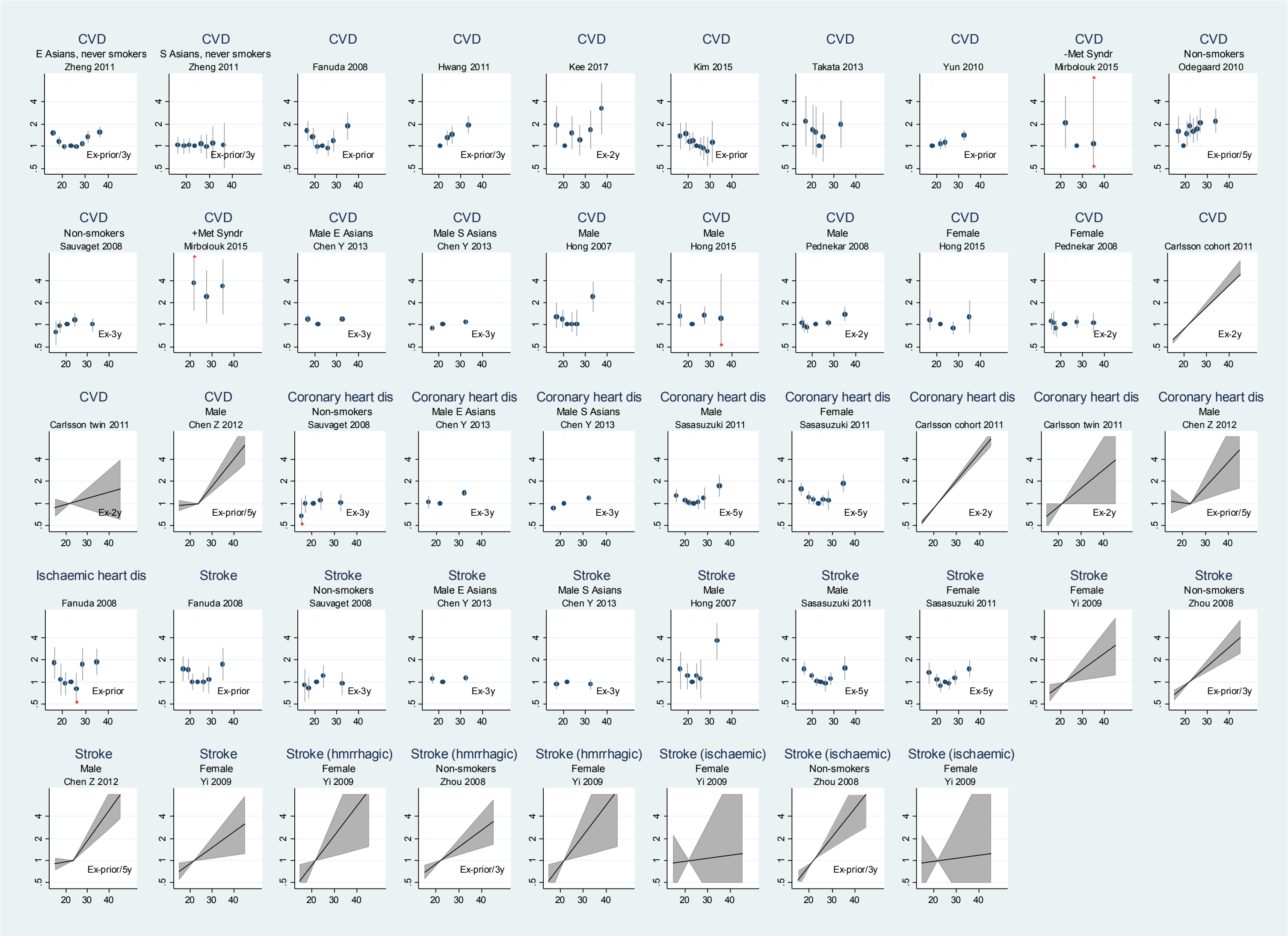

Notes: +Met Syndr and -Met Syndr = with and without metabolic syndrome respectively. Carlsson 2011 appears twice ("Carlsson cohort" and "Carlsson twin") as the authors presented separate results from a cohort approach and twin-matched approach).

Figure S4.6: Relative risk estimates of association between BMI and cancers mortality outcomes from studies in Asian settings

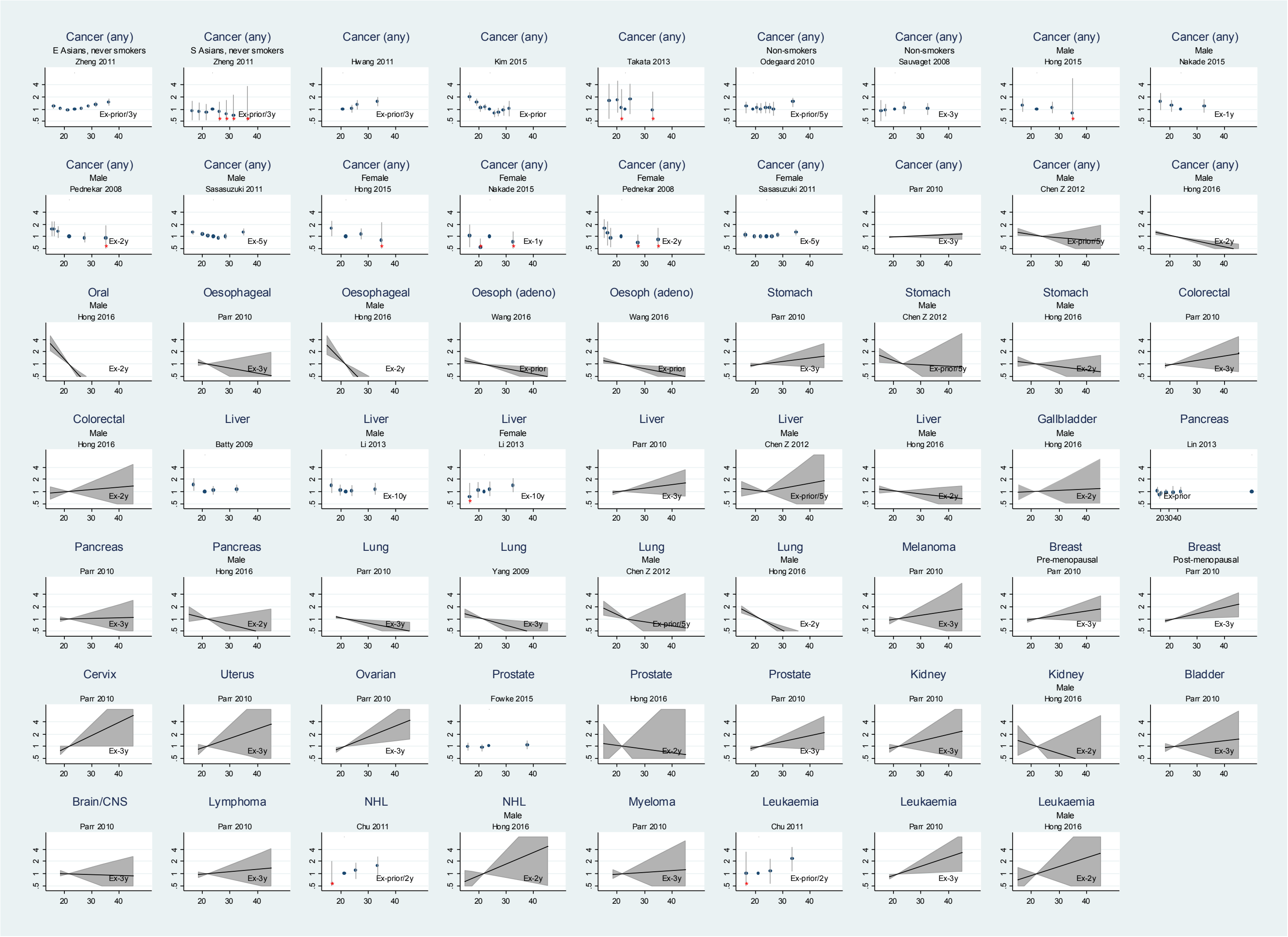

Figure S4.7: Relative risk estimates of association between BMI and mortality outcomes other than CVD/cancer from studies in Asian settings

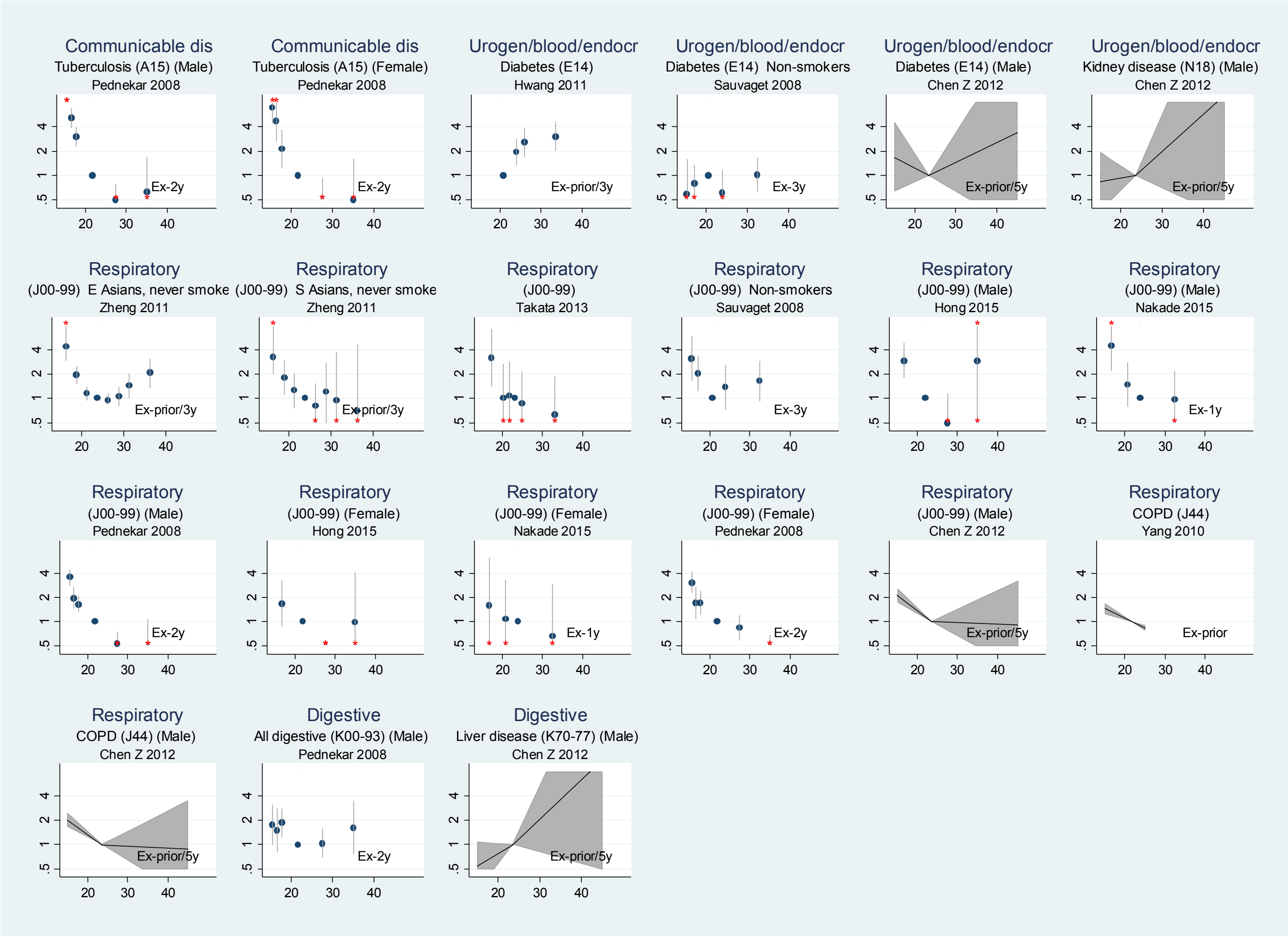

References for studies included in systematic review

1. Adabag S, Huxley RR, Lopez FL, et al. Obesity related risk of sudden cardiac death in the atherosclerosis risk in communities study. *Heart* 2015; **101**(3): 215-21. doi: 10.1136/heartjnl-2014-306238. Epub 2014 Nov 19.

2. Arnlov J, Ingelsson E, Sundstrom J, Lind L. Impact of body mass index and the metabolic syndrome on the risk of cardiovascular disease and death in middle-aged men. *Circulation* 2010; **121**(2): 230-6. doi: 10.1161/CIRCULATIONAHA.109.887521. Epub 2009 Dec 28.

3. Aune D, Sen A, Norat T, et al. Body Mass Index, Abdominal Fatness, and Heart Failure Incidence and Mortality: A Systematic Review and Dose-Response Meta-Analysis of Prospective Studies. *Circulation* 2016; **133**(7): 639-49. doi: 10.1161/CIRCULATIONAHA.115.016801. Epub 2016 Jan 8.

4. Batty GD, Shipley MJ, Kivimaki M, et al. Obesity and overweight in relation to liver disease mortality in men: 38 year follow-up of the original Whitehall study. *Int J Obes (Lond)* 2008; **32**(11): 1741-4. doi: 10.038/ijo.2008.162. Epub Sep 16.

5. Batty GD, Barzi F, Huxley R, et al. Obesity and liver cancer mortality in Asia: The Asia Pacific Cohort Studies Collaboration. *Cancer Epidemiol* 2009; **33**(6): 469-72. doi: 10.1016/j.canep.2009.09.010. Epub Nov 8.

6. Bessonova L, Marshall SF, Ziogas A, et al. The association of body mass index with mortality in the California Teachers Study. *Int J Cancer* 2011; **129**(10): 2492-501. doi: 10.1002/ijc.25905. Epub 2011 Apr 8.

7. Bethea TN, Kitahara CM, Sonderman J, et al. A pooled analysis of body mass index and pancreatic cancer mortality in african americans. *Cancer Epidemiol Biomarkers Prev* 2014; **23**(10): 2119-25. doi: 10.1158/055-9965.EPI-14-0422. Epub 2014 Jul 13.

8. Bombelli M, Facchetti R, Fodri D, et al. Impact of body mass index and waist circumference on the cardiovascular risk and all-cause death in a general population: data from the PAMELA study. *Nutr Metab Cardiovasc Dis* 2013; **23**(7): 650-6. doi: 10.1016/j.numecd.2012.01.004. Epub May 26.

9. Borrell LN, Samuel L. Body mass index categories and mortality risk in US adults: the effect of overweight and obesity on advancing death. *Am J Public Health* 2014; **104**(3): 512-9. doi: 10.2105/AJPH.013.301597. Epub 2014 Jan 16.

10. Cao Y, Ma J. Body mass index, prostate cancer-specific mortality, and biochemical recurrence: a systematic review and meta-analysis. *Cancer Prev Res (Phila)* 2011; **4**(4): 486-501. doi: 10.1158/940-6207.CAPR-10-0229. Epub 2011 Jan 13.

11. Carlsson S, Andersson T, de Faire U, Lichtenstein P, Michaelsson K, Ahlbom A. Body mass index and mortality: is the association explained by genetic factors? *Epidemiology* 2011; **22**(1): 98-103. doi: 10.1097/EDE.0b013e3181fce2a2.

12. Chen Y, Copeland WK, Vedanthan R, et al. Association between body mass index and cardiovascular disease mortality in east Asians and south Asians: pooled analysis of prospective data from the Asia Cohort Consortium. *BMJ* 2013; **347:f5446.**(doi): 10.1136/bmj.f5446.

13. Chen Z, Yang G, Offer A, et al. Body mass index and mortality in China: a 15-year prospective study of 220 000 men. *Int J Epidemiol* 2012; **41**(2): 472-81. doi: 10.1093/ije/dyr208. Epub 2012 Jan 31.

14. Chu DM, Wahlqvist ML, Lee MS, Chang HY. Central obesity predicts non-Hodgkin's lymphoma mortality and overall obesity predicts leukemia mortality in adult Taiwanese. *J Am Coll* ***Nutr*** **2011; 30(5): 310-9.**

15. Cohen SS, Park Y, Signorello LB, et al. A pooled analysis of body mass index and mortality among African Americans. *PLoS One* 2014; **9**(11): e111980. doi: 10.1371/journal.pone.0111980. eCollection 2014.

16. Crump C, Sundquist J, Winkleby MA, Sundquist K. Interactive Effects of Aerobic Fitness, Strength, and Obesity on Mortality in Men. *Am J Prev Med* 2017; **52**(3): 353-61. doi: 10.1016/j.amepre.2016.10.002. Epub Nov 14.

17. Czernichow S, Kengne AP, Stamatakis E, Hamer M, Batty GD. Body mass index, waist circumference and waist-hip ratio: which is the better discriminator of cardiovascular disease mortality risk?: evidence from an individual-participant meta-analysis of 82 864 participants from nine cohort studies. *Obes Rev* 2011; **12**(9): 680-7. doi: 10.1111/j.467-789X.2011.00879.x. Epub 2011 Apr 27.

18. de Hollander EL, Van Zutphen M, Bogers RP, Bemelmans WJ, De Groot LC. The impact of body mass index in old age on cause-specific mortality. *J Nutr Health Aging* 2012; **16**(1): 100-6.

19. Dehal A, Garrett T, Tedders SH, Arroyo C, Afriyie-Gyawu E, Zhang J. Body mass index and death rate of colorectal cancer among a national cohort of U.S. adults. *Nutr Cancer* 2011; **63**(8): 1218-25. doi: 10.080/01635581.2011.607539. Epub 2011 Oct 7.

20. Dudina A, Cooney MT, Bacquer DD, et al. Relationships between body mass index, cardiovascular mortality, and risk factors: a report from the SCORE investigators. *Eur J Cardiovasc Prev Rehabil* 2011; **18**(5): 731-42. doi: 10.1177/1741826711412039. Epub 2011 Jun 3.

21. Eranti A, Aro AL, Kerola T, et al. Body Mass Index as a Predictor of Sudden Cardiac Death and Usefulness of the Electrocardiogram for Risk Stratification. *Am J Cardiol* 2016; **117**(3): 388-93. doi: 10.1016/j.amjcard.2015.10.057. Epub Nov 18.

22. Faeh D, Braun J, Tarnutzer S, Bopp M. Obesity but not overweight is associated with increased mortality risk. *Eur J Epidemiol* 2011; **26**(8): 647-55. doi: 10.1007/s10654-011-9593-2. Epub 2011 Jun 17.

23. Fowke JH, McLerran DF, Gupta PC, et al. Associations of body mass index, smoking, and alcohol consumption with prostate cancer mortality in the Asia Cohort Consortium. *Am J Epidemiol* 2015; **182**(5): 381-9. doi: 10.1093/aje/kwv089. Epub 2015 Aug 4.

24. Funada S, Shimazu T, Kakizaki M, et al. Body mass index and cardiovascular disease mortality in Japan: the Ohsaki Study. *Prev Med* 2008; **47**(1): 66-70. doi: 10.1016/j.ypmed.2008.03.010. Epub Mar 26.

25. Genkinger JM, Kitahara CM, Bernstein L, et al. Central adiposity, obesity during early adulthood, and pancreatic cancer mortality in a pooled analysis of cohort studies. *Ann Oncol* 2015; **26**(11): 2257-66. doi: 10.1093/annonc/mdv355. Epub 2015 Sep 7.

26. Ghaem Maralani H, Tai BC, Wong TY, et al. The prognostic role of body mass index on mortality amongst the middle-aged and elderly: a competing risk analysis. *Diabetes Res Clin Pract* 2014; **103**(1): 42-50. doi: 10.1016/j.diabres.2013.11.025. Epub Dec 12.

27. Global BMIMC, Di Angelantonio E, Bhupathiraju Sh N, et al. Body-mass index and all-cause mortality: individual-participant-data meta-analysis of 239 prospective studies in four continents. *Lancet* 2016; **388**(10046): 776-86.

28. Gray L, Lee IM, Sesso HD, Batty GD. Association of body mass index in early adulthood and middle age with future site-specific cancer mortality: the Harvard Alumni Health Study. *Ann Oncol* 2012; **23**(3): 754-9. doi: 10.1093/annonc/mdr270. Epub 2011 Jun 15.

29. Han X, Stevens J, Truesdale KP, et al. Body mass index at early adulthood, subsequent weight change and cancer incidence and mortality. *Int J Cancer* 2014; **135**(12): 2900-9. doi: 10.1002/ijc.28930. Epub 2014 May 8.

30. Hart CL, Gruer L, Watt GC. Cause specific mortality, social position, and obesity among women who had never smoked: 28 year cohort study. *BMJ* 2011; **342:d3785.**(doi): 10.1136/bmj.d3785.

31. Hong JS, Yi SW, Kang HC, Ohrr H. Body mass index and mortality in South Korean men resulting from cardiovascular disease: a Kangwha cohort study. *Ann Epidemiol* 2007; **17**(8): 622-7. Epub 2007 Jun 6.

32. Hong S, Yi SW, Sull JW, Hong JS, Jee SH, Ohrr H. Body mass index and mortality among Korean elderly in rural communities: Kangwha Cohort Study. *PLoS One* 2015; **10**(2): e0117731. doi: 10.1371/journal.pone.. eCollection 2015.

33. Hong JS, Yi SW, Yi JJ, Hong S, Ohrr H. Body Mass Index and Cancer Mortality Among Korean Older Middle-Aged Men: A Prospective Cohort Study. *Medicine (Baltimore)* 2016; **95**(21): e3684. doi: 10.1097/MD.0000000000003684.

34. Hwang LC, Chen SC, Chen CJ. Increased risk of mortality from overweight and obesity in middle-aged individuals from six communities in Taiwan. *J Formos Med Assoc* 2011; **110**(5): 290-8. doi: 10.1016/S0929-6646(11)60044-2.

35. Jia T, Tynelius P, Rasmussen F. U-shaped association of body mass index in early adulthood with unintentional mortality from injuries: a cohort study of Swedish men with 35 years of follow-up. *Int J Obes (Lond)* 2016; **40**(5): 809-14. doi: 10.1038/ijo.2015.239. Epub Nov 26.

36. Kee CC, Sumarni MG, Lim KH, et al. Association of BMI with risk of CVD mortality and all-cause mortality. *Public Health Nutr* 2017; **20**(7): 1226-34. doi: 10.017/S136898001600344X. Epub 2017 Jan 12.

37. Kim NH, Lee J, Kim TJ, et al. Body Mass Index and Mortality in the General Population and in Subjects with Chronic Disease in Korea: A Nationwide Cohort Study (2002-2010). *PLoS One* 2015; **10**(10): e0139924. doi: 10.1371/journal.pone.. eCollection 2015.

38. Kivimaki M, Ferrie JE, Batty GD, et al. Optimal form of operationalizing BMI in relation to all-cause and cause-specific mortality: the original Whitehall study. *Obesity (Silver Spring)* 2008; **16**(8): 1926-32. doi: 10.038/oby.2008.322. Epub Jun 12.

39. Klenk J, Nagel G, Ulmer H, et al. Body mass index and mortality: results of a cohort of 184,697 adults in Austria. *Eur J Epidemiol* 2009; **24**(2): 83-91. doi: 10.1007/s10654-009-9312-4. Epub 2009 Jan 29.

40. Korkeila M, Rissanen A, Sorensen TI, Kaprio J. BMI, weight stability and mortality among adults without clinical co-morbidities: a 22-year mortality follow-up in the Finnish twin cohort. *Obes Facts* 2009; **2**(6): 344-51. doi: 10.1159/000261416. Epub 2009 Dec 4.

41. Leitzmann MF, Moore SC, Koster A, et al. Waist circumference as compared with body-mass index in predicting mortality from specific causes. *PLoS One* 2011; **6**(4): e18582. doi: 10.1371/journal.pone.0018582.

42. Li Y, Yatsuya H, Yamagishi K, et al. Body mass index and weight change during adulthood are associated with increased mortality from liver cancer: the JACC Study. *J Epidemiol* 2013; **23**(3): 219-26. Epub 2013 Apr 20.

43. Lin Y, Fu R, Grant E, et al. Association of body mass index and risk of death from pancreas cancer in Asians: findings from the Asia Cohort Consortium. *Eur J Cancer Prev* 2013; **22**(3): 244-50. doi: 10.1097/CEJ.0b013e3283592cef.

44. Ma J, Flanders WD, Ward EM, Jemal A. Body mass index in young adulthood and premature death: analyses of the US National Health Interview Survey linked mortality files. *Am J Epidemiol* 2011; **174**(8): 934-44. doi: 10.1093/aje/kwr169. Epub 2011 Aug 26.

45. Mirbolouk M, Asgari S, Sheikholeslami F, Mirbolouk F, Azizi F, Hadaegh F. Different obesity phenotypes, and incident cardiovascular disease and mortality events in elderly Iranians: Tehran lipid and glucose study. *Geriatr Gerontol Int* 2015; **15**(4): 449-56. doi: 10.1111/ggi.12295. Epub 2014 Apr 21.

46. Nakade M, Takagi D, Suzuki K, et al. Influence of socioeconomic status on the association between body mass index and cause-specific mortality among older Japanese adults: The AGES Cohort Study. *Prev Med* 2015; **77:112-8.**(doi): 10.1016/j.ypmed.2015.05.015. Epub May 30.

47. Odegaard AO, Pereira MA, Koh WP, et al. BMI, all-cause and cause-specific mortality in Chinese Singaporean men and women: the Singapore Chinese health study. *PLoS One* 2010; **5**(11): e14000. doi: 10.1371/journal.pone.0014000.

48. Park Y, Wang S, Kitahara CM, et al. Body mass index and risk of death in Asian Americans. *Am J Public Health* 2014; **104**(3): 520-5. doi: 10.2105/AJPH.013.301573. Epub 2014 Jan 16.

49. Parr CL, Batty GD, Lam TH, et al. Body-mass index and cancer mortality in the Asia-Pacific Cohort Studies Collaboration: pooled analyses of 424,519 participants. *Lancet Oncol* 2010; **11**(8): 741-52. doi: 10.1016/S470-2045(10)70141-8. Epub 2010 Jun 30.

50. Paulsen J, Askim A, Mohus RM, et al. Associations of obesity and lifestyle with the risk and mortality of bloodstream infection in a general population: a 15-year follow-up of 64 027 individuals in the HUNT Study. *Int J Epidemiol* 2017; **15**(10).

51. Pednekar MS, Hakama M, Hebert JR, Gupta PC. Association of body mass index with all-cause and cause-specific mortality: findings from a prospective cohort study in Mumbai (Bombay), India. *Int J Epidemiol* 2008; **37**(3): 524-35. doi: 10.1093/ije/dyn001. Epub 2008 Feb 14.

52. Prospective Studies Collaboration. Body-mass index and cause-specific mortality in 900 000 adults: collaborative analyses of 57 prospective studies. *Lancet* 2009; **373**(9669): 1083-96. doi: 10.16/S0140-6736(09)60318-4. Epub 2009 Mar 18.

53. Reeves GK, Pirie K, Beral V, Green J, Spencer E, Bull D. Cancer incidence and mortality in relation to body mass index in the Million Women Study: cohort study. *BMJ* 2007; **335**(7630): 1134. Epub 2007 Nov 6.

54. Sasazuki S, Inoue M, Tsuji I, et al. Body mass index and mortality from all causes and major causes in Japanese: results of a pooled analysis of 7 large-scale cohort studies. *J Epidemiol* 2011; **21**(6): 417-30. Epub 2011 Sep 10.

55. Sauvaget C, Ramadas K, Thomas G, Vinoda J, Thara S, Sankaranarayanan R. Body mass index, weight change and mortality risk in a prospective study in India. *Int J Epidemiol* 2008; **37**(5): 990-1004. doi: 10.93/ije/dyn059. Epub 2008 Apr 3.

56. Song X, Pitkaniemi J, Gao W, et al. Relationship between body mass index and mortality among Europeans. *Eur J Clin Nutr* 2012; **66**(2): 156-65. doi: 10.1038/ejcn.2011.145. Epub Aug 10.

57. Staiano AE, Reeder BA, Elliott S, et al. Body mass index versus waist circumference as predictors of mortality in Canadian adults. *Int J Obes (Lond)* 2012; **36**(11): 1450-4. doi: 10.038/ijo.2011.268. Epub 2 Jan 17.

58. Taghizadeh N, Boezen HM, Schouten JP, Schroder CP, Elisabeth de Vries EG, Vonk JM. BMI and lifetime changes in BMI and cancer mortality risk. *PLoS One* 2015; **10**(4): e0125261. doi: 10.1371/journal.pone.. eCollection 2015.

59. Takata Y, Ansai T, Soh I, et al. Body mass index and disease-specific mortality in an 80-year-old population at the 12-year follow-up. *Arch Gerontol Geriatr* 2013; **57**(1): 46-53. doi: 10.1016/j.archger.2013.02.006. Epub Mar 9.

60. Wang SM, Fan JH, Jia MM, et al. Body mass index and long-term risk of death from esophageal squamous cell carcinoma in a Chinese population. *Thorac Cancer* 2016; **7**(4): 387-92. doi: 10.1111/759-7714.12340. Epub 2016 Apr 7.

61. Yang L, Yang G, Zhou M, et al. Body mass index and mortality from lung cancer in smokers and nonsmokers: a nationally representative prospective study of 220,000 men in China. *Int J Cancer* 2009; **125**(9): 2136-43. doi: 10.1002/ijc.24527.

62. Yang L, Zhou M, Smith M, et al. Body mass index and chronic obstructive pulmonary disease-related mortality: a nationally representative prospective study of 220,000 men in China. *Int J Epidemiol* 2010; **39**(4): 1027-36. doi: 10.93/ije/dyq051. Epub 2010 Apr 16.

63. Yi SW, Odongua N, Nam CM, Sull JW, Ohrr H. Body mass index and stroke mortality by smoking and age at menopause among Korean postmenopausal women. *Stroke* 2009; **40**(11): 3428-35. doi: 10.1161/STROKEAHA.109.555144. Epub 2009 Aug 20.

64. Yun KE, Park HS, Song YM, Cho SI. Increases in body mass index over a 7-year period and risk of cause-specific mortality in Korean men. *Int J Epidemiol* 2010; **39**(2): 520-8. doi: 10.1093/ije/dyp282. Epub 2009 Sep 17.

65. Zajacova A, Burgard SA. Shape of the BMI-mortality association by cause of death, using generalized additive models: NHIS 1986-2006. *J Aging Health* 2012; **24**(2): 191-211. doi: 10.1177/0898264311406268. Epub 2011 May 10.

66. Zheng W, McLerran DF, Rolland B, et al. Association between body-mass index and risk of death in more than 1 million Asians. *N Engl J Med* 2011; **364**(8): 719-29. doi: 10.1056/NEJMoa1010679.

67. Zhou M, Offer A, Yang G, et al. Body mass index, blood pressure, and mortality from stroke: a nationally representative prospective study of 212,000 Chinese men. *Stroke* 2008; **39**(3): 753-9. doi: 10.1161/STROKEAHA.107.495374. Epub 2008 Jan 31.
